# Supplementary material for: Discovery of a metabolic alternative to the classical mevalonate pathway
Source: eLife. 2013 Dec 10;2:e00672. doi: 10.7554/eLife.00672 (PMC3857490; doi:10.7554/eLife.00672)
Supplement: Figure 2—source data 1. — DOI: http://dx.doi.org/10.7554/eLife.00672.005 [file elife00672s001.rtf]

Figure 2 - Source data 1. Alignment of IPKs from the three domains of life

Anolis_caroliensis-lizard/1-281            --------------------------------------------MAAAVDCIVKLGGGAL
Callorhinchus_milii-shark/1-249            ------------------------------------------------LDCVVKLGGSAV
Saccoglossus_kowalevskii/1-283             ----------------------------------------------MNIECIIKLGGSAI
Branchiostoma_floridae/1-298               --------------------------------MVHKCCLCQCTDVLSGLECIVKLGGSAV
Strongylocentrotus_purpuratus-urchin/1-280 --------------------------------------------MEQKIECIIKVGGSAI
Paracentrotus_lividus-sea_star/1-236       --------------------------------------------MEHKIECIIKLGGSAI
Lottia_gigantea/1-283                      -------------------------------------------MTENDIDLIIKFGGSSI
Nematostella-anemone/1-278                 ----------------------------------------------MPLEVAIKLGGCAI
Acropora_palmata/1-176                     ------------------------------------------------------------
Montastraea_faveolata/1-203                ------------------------------------------------------------
Trichoplax_adhaerens/1-281                 -----------------------------------------MALENRHVDCIIKLGGSAI
Rhizopus_oryzae/1-279                      ------------------------------------------------MLVIVKLGGAAI
Mucor_circinelloides/1-306                 ----------------------------------------------MSKIVIVKLGGAAI
Epichloe_festucae/1-297                    -----------------------------------------------MAIVIVKIGGAAI
Spizellomyces_punctatus/1-299              ---------------------------------------------MTSIDLIIKIGGSVL
Phaeodactylum_tricornutum/1-361            MPQCCRKLVWVLYALAVVSHATVCYSEEQQSSPEATTCFSPSSSFADHDVVLVKIGGSSI
Thalassiosira_pseudonana/1-355             ----------------------MPKPGAHVQNEQPREEAPKKRVEEKEKIIILKIGGSSI
Perkinsus_marinus/1-320                    -----------MSSSSSKVIGGCVAVATTVACGIAAAMVYSRRRRGTKCTCVIKIGGSAC
Polysphondylium_pallidum/1-283             ------------------------------------------MNRKDYKLLIIKCGGAYL
Dictyostelium_discoideum/1-292             ------------------------------------------MQENKQQLIIIKFGGAYI
Arabidopsis_thaliana/1-332                 ------------------------------------MELNISESRSRSIRCIVKLGGAAI
Rice/1-335                                 ------------------------------MAEEAAQEQQTDPAASRPVRCIVKLGGAAI
Maize/1-340                                ------------------------------MAEE-MAQAQPRLTAPRSVRCIVKLGGAAI
Poplar/1-335                               ------------------------------------MEDTTTLSVTKPIRCIVKLGGAAI
Ricinus_communis/1-337                     ------------------------------------MEDTAALNLTKPIRCIVKLGGAAI
Vitis_vinifera/1-340                       ---------------------------MDDGISRNNNNSNQILCPIKPIRCIVKLGGAAI
Soybean/1-335                              --------------------------MEQHKNESHTQTSLPLSPFTQPIRCIVKLGGAAI
Selaginella_moellendorffii/1-312           ---------------------------------------MESSSRKHHVRCIVKLGGAAI
Physcomitrella_patens.2/1-351              -----------------MIPAQFPTPPESQALNIATSQFSALANSHRYVHCIVKLGGAAI
Chlamydomonas_reinhardtii/1-344            --------------------------MKPVLTTQPLAAVAPGPSAAPPVRRIIKLGGAAV
Volvox_carteri/1-321                       ------------------------------------------PPAPAPVRRIIKLGGAAI
Micromonas_pusilla.CCMP1545/1-411          --------------------MPPKRARETAQSVAQDSRVDQDLRCKIPVALIVKLGGAAI
Micromonas_pusilla.RCC299/1-402            ----------------MRTKGEKRKRIVARKQAPVRWVTRGAPPADGSVDVIVKFGGAAI
Ostreococcus_lucimarinus/1-352             ----------------------------------------------MPSVAVVKFGGAVL
Ostreococcus_tauri/1-356                   ----------------------------------------MALDVPIRRVVVVKLGGAAI
Entamoeba_hisolytica/1-259                 -------------------------------------------MNSIPNLIILKIGGSYL
Entamoeba_invadens/1-257                   -------------------------------------------MDT-P-LFIIKIGGGFL
Roseiflexus_castenholzii/1-269             ------------------------------------------------MITFIKWGGSVI
Herpetosiphon_aurantiacus/1-267            ----------------------------------------------MNKPIFIKLGGSML
Desulfurococcus_kamchatkensis/1-262        -------------------------------------------MTPLQDTVYVKLGGSFI
Thermosphaera_aggregans/1-261              ---------------------------------------------MTRDVVFLKAGGSFI
Staphylothermus_hellenicus/1-266           ----------------------------------------MSINRDHDNVVFVKLGGSFI
Hyperthermus_butylicus/1-260               --------------------------------------MPRGGAGRQGGVYVVKLGGSVV
Aeropyrum_pernix/1-249                     --------------------------------------MASPGSRAPCGSAVVKLGGGLI
Ignicoccus_hospitalis/1-236                -------------------------------------------------MIVIKLGGSVI
Thermofilum_pendens/1-259                  --------------------------------------------MNPAMLTVIKLGGSVI
Methanocaldococcus_jannaschii/1-260        ------------------------------------------------MLTILKLGGSIL
Methanococcus_maripaludis/1-257            ------------------------------------------------MFAILKLGGSIL
Metallosphaera_sedula/1-224                ---------------------------------------------MGKPNRVIKLGGSAI
Sulfolobus_solfataricus/1-241              -------------------------------------MEMDMGSELGYDYRVLKLGGSLI
Archaeoglobus_profundus/1-239              -------------------------------------------------MIVVKIGGSAI
Methanospirillum_hungatei/1-256            ----------------------------------------------MPDRIILKLGGSVI
Methanosarcina_mazei/1-260                 -------------------------------------------MNASNEPVILKLGGSAI
Methanosaeta_thermophila/1-248             ------------------------------------------------MLKILKLGGSII
Nitrosopumilus_maritimus/1-247             -------------------------------------------------MILIKLGGSII
Cenarchaeum_symbiosum/1-246                ---------------------------------------------------MVKLGGSVI
Ferroplasma_acidarmanus/1-280              -----------MLFKNMLSVLLNLDLLFNQILTTDAIVKFNNTFYIIYTMIVIKIGGSII
Picrophilus_torridus/1-242                 -------------------------------------------------MIIVKLGGSVI
Pyrococcus_furiosus/1-256                  -------------------------------------------------MILVKIGGSVI
Thermococcus_barophilus/1-266              -------------------------------------------------MIIIKLGGSVI
Chloroflexus_aggregans/1-268               ------------------------------------------------MYTFVKFGGSVI
Pseudomonas_syringae/1-261                 ---------------------------------------------MKPACYVIKLGGSVT
Shewanella_denitrificans/1-279             --------------------------------------------MELDACLIIKFGGSII
Streptomyces_wedmorensis__fomA/1-266       ---------------------------------------------MTPDFLAIKVGGSLF

Anolis_caroliensis-lizard/1-281            THKK--QLETPKLEALRRAAALVGKLYGA-------------------------------
Callorhinchus_milii-shark/1-249            TVKD--ELETLRVGELRRAAALISRLCQE-------------------------------
Saccoglossus_kowalevskii/1-283             TEKD--SFETYNLAAIQQAAKIISEIK---------------------------------
Branchiostoma_floridae/1-298               TDKS--TLETPRLDAIRAAADIISQVR---------------------------------
Strongylocentrotus_purpuratus-urchin/1-280 TYKT--ELETANPENIVKTAELLVLSR---------------------------------
Paracentrotus_lividus-sea_star/1-236       TQKS--ELETANMENIQKAVELLVLSR---------------------------------
Lottia_gigantea/1-283                      TNKD--CIETLRVTALEWCAHLVKKCLNS-------------------------------
Nematostella-anemone/1-278                 TDKN--TFETFDLSSIEAAAKVVSKLV---------------------------------
Acropora_palmata/1-176                     ------------------------------------------------------------
Montastraea_faveolata/1-203                ------------------------------------------------------------
Trichoplax_adhaerens/1-281                 TSKQ--HLEKANTQAINIAASHVHE-----------------------------------
Rhizopus_oryzae/1-279                      TNKKG-VCEYS--ACLDRLLDQVRQAYCDLQ-----------------------------
Mucor_circinelloides/1-306                 TNKKG-ICELAPENNLSVLLDQVATAYEILK-----------------------------
Epichloe_festucae/1-297                    TVKSKADTLSPDLDTLVGGIAQVCRDELRPR-----------------------------
Spizellomyces_punctatus/1-299              TDKTS-YETLSPRSLLDPLFDSISNLHAT-------------------------------
Phaeodactylum_tricornutum/1-361            TNKA--QKETLNQEALDWFAEAVRPVLSRNFCA----PSGSDDIVTGRIHNNESALAK--
Thalassiosira_pseudonana/1-355             THKG--EEETLNTESLNWFARLIASSVNESFLS----SSGTYNNVDSSR-----------
Perkinsus_marinus/1-320                    TKKD--EFETIDDGVLSKTAEQLVEAIER-------------------------------
Polysphondylium_pallidum/1-283             TKKD--QHQQLNQDNFNSLLDILDSLLKKN------------------------------
Dictyostelium_discoideum/1-292             SKKD--KLETIWKENIDNLVYIIKTLSIDYN-----------------------------
Arabidopsis_thaliana/1-332                 TCKN--ELEKIHDENLEVVACQLRQAMLEGSAPS---KVIGMDWSKRPGSSEIS-CDVDD
Rice/1-335                                 TNKG--ELESIDAASLRSACAQLRQAMSHGGAAG---KVVGMDWSRRPGDPTGPVVDVEG
Maize/1-340                                TNKG--ELESINEENLRSACAQLRHAMSESDGDGAMEKVLGMDWSRKPGDPVDPAVDAEW
Poplar/1-335                               TCKN--DLEKINEENVEIVSSQLREAMITGSSSR---KVLGMDWSKRPGKSGIS-CDADD
Ricinus_communis/1-337                     TCKN--ELEKVNEENLEIVSSQLKKALIAGSGSQ---RVLGMDWSNRHGTSEIS-CDVDN
Vitis_vinifera/1-340                       TCKN--EIEKMNEESIEKVSSQLRQAMISGLSSL---KICGMDWSKRPGNSEAS-PTVDD
Soybean/1-335                              TSKN--ELEMINEEILQKVSEQLREAMVASSE-----KPPGMDWSKRPGASEIF-CNPEE
Selaginella_moellendorffii/1-312           TFKD--KLEALNRESLSKTSLQLHEAMGGGVSFN-------MDWSKNLEVP-------DR
Physcomitrella_patens.2/1-351              TRKD--ELETVNDPVLSATTLHLREAM--GLSY----TNVSMDWSRRNGSSIVDLVPN--
Chlamydomonas_reinhardtii/1-344            TVKS--QLETLRPEVLDSLVRTLATTS--GLPEA--------------------------
Volvox_carteri/1-321                       THKS--QLETLQQDVLRRVCVSLANE---GHSSS-----------------SSASPSSSS
Micromonas_pusilla.CCMP1545/1-411          TIKD--GRENLDEKTLDACVASIAAVHEHECKLQ--------------------------
Micromonas_pusilla.RCC299/1-402            TVKDG-EPDTLNDEALGACCASIAEVVRTEISEL-------------------------A
Ostreococcus_lucimarinus/1-352             TDKR--QRGVVDARGLADCARVARLARD--------------------------------
Ostreococcus_tauri/1-356                   TDKR--SSRKIDAPGLRACASVVREATA--------------------------------
Entamoeba_hisolytica/1-259                 TEKNR-ADGPPILENIHMFSKCLAQFIHS-------------------------------
Entamoeba_invadens/1-257                   TEKVG-ENGAVYLDKIHLFCKVLSSYKQS-------------------------------
Roseiflexus_castenholzii/1-269             TDKT--QQERADAGTIRRLAGELRQALDAAP-----------------------------
Herpetosiphon_aurantiacus/1-267            TDKT--TAERLVDQTLKQVVTDLSAWRQAHP-----------------------------
Desulfurococcus_kamchatkensis/1-262        TIKD--KPVTLRRESLDAVAEILRQVYGR-------------------------------
Thermosphaera_aggregans/1-261              TFKD--KPVSVNYMALEALAEALKATHR--------------------------------
Staphylothermus_hellenicus/1-266           TYKD--KPYSINYAALKKTVDILSSVYG--------------------------------
Hyperthermus_butylicus/1-260               TVKD--KPFTPNLDILHHLARVLAGLNREG------------------------------
Aeropyrum_pernix/1-249                     TFKD--KPYTIDRAMLERTASQLAAYSKGT------------------------------
Ignicoccus_hospitalis/1-236                SNKK--VPYSLNKELIGKIGNSLREYAG--------------------------------
Thermofilum_pendens/1-259                  TDKS--KPYTVRSSNLKTASAALAKLYGE-------------------------------
Methanocaldococcus_jannaschii/1-260        SDKN--VPYSIKWDNLERIAMEIKNALDYYKNQ---------------------------
Methanococcus_maripaludis/1-257            CDKN--IPYSINWENLENIAIEIKEAIEYYKSK---------------------------
Metallosphaera_sedula/1-224                TCKA--VPYCADLPVIRQIAGEIKDFVDG-------------------------------
Sulfolobus_solfataricus/1-241              TCKD--VPRCVKLEVLRRVSEEIRKFVNENP-----------------------------
Archaeoglobus_profundus/1-239              TDKK--GFKIVKIDSIERVAKDIAEVRPR-------------------------------
Methanospirillum_hungatei/1-256            TDKERGDAGVIRESVLQEVARALKEYAD--------------------------------
Methanosarcina_mazei/1-260                 TDKGA-YEGVVKEADLLRIAQEVSGFRG--------------------------------
Methanosaeta_thermophila/1-248             TDKS--RLATARLDQISRIAHEISGIE---------------------------------
Nitrosopumilus_maritimus/1-247             TNKE--KPLSARRKTIDNLAKSLKKIQE--------------------------------
Cenarchaeum_symbiosum/1-246                TNKE--KPLSARTRNMDGIAAALAKLRE--------------------------------
Ferroplasma_acidarmanus/1-280              TDKS--MYKKFNGQIVENIVKTLKRIDK--------------------------------
Picrophilus_torridus/1-242                 TDKS--YYRKFRSGAVKKISKVLSDFN---------------------------------
Pyrococcus_furiosus/1-256                  SDKT--RKFHFRAEVVKRIAYEISRFFPE-------------------------------
Thermococcus_barophilus/1-266              SDKE--KEYSFHKYIVEQIAEEIAQFYPE-------------------------------
Chloroflexus_aggregans/1-268               TDKT--DRESADLAVIEALAGAVAAARAADP-----------------------------
Pseudomonas_syringae/1-261                 TYKQ--GRYALRHDAIALVAPCLR-LGRRS------------------------------
Shewanella_denitrificans/1-279             TDRE--SPYTLKRDMCNALIQKVIALKQQS------------------------------
Streptomyces_wedmorensis__fomA/1-266       SRKD--EPGSLDDDAVTPFARNFARLAETY------------------------------

Anolis_caroliensis-lizard/1-281            -----------GERRCIVVHGAGSFGHFQAKQHNVASG--TSEGSAAS-------LSLRQ
Callorhinchus_milii-shark/1-249            -----------G-KRAVVVHGAGXXXXXXXXXXXXXXXVKAFETPSRRESR----RKIVI
Saccoglossus_kowalevskii/1-283             -------------GRCVLVHGAGSFGHFQAKEYKVSNGYCANLDKDYI-------ENVKH
Branchiostoma_floridae/1-298               -------------GRCIVVHGAGSFGHFQAREHGVVWGYRDKETDTEA-------QTVKL
Strongylocentrotus_purpuratus-urchin/1-280 -----------LKGKCILVHGAGSFGHFQASEYGVAKGYTSLS--PDE-------TIVKE
Paracentrotus_lividus-sea_star/1-236       -----------VQGKCILVHGAGSFGHFQACEYGVSKGHASLP--PGD-------TRVKE
Lottia_gigantea/1-283                      -----------D-KNCVIVHGAGSFGHHQAKEYHVSSGYSHLSTKFET-------DKIKL
Nematostella-anemone/1-278                 -------------GKCVLIHGAGSFGHFQAHKYGIAKGLGDDPIRG------------KA
Acropora_palmata/1-176                     ------------------------------------------------------------
Montastraea_faveolata/1-203                ------------------------------------------------------------
Trichoplax_adhaerens/1-281                 -----------MTRKCVIVHGAGSFGHFHAKKYNIATG--FNDTDFEQ---------QRI
Rhizopus_oryzae/1-279                      ----------AQGHQLILVHGAGSFGHPQAKKYQLKEGWRTSRPGAD----------YLK
Mucor_circinelloides/1-306                 ----------GAGHQLILVHGAGSFGHPQAVKYNLKSGWSASASSSTDALSN---QNYLK
Epichloe_festucae/1-297                    ------------GTRLILIHGAGSFGHPPAKKYRVKAGWSTTRPNERTSVN----ESVKF
Spizellomyces_punctatus/1-299              -------------TRFLLIHGAGSFGHPHAHAAGLSIGFTPPSPK------------HVL
Phaeodactylum_tricornutum/1-361            ----------GRPIAFVVVHGAGSFGHHQAKEFGLQGQTAAAPTEAAVDEKQR--RFAMQ
Thalassiosira_pseudonana/1-355             -----------SKPKFIVVHGAGSFGHHSAKRYGLRCGKAVYIDELGTAVTGTSQQYQME
Perkinsus_marinus/1-320                    -----------SNARPMLIHGAGSFGHFQAKDYAVSRGNKELSGKPEGGSAYAMSRFLSK
Polysphondylium_pallidum/1-283             -------------YKLILIIGAGSFGHHEAKQYKITGGFNYNFDEVNDVVDS------RK
Dictyostelium_discoideum/1-292             -------------HKIILIIGAGSFGHHSANQYKVKYGLSSNCGDNKF---------LCE
Arabidopsis_thaliana/1-332                 -IGDQKS---SEFSKFVVVHGAGSFGHFQASRSGVHKGGLEKPIV-------------KA
Rice/1-335                                 LSEMGGL--GLDS-NFVVVHGAGSFGHFQASRSGVHKGGLHSTLV-------------KA
Maize/1-340                                IAGIARL--GLDT-NFVVVHGAGSFGHFQASRSGVHKGGLHSTLV-------------KA
Poplar/1-335                               --FEDQN---LDSSSFVVVHGAGSFGHFQASKSGVHKGGLNKPLV-------------KA
Ricinus_communis/1-337                     -FQEQTS---LDSCSFVVVHGAGSFGHFQASKSGVHKGGLSQPLV-------------KA
Vitis_vinifera/1-340                       -FSDQSL---LDSDRFIVVHGAGSFGHFQASKSGVHKGGLNQPLV-------------KA
Soybean/1-335                              -FGDHSA---IDCSPFIVVHGAGSFGHFQASKSGVHKGQLNKPLV-------------KG
Selaginella_moellendorffii/1-312           I--DMEL---PQKRAFVVVHGAGSFGHFQASISGVNKGGLDNSLLV------------NA
Physcomitrella_patens.2/1-351              -STDQHL--GFQN-PFVVVHGAGSYGHFQASKSGVNKGDLKNPLV-------------KA
Chlamydomonas_reinhardtii/1-344            ---EAST---SGAGGTVLVHGAGSFGHFPASEYGVVRGPISDPRV-------------RR
Volvox_carteri/1-321                       SPSSSSG---GSDGGTVLVHGAGSFGHHPASEYGVARGNLSDPRV-------------RL
Micromonas_pusilla.CCMP1545/1-411          AE--KPN---YVRKGIVVVHGAGSYGHGVAKDFLVAEGTRAAMRDNRQ---------LGV
Micromonas_pusilla.RCC299/1-402            IEFAKES---PLR--VIVVHGAGSFGHPQAKQYGVADGGDMDGDPV-----------LRE
Ostreococcus_lucimarinus/1-352             -----------AGASLIIAHGAGSFGHAEAKACGCARGGDLRDAA------------FAL
Ostreococcus_tauri/1-356                   -----------RGETLIVAHGAGSFGHAEAAACGCARGGDVEDET------------FAR
Entamoeba_hisolytica/1-259                 ----------HPKQPIILAHGAGSFGHIPAATYHLAEGFH------------------KT
Entamoeba_invadens/1-257                   ----------HPTHRFILVHGAGSFGHVPAAEYSLMERFH------------------PK
Roseiflexus_castenholzii/1-269             ------------DMRIIVGHGSGSFGHVYAQRYGIHRG-LAPNAD-------------WM
Herpetosiphon_aurantiacus/1-267            ------------NQPILLGHGGGSFGHYWAERYQTAQGIINEQS--------------WW
Desulfurococcus_kamchatkensis/1-262        -------------VKLILGNGGGSFAHYAVRKYCGDGR--------------------ID
Thermosphaera_aggregans/1-261              ------------EVSIVLGNGGGSFAHYAVLKYGSNDP--------------------TR
Staphylothermus_hellenicus/1-266           ------------KTRLLLGNGGGSFAHFVVEKYKMYDE--------------------KI
Hyperthermus_butylicus/1-260               -------------RLAGIVIGGESYGHYVAKTLGEAGVSAG------------------E
Aeropyrum_pernix/1-249                     ------------GRLAALVHGGGSFGHAAVAEARAQGGLTP------------------R
Ignicoccus_hospitalis/1-236                --------------ELVVVHGGGSFGHPKVKEIIESG----------------------H
Thermofilum_pendens/1-259                  ------------GYDIVLVHGGGSFGHPTAAKYRLHEGGLSPEKVR--------------
Methanocaldococcus_jannaschii/1-260        ----------NKEIKLILVHGGGAFGHPVAKKYLKIEDGKKIFINM------------EK
Methanococcus_maripaludis/1-257            ----------NEEFKLIIVHGGGSFGHPVAKKYLKDGKFVDMG----------------K
Metallosphaera_sedula/1-224                ---------------LVIVHGGGSFGHFEAGRNVP------------------------V
Sulfolobus_solfataricus/1-241              ------------DKKIILLHGGGSFGHYEASIFD------------------------DN
Archaeoglobus_profundus/1-239              --------------KLILVHGVGSFGHPFVVKYRLKEEKN------------------LE
Methanospirillum_hungatei/1-256            -------------IPLLLIHGAGSCGHPQARQYHIQSGVSREN---------------RE
Methanosarcina_mazei/1-260                 --------------KMIVVHGAGSFGHTYAKKYGLDRTFDP------------------E
Methanosaeta_thermophila/1-248             --------------NLIVVHGAGSFGHIHAKNFGLPERFSG------------------E
Nitrosopumilus_maritimus/1-247             --------------PIIIVHGGGSYGHYWSVKYDMHTKERKYDLR---------------
Cenarchaeum_symbiosum/1-246                --------------PVVVVHGGGSFGHYWSVKYDMHTKPARYEIR---------------
Ferroplasma_acidarmanus/1-280              --------------QMVIIHGGGSFGHIKSKEYGLPGQVSERT---------------ME
Picrophilus_torridus/1-242                 --------------DIIIVHGGGSFGHIKAKEYGLPGKISVKTLS---------------
Pyrococcus_furiosus/1-256                  -------------EKFIVVHGGGSFGHPLAQKFRIRDGLKDYSSR--------------H
Thermococcus_barophilus/1-266              -------------ENFILVHGGGSFGHPNAKEYKIPEGLVGNVDRK------------RI
Chloroflexus_aggregans/1-268               ------------TLAIVLGHGSGSFGHHYAARYGIHLG-TPIDAD-------------HT
Pseudomonas_syringae/1-261                 ------------SXPVILIFGGGSFGNVAPTEYRIFER-QHGLPS-------------AN
Shewanella_denitrificans/1-279             ------------ATPILLVLGGGAYGHPPVHEYGLASARGNGTV---------------S
Streptomyces_wedmorensis__fomA/1-266       ------------RGRMVLISGGGAFGHGAIRDHDSTHAFSLAGLT-------------EA

Anolis_caroliensis-lizard/1-281            GLCLTRLSVTKLNHLVIEHLVSCGI--PAVGIST--------------FG-TWKTASK-N
Callorhinchus_milii-shark/1-249            TILLYRN---REKSRTIANMP------VAFVLLA--------------FG-IWKTAGR-R
Saccoglossus_kowalevskii/1-283             GFCHTRISVTKLNHLVTSEFNSLGI--PAVGIST--------------CG-SWKTENK-H
Branchiostoma_floridae/1-298               GFCRTRQSVTKLLHIVTEEFVRLGI--PAVGVSP--------------LS-SWVTDDA-S
Strongylocentrotus_purpuratus-urchin/1-280 GFCKTRISVTKLNHMLVEALVNKGV--HATGISP--------------CG-RWKTRERGQ
Paracentrotus_lividus-sea_star/1-236       GFCKTRLSVTKLNHMLVEALVNKGV--HATGISP--------------CG-RWKTRGRGQ
Lottia_gigantea/1-283                      GFSLTRQSVLKLNASVIENLLKEGI--PAISLSP--------------FL-SWKTDNR-I
Nematostella-anemone/1-278                 GFVKTRLSVTKLLHKVCEVFVEQGI--PAVALSP--------------CG-SWKTSGG-H
Acropora_palmata/1-176                     ---MHCGAWSRLQQMVVESFISNGI--PAVGISP--------------CG-SWMTTEG-V
Montastraea_faveolata/1-203                ---MYRGPWRGLQHTITAVFLENGI--PALGISP--------------CG-SWLTSSG-V
Trichoplax_adhaerens/1-281                 GFSQTRLSVTKLNHIIVQALIEKDV--PAVSISP--------------CG-LWKTTDR-S
Rhizopus_oryzae/1-279                      GFSHIRACLQQLNTTIITQLEQRGV--PVLNITP--------------LD-YLHARHGQD
Mucor_circinelloides/1-306                 GYSHIRNCLQALCQAIASKLEARHV--PALTMSP--------------ID-YVETANCED
Epichloe_festucae/1-297                    GMAFTRQRVLQLHHHVLQRLQDRGR-LPVLSVST--------------YD-TVETDRGEL
Spizellomyces_punctatus/1-299              GAAKTRHAVATLNRMVVGELIDRGI--PAVSVNP------GSLLDGNWSR-GEGTKPD-P
Phaeodactylum_tricornutum/1-361            GLATTRLSVLHLNHYVVSSLLANGI--NAIGISP-----------CFSNRHMEADGRD-T
Thalassiosira_pseudonana/1-355             GLSKTRQSVQKLNTAVVNSLIANGV--NAVGISPGMSTPLLRAHGGTSLPNDGDDDND-S
Perkinsus_marinus/1-320                    GFVATRASVGRLNSMVVDVLVNHGL--KAVGVGS--------------CGYLWTSDKK-L
Polysphondylium_pallidum/1-283             GMIATRFSVLKLLNLVCLECQKRSI--SAMPCSP-------------FDG--WMTDNN-V
Dictyostelium_discoideum/1-292             GILRTRESVTTLLDIVKKELVNNGL--NVCSMSP-------------FSS--WRTNNG-G
Arabidopsis_thaliana/1-332                 GFVATRISVTNLNLEIVRALAREGI--PTIGMSP------------FSCG--WSTSKR-D
Rice/1-335                                 GFVATRISVTSLNQEIVRALAREGI--PSVGMSP------------FACG--WSTKQR-N
Maize/1-340                                GFVATRISVTSLNQEIVRALAREGI--PSVGMSP------------FACG--WSTQQR-K
Poplar/1-335                               GFVATRISVTTLNLEIVRALAREGI--PTIGMSP------------FSCG--WTTPER-N
Ricinus_communis/1-337                     GFVATRISVTTLNLEVVRILARDGI--PSVGMSP------------FSCG--WSTSQR-N
Vitis_vinifera/1-340                       GFVATRISVTTLNLEIVRALAREGI--PSIGMSP------------FSCG--WLTSER-N
Soybean/1-335                              GFVATRISVTTLNLEIVRALAREGI--PSIGMSP------------FSCG--WFTSER-H
Selaginella_moellendorffii/1-312           GFVATRLSVTKLNHKVIRALASQGI--PAVGMPP------------FAAG--WSTHKR-I
Physcomitrella_patens.2/1-351              GFVATRISVTKLNQGVVRSLALEGI--PAVGISP------------FSAG--WSTQNK-A
Chlamydomonas_reinhardtii/1-344            GFTLSRASVTKLNGLVVGALVAAGV--PAVGLSP--------------LGLYHTCNKQ-V
Volvox_carteri/1-321                       GFTLTRSSVTRLNGLVTGELVAAGI--TAVGLSP--------------LGLYTTCGRQ-V
Micromonas_pusilla.CCMP1545/1-411          GLAKTRLSVTKLNHIVTSKLVEKGI--AAVGMSP--------------FG-AWSTSGK-T
Micromonas_pusilla.RCC299/1-402            GVDKTQASVRKLCRLVCDELTSHTAGSRAWVKPA----------PISPYG-KFFTVGK-K
Ostreococcus_lucimarinus/1-352             GVDGTRAKVLELNRLVVDALRDAGV--NARGMTP------------WREG--WRTRGP-G
Ostreococcus_tauri/1-356                   GVEATRAAVRTLNGIVVDALRENGV--RAVGVTP------------SEVG--WHTRGR-G
Entamoeba_hisolytica/1-259                 GVIECEMAMQELSSIIVNSLIKEGI--SAIPFHP-------------FNF--VVTENK-R
Entamoeba_invadens/1-257                   GVLLCEMAMQKLNEIVINSLEQESL--HVFPFHP-------------VDF--IVTTKR-R
Roseiflexus_castenholzii/1-269             GFALTSAAALRLNRIVVDELLAAGV--PALALQP--------------ST-TLAARGG-Q
Herpetosiphon_aurantiacus/1-267            GVARVADAMARLNRAVVGACLDADL--PAIGIQP--------------MA-SSLANAG-E
Desulfurococcus_kamchatkensis/1-262        CVVKCHQATRLLNRIITDHLVEAGIPVSSIQTSA---------------V-ITVSQDG-E
Thermosphaera_aggregans/1-261              LLVKCQQATRLLNRLIVDYLVFNNI--PATSLQT--------------SA-IIGYGKD-S
Staphylothermus_hellenicus/1-266           LLVNCHRATRLLNSIIVDYLLDHGL--LVSTMQT--------------SA-IIYGGKR-G
Hyperthermus_butylicus/1-260               ALSAIPRAMMELSLLAADVFSMYGL--HVVVYPP---------------H-SFCKPQG-L
Aeropyrum_pernix/1-249                     AAPRVQLAMLKLAMEVAASLLKYGV---EVSIHP--------------PH-TFCSGGE-C
Ignicoccus_hospitalis/1-236                TLADKGFEVIRVMNLMTNLVVEALG----QPFAP------------------YSTPSL-W
Thermofilum_pendens/1-259                  GFSETRYWMTKLNTLVVEHLLRLNV--PAVSLQT--------------SA-IAVNSGG-K
Methanocaldococcus_jannaschii/1-260        GFWEIQRAMRRFNNIIIDTLQSYDI--PAVSIQP--------------SS-FVVFGDK-L
Methanococcus_maripaludis/1-257            GYWEIQKAMRKFNNIVIEELQNFEI--PAVSIQA--------------SS-FITFDNE-S
Metallosphaera_sedula/1-224                RVSLTSASMEELNTILIREMAVRGI--KGFPLPG--------------------------
Sulfolobus_solfataricus/1-241              RIVRTSEAMQELNYIVAKHLLKSGI--KAISVPG--------------------------
Archaeoglobus_profundus/1-239              GVVRAHMSCKELNAMICEAMLMYGL--KPFPVHP---------------L-LTFKLRG-G
Methanospirillum_hungatei/1-256            GIYATHQAVSALNELVVRTLRAEGI--EAVSVHP--------------LE-GMVASGG-E
Methanosarcina_mazei/1-260                 GAIVTHESVKKLASKVVGALNSFGV--RAIAVHP--------------MD-CAVCRNG-R
Methanosaeta_thermophila/1-248             GLLKTHLSVSDLNRIVVEALHDAGV--DALPLHP--------------LS-SVVLRDG-R
Nitrosopumilus_maritimus/1-247             GVSIVKNSMIELNKIILDSFLKNKL--KPYCLPP---------------T-DFMTGNK-P
Cenarchaeum_symbiosum/1-246                GVATVKNSMARLNMMVLDSLLNAGL--SPYSVPP-----------------ACISRSG-R
Ferroplasma_acidarmanus/1-280              GMNIVHNDMAELDLKISKIFQENKIYNISLPVSS------------------LVYNNK--
Picrophilus_torridus/1-242                 GMNVVHNDMLELNVKVSRILNENGIFNISIPIPA------------------ISRNGR--
Pyrococcus_furiosus/1-256                  GFVVTHLAMVDLASRIAKCFLEQHV--PGFPISS--------------SS-VFITSRG-K
Thermococcus_barophilus/1-266              GFSKTHQAMLRLNDLIVQTFLEKGL--PAYSVSS--------------SS-IFLIENK-E
Chloroflexus_aggregans/1-268               GFALTAAAALRLNRIVVDALLTAGV--PAVSLQP--------------SA-SLSSAHG-Q
Pseudomonas_syringae/1-261                 LPMMMTSIMFSMLSDITRIFVEQGL--RVYPFQS--------------SA-LLGVDEE-G
Shewanella_denitrificans/1-279             NLQFSR--LTTGLYKLMVDFMQISYE-NSLEMHP-------------FQS-SSLFMCQ-D
Streptomyces_wedmorensis__fomA/1-266       TFEVKKRWAEKLRGIGVDAFPLQLA--AMCTLR-------------------NGIPQL-R

Anolis_caroliensis-lizard/1-281            VTQDGI-----DAVKEALDAGYVPVLHGDCALDSE-QHCCVLSGDTIIEVLAKKFSPR--
Callorhinchus_milii-shark/1-249            VIQHNI-----SSVHDVLAAGYVPVLHGDCVLDSN-QHCCILSGDTIIEXXXX-------
Saccoglossus_kowalevskii/1-283             VIKSDI-----ESVLSLLDGGFLPILHGDCVIDSK-LGCTILSGDTIIETLCQKCNPK--
Branchiostoma_floridae/1-298               VVKADT-----DNIRNMLLEGFLPVMHGDAVLDQK-RGCTILSGDTIIKHLCGVFRPP--
Strongylocentrotus_purpuratus-urchin/1-280 VVSSDC-----EGVNELLQAGFLPVLHGDCVLDEE-LGCTILSGDTIMQVLCEFFKPK--
Paracentrotus_lividus-sea_star/1-236       VVSNDC-----DSVNELLQAGFLPVLHGDCVLDEE-LGCTILSGDVIIEVLCEFFKPK--
Lottia_gigantea/1-283                      VQEDGCE-----MIHAVLNQGYMPVLHGDAVFDKS-LGCTILSGDTIIERICEKFEVK--
Nematostella-anemone/1-278                 VTKSAVN-----QMIDLLEAGFVPVIHGDCVLDNQ-IGCFILSGDKIIEQIVKEQRPN--
Acropora_palmata/1-176                     VTRSAIH-----PIVQCVAAGFVPILHGDCVLDTK-QGCAALSGDKIIEKLVEELHPS--
Montastraea_faveolata/1-203                VTRSAVT-----PIVELLEAGFVPILHGDCVLDDV-QGCSILSGDKIIQRLAEELRPK--
Trichoplax_adhaerens/1-281                 VTSTFLQ-----PINDLLRAGFVPVVHGDAVIDTS-LGCTILSGDTIIQILAENLCPK--
Rhizopus_oryzae/1-279                      TPTERFE-ALVERTSQYLQLGFVPVLHGDAVLDDM-RGCTILSGDVILYHLSKWLPVA--
Mucor_circinelloides/1-306                 TPTSAFE-AMADRVKRYLALGFVPVLHGDAVLDQI-RGCTILSGDIIMYQLTRLLPQVR-
Epichloe_festucae/1-297                    TPESSIR--LVTRVQKLLAQGFVPVLFGDAVLDSA-LGTTILSGDALMHKLATELPEVQ-
Spizellomyces_punctatus/1-299              PPQTAYT-ALLRHVTALIDAGYIPVLHGDVVSSPL-FGSMILSGDDLIVAFAYALKPRR-
Phaeodactylum_tricornutum/1-361            VAQEELI----QSVEASLKAGLVPVLHGDACLYGR-DGAGILSGDTVVEILAKAPWIS--
Thalassiosira_pseudonana/1-355             VEGMKLL---CQSIHQSLQAGLVPIVHGDACLLYDSIRAGILGGDTLAEGIATLWDESVG
Perkinsus_marinus/1-320                    PWFTMCGQVLYDGLSGLLNSGIIPVMHGDCVLDEK-QVCTILSGDTIFYWMCRAFKPS--
Polysphondylium_pallidum/1-283             VIKHNAD-----SIQSMLDLSIIPVLHGDVCLDRS-KGCTILSGDTIIQVLCEQLKPQPT
Dictyostelium_discoideum/1-292             DNVVQDNI-DNINFSLNHFPKIIPILHGDVCLDNT-LGCTIISGDTIIRELCYKLKPN--
Arabidopsis_thaliana/1-332                 VASADLA-----TVAKTIDSGFVPVLHGDAVLDNI-LGCTILSGDVIIRHLADHLKPE--
Rice/1-335                                 LESVDAS-----QIMLSLHVGFVPVLHGDAVLDEL-LDCTILSGDVIIRHLAQLLSPK--
Maize/1-340                                LASANAS-----QIFQSLHAGFVPVLHGDAVLDEL-LDCTILSGDVIIRHLAQLLSPK--
Poplar/1-335                               MASADLS-----MVAQAINSGFVPVLHGDAVLDDL-QGCTILSGDVIIRHLAAYLKPE--
Ricinus_communis/1-337                     MESADLS-----MVAKAIGSGFVPVLHGDAVLDEF-QGCTILSGDVIIRHLAAYLKPE--
Vitis_vinifera/1-340                       VASADVS-----MVAKAIDSGFVPVLHGDAVLDEA-QDCTILSGDVIIRHLAAQLKPE--
Soybean/1-335                              ISSADLS-----SVAKAIDSGFTPVLHGDAVLDEI-QGCTILSGDAIISHLAAYSKPI--
Selaginella_moellendorffii/1-312           VDCDNVS-----GVREAVDAGFVPVLHGDAVRDSH-QGCCILSGDVIVRRLAEELQPS--
Physcomitrella_patens.2/1-351              LKRDNVL-----EVQRAVDAGFVPVVHGDAVLDST-LGCTILSGDVLVSRLAQVIKPN--
Chlamydomonas_reinhardtii/1-344            AVSGGAS------ASALLAAGLLPVLHGDCVLDTA-LGCTVLSGDTLVRDLAERLRPQ--
Volvox_carteri/1-321                       HNSGAAA------VADCLRAGLVPVLHGDAVLDEQ-LGCTILSGDTLVRDLAERLRPQ--
Micromonas_pusilla.CCMP1545/1-411          MISAGIK-----AVNEALAAGLVPVVHGDAVLDEQ-QGCCILSGDTIMEELCGYVKCD--
Micromonas_pusilla.RCC299/1-402            LNRNLSR-GGFDEVRAALMEGKIPVLHGDVVNDAE-QGCAILSGDTLVECLTEEFKPK--
Ostreococcus_lucimarinus/1-352             RPETSGRD-EGRAVLDAVRDGVVPVIHGDVVEDVA-QGTSVLSADTIVEMCAKWAMEEWP
Ostreococcus_tauri/1-356                   AEAEASTS-GRGGVFDLLRDGAVPVIHGDVVIDDV-QGTSVLSGDDIVAWCARWAIEDGF
Entamoeba_hisolytica/1-259                 IVDMYLQ-----PLQMLVNQGVIPVVHGDVAMDIM-QGSCILSADQLVPELAIRFGCH--
Entamoeba_invadens/1-257                   ISECYLR-----PLELMLSQGIIPVLHGDVVTDTE-QGSCILSADQIVPFLSKKFKTH--
Roseiflexus_castenholzii/1-269             LTRWNTD-----ALERALHHRLVPVIHGDVAFDDI-QGSAIISTEQLLAHLAALPALQPT
Herpetosiphon_aurantiacus/1-267            IQQIGSQ-----PLATLLAAGTIPVIYGDVLLDVA-QGCTIASTERIFSALVGPLQPT--
Desulfurococcus_kamchatkensis/1-262        FKVFSEP------VYNFISAGLIPLLYGECIPWGN--GYHVVSTEKVFELLAESIKPA--
Thermosphaera_aggregans/1-261              LRAFTPP------LLNLVGNGIIPVVYGECILNEK-GLVEVFSTEKVFEILSSILKPS--
Staphylothermus_hellenicus/1-266           FVSFIK------PLKYLLRNNIIPSIYGECIIDEN-QAVRIVSTEEVFSILAEHIKVS--
Hyperthermus_butylicus/1-260               RPSCNWS-----LVMGYEWGRPIPLVYGDAYPCSS--GACIVSGDELAMEMACALGAS--
Aeropyrum_pernix/1-249                     VLET---------LKRDYRLGLTPMTFGDAVPADG--GVEIVSGDDLALWLAVELGVE--
Ignicoccus_hospitalis/1-236                DGRLNVR-----PLALAAKAGWVPVVQGNVVPPGR-----VLSGDEIVVELVKELGAE--
Thermofilum_pendens/1-259                  LSRISLD-----VLREMLARRLVPVLYGDAVIDLS-RGFSILSGDTLAARIAVELGAK--
Methanocaldococcus_jannaschii/1-260        IFDTSA-------IKEMLKRNLVPVIHGDIVIDDK-NGYRIISGDDIVPYLANELKAD--
Methanococcus_maripaludis/1-257            NLHFDTN-----AVEKMLDKGLIPVIHGDIVIDEKTDNFKIFSGDHALPFLSKKLNPD--
Metallosphaera_sedula/1-224                -RFFDLE-----RLERILDHGMVPVVFGDIKEDGT-----IISGDDLTISIAREYSLT--
Sulfolobus_solfataricus/1-241              -KFYTFD-----AVLSALEKDLVPLIYGDVKFDGS-----IISADDMSIDIAKRLNA---
Archaeoglobus_profundus/1-239              KITFDID-----IFEKALEEGFIPVTHGDMVYDVEDRFFKVLSGDDITLKLAKAFKAE--
Methanospirillum_hungatei/1-256            LSGYCLT-----HLHLMIDLGIVPVLHGDVVMDTE-KGACIVSGDQLVRVLAQKLGMK--
Methanosarcina_mazei/1-260                 IETMYLD-----SIKLMLEKGLVPVLHGDVAMDIE-LGTCILSGDQIVPYLAKELGIS--
Methanosaeta_thermophila/1-248             IHHMSTE-----VITEMLRRDVVPVLHGDVAMDLS-KGAGIVSGDQLVSYMARTLGAG--
Nitrosopumilus_maritimus/1-247             IPKKVKE------IEKISKSGLIPVTFGDALWYGQ-NKTFILSGDKIMTHLAKILKPK--
Cenarchaeum_symbiosum/1-246                PLSAGIK-----ETGEEARAGLVPVTYGDALWAGR-GRTYILSGDRIMGMLARALRPR--
Ferroplasma_acidarmanus/1-280              -KNYNI-------FSKYLELGITPISYGDTYIHSN--EIGIYSGDNIAYDISKILHPE--
Picrophilus_torridus/1-242                 IDYTS--------FIEYIKAGITPVSFGDIYVKNG--TIGIYSGDNIVYDLSFIYKPD--
Pyrococcus_furiosus/1-256                  IVSGYLN-----SVEEAIRREFVPILFGDVSFDVE-KGIEIVSGDEIMVYLAKHFKPE--
Thermococcus_barophilus/1-266              IVYAELE-----ILRKLLELKFIPILFGDTAIALD-KGIGILSGDQIVSYLAKMLKPN--
Chloroflexus_aggregans/1-268               ITHWEIG-----PISAALQRRLVPVIHGDVAFDTV-QGTAIISTEALLRFLALHSPLRPR
Pseudomonas_syringae/1-261                 RVTLHAR-----QLATAMASGYMPLLTGDLLLRGE-QEAQVFSSDNIAPLLAADFEVR--
Shewanella_denitrificans/1-279             GKVKSVF---SEAIEKSLQFNDIPLLTGGSAYDTT-LGQLVFGSDRIPELLTKMFKVS--
Streptomyces_wedmorensis__fomA/1-266       SE----------VLRDVLDHGALPVLAGDALFDEH-GKLWAFSSDRVPEVLLPMVEGRL-

Anolis_caroliensis-lizard/1-281            -----------RVVFLTDVNGIFSCPP-----------------------DTPGAKLLDH
Callorhinchus_milii-shark/1-249            ---------------------------------------------------XXGSQLLSQ
Saccoglossus_kowalevskii/1-283             -----------RVIFLTDVEGIYDKPP-----------------------VNDDAKLLPL
Branchiostoma_floridae/1-298               -----------RVVFLTDVPGIYDRPP-----------------------EQPGAQLIPE
Strongylocentrotus_purpuratus-urchin/1-280 -----------RVVFLSDVQGIFTKPP-----------------------LNPKAKLIPR
Paracentrotus_lividus-sea_star/1-236       -----------RVIFLSDVEGVFTKPP-----------------------THPDSKLIPR
Lottia_gigantea/1-283                      -----------KVVFISDVDGIFDKPP-----------------------NNLEAKLIKH
Nematostella-anemone/1-278                 -----------RVVFLTNVDGVFDRPP-----------------------EKQGAKLLDH
Acropora_palmata/1-176                     -----------RVVFLTDVGGIYNKPP-----------------------DNEDATLIRT
Montastraea_faveolata/1-203                -----------RVVFLTDVDGIYDKPP-----------------------EKEDSVLLRK
Trichoplax_adhaerens/1-281                 -----------RIIFITDTNGIYDRPP-----------------------HNDDAKLLRY
Rhizopus_oryzae/1-279                      -----------RCVFLTDVEGIYKADPK-------------------LKLVPQSFEILSH
Mucor_circinelloides/1-306                 -----------RCVFITDVCGIYKLDPK--------------------LHPEEENELIRH
Epichloe_festucae/1-297                    -----------RCVFVTDVAGIYTRDPK----------------------RFQDATLIYQ
Spizellomyces_punctatus/1-299              -----------GCVFLTDVDGIFDRDPK----------------------VFANAELLRT
Phaeodactylum_tricornutum/1-361            -----------RVLFLTDVDGVFDKDP----------------------RMYSDANLLRS
Thalassiosira_pseudonana/1-355             NRKNSRGDKISRVIFITDVAGVFSADP----------------------KADPNAVLVRS
Perkinsus_marinus/1-320                    -----------RGIFLTDVAGIYDKPP-----------------------NEEGAKLIPR
Polysphondylium_pallidum/1-283             -----------RAIFITDVSGVYDRPP-----------------------NEPNAIIISN
Dictyostelium_discoideum/1-292             -----------KCIYVSDVNGVYDSNPK----------------------ENENAQLLSN
Arabidopsis_thaliana/1-332                 -----------YVVFLTDVLGVYDRPP---------------------SPSEPDAVLLKE
Rice/1-335                                 -----------YVVFLTDVHGVYDRPP-----------------------SDPNAVLLRE
Maize/1-340                                -----------YVVFLTDVHGVYDRPP-----------------------TDSNAVLLRE
Poplar/1-335                               -----------YVVFLTDVLGVYDRPP-----------------------SEPNAVLLRE
Ricinus_communis/1-337                     -----------SVVFLTDVLGVYDRPP-----------------------SEPGAVLLRE
Vitis_vinifera/1-340                       -----------YVVFLTDVLGVYDRPP-----------------------TEPNAVLLKE
Soybean/1-335                              -----------YVVFLTDVYGVYDRPP-----------------------TEPNAILLKE
Selaginella_moellendorffii/1-312           -----------YVVFLTNVPGVFDRPP-----------------------SEENAVLLQE
Physcomitrella_patens.2/1-351              -----------FVVFLTNVPGVFDRSP-----------------------EQPGATLLRE
Chlamydomonas_reinhardtii/1-344            -----------YVVFLTNVPGVYDRPP-----------------------EEAGARLLRR
Volvox_carteri/1-321                       -----------YVVFLTNVTGVYDRPP-----------------------EEEGARLLRR
Micromonas_pusilla.CCMP1545/1-411          -----------RVVFLTNTLGVFDRPPEDHYKLWRGEGSRPPLDFPNEEWEEATKLLIEI
Micromonas_pusilla.RCC299/1-402            -----------RVVFVSDVEGIFTAKPLHKNGPCDLCPD----------GKTPPPALLRE
Ostreococcus_lucimarinus/1-352             DECP-------RVVFCSDVFGVYDSPPTTRIINADVTDGDV----PLRVNETETAVLLRD
Ostreococcus_tauri/1-356                   AARA-------RVVFLSDVWGVYASPPRCAPITNPSDTS------ALTIAPGEDAVLLRE
Entamoeba_hisolytica/1-259                 -----------R-------IGFICNSP----------------------VLNDKREVIPL
Entamoeba_invadens/1-257                   -----------R-------VGFISQSP----------------------VYDNKKEVIPL
Roseiflexus_castenholzii/1-269             -----------RIVLVGES-GVYTADP----------------------RNNPNAERIAR
Herpetosiphon_aurantiacus/1-267            -----------QIILLGEQA-VYDADP----------------------RQHADAQPIPL
Desulfurococcus_kamchatkensis/1-262        -----------RIVLLTDVKGVYTCNP----------------------SRCSDPVLIKS
Thermosphaera_aggregans/1-261              -----------RILLLTDVEGVYSCNP----------------------KKCSNPGLIKR
Staphylothermus_hellenicus/1-266           -----------RIVLLTDVEGVFTCDP----------------------KRCEDAELIPR
Hyperthermus_butylicus/1-260               -----------GVIYATTVPGVLGRDG----------------------------RPIPR
Aeropyrum_pernix/1-249                     -----------CLIYATRVPGVVKGGR--------------------------VVPVIRG
Ignicoccus_hospitalis/1-236                -----------RAGVATDVDGVYETWP-------------------------PKGGPLKE
Thermofilum_pendens/1-259                  -----------SLVYVMGAGGVYSKPP-----------------------GSPDARLLRE
Methanocaldococcus_jannaschii/1-260        -----------LILYATDVDGVLIDNK-----------------------------PIKR
Methanococcus_maripaludis/1-257            -----------LSLHASDVDGVWDLNF----------------------------KIIEN
Metallosphaera_sedula/1-224                ------------ALFATDVDGILVNGQ-----------------------------VIPE
Sulfolobus_solfataricus/1-241              -----------RLLFAIDKAGIIGRGG----------------------------GVISE
Archaeoglobus_profundus/1-239              -----------KIGFATDVEGVYVDGK-----------------------------LADV
Methanospirillum_hungatei/1-256            -----------RIGLATDVPGLLDADG----------------------------SVVRE
Methanosarcina_mazei/1-260                 -----------RLGLGSAEDGVLDMEG----------------------------KPVPE
Methanosaeta_thermophila/1-248             -----------MVAMGTDVDGVMIDGR-----------------------------VLSC
Nitrosopumilus_maritimus/1-247             -----------LCIFALNEDGVYSDLK--------------------------SKKLIHE
Cenarchaeum_symbiosum/1-246                -----------LCIFAMNVDGLYESPR--------------------------TRKLIPE
Ferroplasma_acidarmanus/1-280              -----------FVIFFSDVDGIFDKNP----------------------KNNPDAKLLKT
Picrophilus_torridus/1-242                 -----------TVVFMSNVDGIFDKNP----------------------------EIYKD
Pyrococcus_furiosus/1-256                  -----------KVIFLMDVDGLYTKFP--------------------------GGELIRE
Thermococcus_barophilus/1-266              -----------KVIFLMDVDGIYNKNP-----------------------RERDAKLIEE
Chloroflexus_aggregans/1-268               -----------RIILVGEA-AVYTADP----------------------HRDPTAQPIPL
Pseudomonas_syringae/1-261                 -----------RVLYYSDVAGVYDQ----------------------------GNALVPW
Shewanella_denitrificans/1-279             -----------KCIFVSDVDGVYEHT---------------------------GGKMFDE
Streptomyces_wedmorensis__fomA/1-266       -----------RVVTLTDVDGIVTDGA-------------------------GGDTILPE

Anolis_caroliensis-lizard/1-281            IIIHPNGT------------------------MEP------------------------H
Callorhinchus_milii-shark/1-249            VSFRADGS------------------------LAL------------------------P
Saccoglossus_kowalevskii/1-283             INISKDGG------------------------FTA------------------------P
Branchiostoma_floridae/1-298               IQVDRDRK------------------------VHV------------------------S
Strongylocentrotus_purpuratus-urchin/1-280 IQVKQDGS------------------------IAT------------------------I
Paracentrotus_lividus-sea_star/1-236       IQVKQDGG------------------------IAT------------------------N
Lottia_gigantea/1-283                      IQVRTDGD------------------------IFM------------------------D
Nematostella-anemone/1-278                 IGILETGE------------------------VNA------------------------S
Acropora_palmata/1-176                     VFVNPSGK------------------------------------------------MSVA
Montastraea_faveolata/1-203                VYVKSDGQ------------------------------------------------MNVT
Trichoplax_adhaerens/1-281                 ISVTKDGK------------------------VTN------------------------E
Rhizopus_oryzae/1-279                      ISVKDTME----------------------------------------------------
Mucor_circinelloides/1-306                 IKVNAATEDDHP----------------------------------------QPDQLQQQ
Epichloe_festucae/1-297                    LRCSSEQG-------------------------------------------------IDE
Spizellomyces_punctatus/1-299              IHVNRKTG------------------------------------------SSNCNCVTSE
Phaeodactylum_tricornutum/1-361            IQVDAKTG------------------------SIVG--------------------VKVD
Thalassiosira_pseudonana/1-355             LKVDRNTG-----------------------EVMIDKSNDNSD------GNEGERSATLS
Perkinsus_marinus/1-320                    ISARGDAK------------------------S--------------------------N
Polysphondylium_pallidum/1-283             ISSSS---------------------------LTN---------------------NDIS
Dictyostelium_discoideum/1-292             IKVSEIDG------------------------CDNNN--------------NNNNNDNIK
Arabidopsis_thaliana/1-332                 IAVGEDGS------------------------WKVVNP--------------LLEHTDKK
Rice/1-335                                 IAVDENGS------------------------WSIVKP-------------AL-KGNKKG
Maize/1-340                                IEVDDIGG------------------------WSIVKP-------------ALLQGNTKG
Poplar/1-335                               IAVSEDGS------------------------WSVVKP--------------TLEDMKKQ
Ricinus_communis/1-337                     IAVNEDGS------------------------WSVVNP--------------TRQNMNNQ
Vitis_vinifera/1-340                       IAVSEDGS------------------------WSVVKP--------------TLKEMNKQ
Soybean/1-335                              IAVAEDGS------------------------WSVVKP-----------------KLQNS
Selaginella_moellendorffii/1-312           IVVYEDGT------------------------WSIARP-----------------RLEAP
Physcomitrella_patens.2/1-351              IVVYEDSS------------------------WTIVDP-------------PLGIGSNGG
Chlamydomonas_reinhardtii/1-344            IMVTPDGG------------------------WRVAEV-------EGGSDGGDSGGGDSS
Volvox_carteri/1-321                       IVVRKDGS------------------------WRVAEA---------------DGGGEVD
Micromonas_pusilla.CCMP1545/1-411          VTFKRNHQNSISWVVRNT-------GSSLTGLFDDSQEMEGSY--QRIIGEGEKGISMAE
Micromonas_pusilla.RCC299/1-402            IQVNPDGSWIATRAFGPCGYLYLVEKSEWQREWQQANPLWKFSDATDERVVESAVGTTDA
Ostreococcus_lucimarinus/1-352             IIVDADADASDG-TRV---------------PWRCARASPLAR----LAEAADAAALDAT
Ostreococcus_tauri/1-356                   IVVDGDSD--DGDASP---------------AWRCVRAAPLV---DPRRDAPLDAVPSAT
Entamoeba_hisolytica/1-259                 INEQNYES------------------------IKT-------------------------
Entamoeba_invadens/1-257                   INLSNYNT------------------------IKK-------------------------
Roseiflexus_castenholzii/1-269             IDHHNVAD------------------------VLA-------------------------
Herpetosiphon_aurantiacus/1-267            INRTNYAT------------------------IIA-------------------------
Desulfurococcus_kamchatkensis/1-262        INKNNISQ------------------------VLE-------------------------
Thermosphaera_aggregans/1-261              IDNDNLGA------------------------VLE-------------------------
Staphylothermus_hellenicus/1-266           IDRNNIDT------------------------VLS-------------------------
Hyperthermus_butylicus/1-260               LRLSELDR---------------------------------------------------V
Aeropyrum_pernix/1-249                     LGEFED--------------------------LG--------------------------
Ignicoccus_hospitalis/1-236                ASPCDVEAK---------------------------------------------------
Thermofilum_pendens/1-259                  IAENDVLT------------------------VGG-------------------------
Methanocaldococcus_jannaschii/1-260        IDKNNIYK------------------------ILN-------------------------
Methanococcus_maripaludis/1-257            INSKNIED------------------------VLK-------------------------
Metallosphaera_sedula/1-224                LNEPHS--------------------------LTN-------------------------
Sulfolobus_solfataricus/1-241              LRGIDEVS------------------------ILM-------------------------
Archaeoglobus_profundus/1-239              VTWKDLDK------------------------IGF-------------------------
Methanospirillum_hungatei/1-256            LRRTMAHT------------------------IRI-------------------------
Methanosarcina_mazei/1-260                 ITPETFEEFR-----H---------------CIGG-------------------------
Methanosaeta_thermophila/1-248             ITPNDMHS------------------------LES-------------------------
Nitrosopumilus_maritimus/1-247             LKGERPS-------------------------IS--------------------------
Cenarchaeum_symbiosum/1-246                LGRGTPE-------------------------LG--------------------------
Ferroplasma_acidarmanus/1-280              ISTDFQYD----------------------------------------------------
Picrophilus_torridus/1-242                 ARLLRNPD------------------------IEL-------------------------
Pyrococcus_furiosus/1-256                  ISASELKE------------------------LLT-------------------------
Thermococcus_barophilus/1-266              LNAEEIRH------------------------LLE-------------------------
Chloroflexus_aggregans/1-268               IDRTNIAQ------------------------VLH-------------------------
Pseudomonas_syringae/1-261                 VGNANAAC------------------------MEA-------------------------
Shewanella_denitrificans/1-279             ITPELYPS------------------------LSD-------------------------
Streptomyces_wedmorensis__fomA/1-266       VDARSPEQ------------------------AYA-------------------------

Anolis_caroliensis-lizard/1-281            ILTSVLPHDTTGGISMKLQASIHIVS-QSRGDVPVLICKLDSD-------AAERACLTGE
Callorhinchus_milii-shark/1-249            IQTASLPHDTTGGISNKLRTAVNILL-NSKGATRVFICDIDSE-------QVLLQ---GV
Saccoglossus_kowalevskii/1-283             IATSSNMHDTTGGIAFKLKNIISIIT-RSEGKIPVFICKIDSE-------SAKHACFHGN
Branchiostoma_floridae/1-298               IATSSQAHDVTGGIALKLKSAVDIVT-ESNGHTCVMVCGIQSQ-------AAVRACVEGQ
Strongylocentrotus_purpuratus-urchin/1-280 IATEQLDHDVTGGIKTKIAAACTIVS-QSGGAIPVFVCKLGGH-------SAELACWHSD
Paracentrotus_lividus-sea_star/1-236       IATEELDHDVTGGIKGKILGVI--------------------------------------
Lottia_gigantea/1-283                      IQTSQSVNDVTGGIKLKLQAAINIVK-ASP-KTTVYISKVSSN-------STQDICLNDN
Nematostella-anemone/1-278                 IGTSLTFHDVTGGIMGKIQTAVNIIK-STKGQTGVFVTKIGSQ-------GSHDACINGS
Acropora_palmata/1-176                     IATSVLTHDVTGGVCEKLRTASNIVLISGGKTRVFVANVMAEAN------VYS-------
Montastraea_faveolata/1-203                IATSNLHHDVTGGIREKLQTASNIIRISEQHSRVFVLNIMSET-------VAYSVCSRGV
Trichoplax_adhaerens/1-281                 IETSQLEHDVTGGVQTKIASAAHIVS-KCN--IPVHVVKLGSA----AAWKLL---DKGE
Rhizopus_oryzae/1-279                      VSTSFTVADVTGGIQGKIEWAKRMVS-DCQVDV--MICRW--------------------
Mucor_circinelloides/1-306                 QQNRMAVADVTGGMQGKVKWAKRMVS-ESQQDLDTVICRW--------------------
Epichloe_festucae/1-297                    SDASAGVDDVTGAMSSKWQWTKRIMA-DAPHIRQVVICQASDLD------KAL--SVTGE
Spizellomyces_punctatus/1-299              SITVGSAVDVTGGMSKKLASAIAVVR-GPLEDVQWNCRVVLVKAGSDDASKAL----AGD
Phaeodactylum_tricornutum/1-361            ASGSSHEQDTTGGLKTKLASAVAVVN-LGLNVTIARCGSTSAQ-------QAI---QSIA
Thalassiosira_pseudonana/1-355             VGESSHAHDVTGGLKVRRCTMYIARD-IYVDEALTHKLVWYTK-----------------
Perkinsus_marinus/1-320                    IKTCVPAHDVTGGIETKLASAVEVAKDLGIPVYIVQ------------------------
Polysphondylium_pallidum/1-283             VSNSKSEHDVTGGMRAKLQSALNIAN-MNIDVVIIGGDSKSIL-------ESP----NLL
Dictyostelium_discoideum/1-292             LTLDSNSKDVTGGMKAKLDSAIKVAR-NKTYTLIIGGSYSKN--------EIL----DTI
Arabidopsis_thaliana/1-332                 VDYSVAAHDTTGGMETKISEAAMIAK-LGVDVYIVKAATTHSQ-------RAL----NGD
Rice/1-335                                 VEISVAAHDTTGGMETKILEAAAIAR-LGVDVYITKVGTEHSL-------RAL----KGD
Maize/1-340                                VEISVAAHDTTGGMETKILEAAVIAR-LGIDVYITKAGTEHSL-------RAL----KGD
Poplar/1-335                               VETTVAAHDTTGGMATKISEAALIAK-LGIDVYIVKAATTHSS-------RALSGEVRGA
Ricinus_communis/1-337                     VEITVAAHDTTGGMETKISEAAMIAK-LGIDVYIVKAATSHSL-------KALSGELRGT
Vitis_vinifera/1-340                       VEITVAAHDTTGGMVTKIWEAAMIAK-LGIDVYIVKAATDDSL-------RALRGELKGN
Soybean/1-335                              IELTVAAHDTTGGMKTKIAEAAMIAK-LGIDVYIVKAATSHSL-------RALNGDLRSS
Selaginella_moellendorffii/1-312           VKTEMASHDTTGGMATKIAEAASISR-LGMDVYIVEAGTEHAL-------QAL----KGN
Physcomitrella_patens.2/1-351              VETAVAAHDTTGGMSTKIAEAASIAA-MGIDVFIVEAGTPHAL-------EAL----RGK
Chlamydomonas_reinhardtii/1-344            VRMSVDAHDATGGIALKVEEAAAVAR-MGIPVLIAQAGSEHGD-------AAC----RLG
Volvox_carteri/1-321                       VRMTADAHDVTGGIALKVEEAARVAR-LGVPVIIAKAGSEDGA-------AAC----RLG
Micromonas_pusilla.CCMP1545/1-411          IETSTSANDVTGGINTKIESAVSIAHYRDVSVYIAAAGTRHGDAAIRGKVLTKGGPDAND
Micromonas_pusilla.RCC299/1-402            TVSLADNGDVTGGIKTKVQEAAAIAL-KGVDVFLTNHQDEDIV-------SVLYGHHEND
Ostreococcus_lucimarinus/1-352             FAIDDAIADVTGGVRAKLTTAIAIAS-YLPPGPPRVFLARPGA-------FAA---EPSR
Ostreococcus_tauri/1-356                   FKTADGVTDVTGGIEAKLGAALAIAR-ALPGASPSVFLTRAGV-------LTA---ADD-
Entamoeba_hisolytica/1-259                 FLHGCKGIDVTGGMAGKINELMIAAT-KHNIQSYVFKGTKECL-------ELF---LEG-
Entamoeba_invadens/1-257                   YLGASDGVDVTGGMAGKIKELIEAAE-DNDTISFVFSGDEKSL-------TKF---LDG-
Roseiflexus_castenholzii/1-269             GTGASHGVDVTGGMRSKVELMWRLVQ-AIPSLQVYFIGPTPGLLH-----RAL----LGD
Herpetosiphon_aurantiacus/1-267            RLGGSHGVDVTGGMRNKVEAMWQLVQ-QAPQLEIWICGPQQLQ-------SAL----SG-
Desulfurococcus_kamchatkensis/1-262        QLSKEKNNDATGGIYGKVKSMHELSE-RLGVRVIIASGFSRQDVVN----AIL----HG-
Thermosphaera_aggregans/1-261              LLESDASRDVTGGMYSKVKTMSELSR-KTGAKVIVTSGFNIDHVV-----QAL----RG-
Staphylothermus_hellenicus/1-266           RLKETMYMDETGSIYGKVKYMASLSK-KLRIPVFIVSGHDVENAVN----AIL----YG-
Hyperthermus_butylicus/1-260               AVGGSDKLDVTGGMRRKLEAIRANWC-EGLSRVVIVYGLEPNN-----IEQAV----LG-
Aeropyrum_pernix/1-249                     ------SGDATGGMARKVKAALEASR-RGVSRVVIVGGDMLL--------EAL----RG-
Ignicoccus_hospitalis/1-236                ---GSEGIDVTGGMRKKLEVLEEAAR-YAEVCIFNGLKVVNFE-------KFL----KG-
Thermofilum_pendens/1-259                  ----THGVDVTGGLREKLAEAFYAAK-NGVRVCIGGVNFIE---------KMV----KGE
Methanocaldococcus_jannaschii/1-260        YLSGSNSIDVTGGMKYKIDMIRKNKC-RGFVFNGNKANNIY---------KAL----LGE
Methanococcus_maripaludis/1-257            SLKPSNKEDVTGGMHLKVMECYNLGI-KTIIFNGNKKRNIY---------NAL----LKN
Metallosphaera_sedula/1-224                --LPSLSYDLTGGMREKVRKILQNNI-NAMIFNGKKRGNVF---------NAL----KG-
Sulfolobus_solfataricus/1-241              ---QTNYYDITGGILSKIKKIFENNL-NALIFDGSKTGNIY---------LAL----RG-
Archaeoglobus_profundus/1-239              ----SKGVDVTGGMRSKVEKILRSGV-NARIFSISKFK------------GFL----SC-
Methanospirillum_hungatei/1-256            --EGSGSVDVTGGMQGKISELLRLAD-IGIESDIFHISRLQ---------DFL----SG-
Methanosarcina_mazei/1-260                 ----SGSTDVTGGMLGKVLELLELSK-NSSITSYIFNAGKADN-----IYRFL----NG-
Methanosaeta_thermophila/1-248             HLLPAKGVDVTGGMRGKLAELVELAG-IGIDSRIFNAGVAGNVR------RAL----SG-
Nitrosopumilus_maritimus/1-247             ----ENKMDVTGGMTRKIEEASKISK-MGMNVFFVNGNKPERIV------KAV----KNR
Cenarchaeum_symbiosum/1-246                ----EAGMDVTGGMGRKIEEGRKIAR-GGTKVFLVNGKKPRRILD-----AAL----KG-
Ferroplasma_acidarmanus/1-280              ----TINPDVTGGIMNKYNKMKLISD-IGIPVYLINGLYPERIY------NIG----KD-
Picrophilus_torridus/1-242                 -NFESKYNDVTGGMKSKLDIMKRIAR-LGVKVYLINGNYPERIY------DLN----ND-
Pyrococcus_furiosus/1-256                  KLEGSAGIDVTGGIKKKLEAVSELVH-YTEEVWLINGLVKDRLS------MAI----VG-
Thermococcus_barophilus/1-266              -SSESAGIDVTGGIGNKLREALKIAE-HSEVYFINGKVRGNLG-------KAI----KG-
Chloroflexus_aggregans/1-268               GAGASRAADVTGGMRSKLELMWQLVE-TLPDLEVRLIGPDPSLLT-----AAL----LGQ
Pseudomonas_syringae/1-261                 CVGASSMTDLTGGMRNKFMQQRQLAR-L--GVVSEVLSFECFD---RVHLSLC-----GL
Shewanella_denitrificans/1-279             AIFATGRLDVTGSMKGKVDAAMRLAE-MEVSSVICSAATFLAT----GVSDIC----SGN
Streptomyces_wedmorensis__fomA/1-266       ALWGSSEWDATGAMHTKLDALVTCAR-R--GAECFIMRGDPGS-------DLE-----FL

Anolis_caroliensis-lizard/1-281            LMEGE----------------------------GTKLFFKEA------------------
Callorhinchus_milii-shark/1-249            VRKNG----------------------------GTELSIEGS------------------
Saccoglossus_kowalevskii/1-283             LLTDS----------------------------GTLITMKDVLPNKQS------------
Branchiostoma_floridae/1-298               LPQGT----------------------------GTIVQLNISKHPDEVT-----------
Strongylocentrotus_purpuratus-urchin/1-280 RSDEFK---------------------------GTIIEAGE-------------------
Paracentrotus_lividus-sea_star/1-236       ------------------------------------------------------------
Lottia_gigantea/1-283                      LDEKTFDSTKISLKK---------------------------------------------
Nematostella-anemone/1-278                 VEKGQ----------------------------GTSIGLCETAQGKNK------------
Acropora_palmata/1-176                     ------------------------------------------------------------
Montastraea_faveolata/1-203                LD-----------------------------GNGTEILAETQEFNRVNIQ----------
Trichoplax_adhaerens/1-281                 LEESDIATTITLQESEYPK-----------------------------------------
Rhizopus_oryzae/1-279                      ---------------------------------GTKEALDMMTLQNTFTDKMTRFSLF--
Mucor_circinelloides/1-306                 ---------------------------------GTDEALDIMALKVELTPNALMTIFTRD
Epichloe_festucae/1-297                    DVDGS-------------------------DRCGEIAHTWTTILR---------------
Spizellomyces_punctatus/1-299              LLN-----------------------------IGTTMLGDDQA-----------------
Phaeodactylum_tricornutum/1-361            D-----------------------------IERATILFR---------------------
Thalassiosira_pseudonana/1-355             ------------------------------------------------------------
Perkinsus_marinus/1-320                    --------------------------------AGTKSALEAME--GREPEICTVVVP---
Polysphondylium_pallidum/1-283             N--NDHSS------------------------VGTVITNK--------------------
Dictyostelium_discoideum/1-292             -LIDDPINIKK----------------------GTF------------------------
Arabidopsis_thaliana/1-332                 LRDS-VPE----------------------DWLGTIIRFSK-------------------
Rice/1-335                                 TSSE--------------------------DWLGTVIRSSR-------------------
Maize/1-340                                VSTDSE------------------------DWLGTIIRSSK-------------------
Poplar/1-335                               LP---------------------------EDWLGTVIRFVGKGNSNC-------------
Ricinus_communis/1-337                     IP---------------------------DDWLGTVIRFVAKGTGSTC------------
Vitis_vinifera/1-340                       NVP--------------------------EEWLGTVIRLLR-------------------
Soybean/1-335                              IP---------------------------DDWLGTVVRSLR-------------------
Selaginella_moellendorffii/1-312           IEQ---------------------------NWIGTIVRKADST-----------------
Physcomitrella_patens.2/1-351              VKNLKSNK----------------------SWTGTLIRNALGQTS---------------
Chlamydomonas_reinhardtii/1-344            PQVAATAAGGEGGGEGEGGGVAAASGSPPATWRGTLVVLEGC------------------
Volvox_carteri/1-321                       PQVAD----------------------------GTATSSAGLAASAVSEPGSRLGPLASC
Micromonas_pusilla.CCMP1545/1-411          LTIEELDG---------------------YQWFGTFIRKAQPGEPDYVRPIVTARKCLTR
Micromonas_pusilla.RCC299/1-402            SRFEP-------------------------HWFGTHVKMLAPEVENEPPA----------
Ostreococcus_lucimarinus/1-352             VSDHALNAILAFDARGAPDDA------ALNNFIGTAIARRAP------------------
Ostreococcus_tauri/1-356                   ARDHALDAVLGRPNR--TDDS------KFP-FVGTVVRTDRQRESS--------------
Entamoeba_hisolytica/1-259                 ------------------------------NDVGTKICQ---------------------
Entamoeba_invadens/1-257                   ------------------------------EDVGTKVCK---------------------
Roseiflexus_castenholzii/1-269             A-----------------------------TVEGTVMVAG--------------------
Herpetosiphon_aurantiacus/1-267            ----------------------------QLNGPGTIIKLD--------------------
Desulfurococcus_kamchatkensis/1-262        -----------------------------DISEGTIIEP---------------------
Thermosphaera_aggregans/1-261              ----------------------------IVPEKATIIEIV--------------------
Staphylothermus_hellenicus/1-266           -----------------------------KVLRGTVIDMS--------------------
Hyperthermus_butylicus/1-260               -----------------------------AAGVGTEILP---------------------
Aeropyrum_pernix/1-249                     ------------------------------VNVGTRVDAR--------------------
Ignicoccus_hospitalis/1-236                ------------------------------ECPGTRVVPCRQG-----------------
Thermofilum_pendens/1-259                  ------------------------------EAPYTVVKA---------------------
Methanocaldococcus_jannaschii/1-260        V-------------------------------EGTEIDFSE-------------------
Methanococcus_maripaludis/1-257            -------------------------------VKGTLIN----------------------
Metallosphaera_sedula/1-224                -------------------------ERIGTLIKVSR------------------------
Sulfolobus_solfataricus/1-241              ------------------------------YNIGTLVRGNPNA-----------------
Archaeoglobus_profundus/1-239              ------------------------------EEVGTLVKSD--------------------
Methanospirillum_hungatei/1-256            -----------------------------ADHGGTRILPEGA------------------
Methanosarcina_mazei/1-260                 ------------------------------ESIGTRISPDKRV-----------------
Methanosaeta_thermophila/1-248             ------------------------------ESLGTLITGR--------------------
Nitrosopumilus_maritimus/1-247             ------------------------------KFEGTLFRGK--------------------
Cenarchaeum_symbiosum/1-246                ------------------------------SFAGTIIRGHK-------------------
Ferroplasma_acidarmanus/1-280              ------------------------------NFTGTVV-----------------------
Picrophilus_torridus/1-242                 ------------------------------DFIGSVIE----------------------
Pyrococcus_furiosus/1-256                  ------------------------------NGIGTIVRP---------------------
Thermococcus_barophilus/1-266              ------------------------------QRVGTRLRKLEHPKIS--------------
Chloroflexus_aggregans/1-268               P-----------------------------LTNGTLIKQ---------------------
Pseudomonas_syringae/1-261                 ------------------------------RQFGTVFLSE--------------------
Shewanella_denitrificans/1-279             -------------------------------ISGTHFNSSEKVGLNPKMEELC-------
Streptomyces_wedmorensis__fomA/1-266       TAPFSSWPAHVR---------------------STRITTTASA-----------------
