# Supplementary figures and images for: Discovery of a metabolic alternative to the classical mevalonate pathway
Source: eLife. 2013 Dec 10;2:e00672. doi: 10.7554/eLife.00672 (PMC3857490; doi:10.7554/eLife.00672)

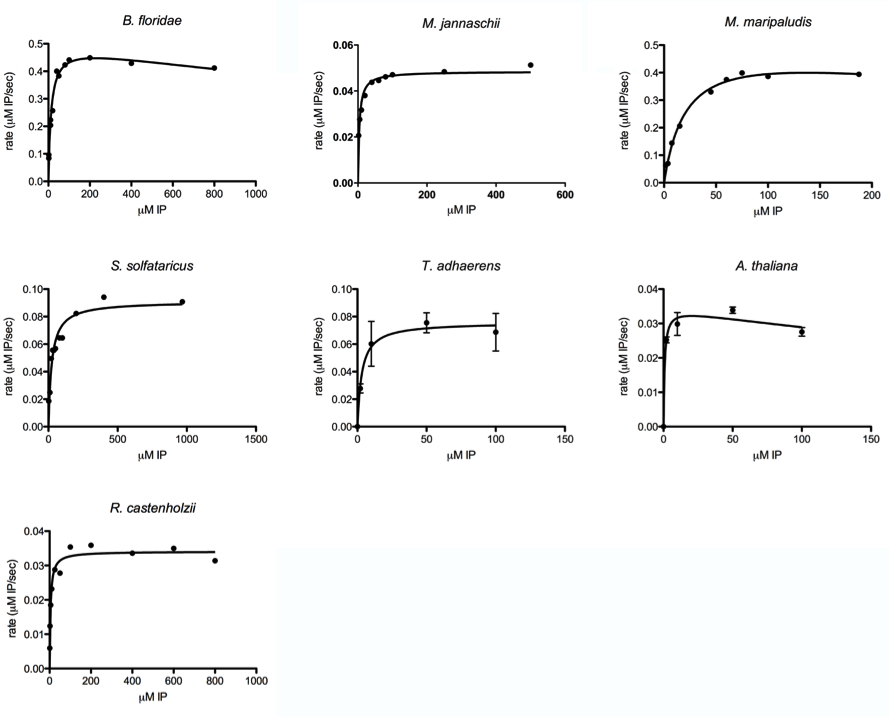

Supplement: Table 3—source data 1. — Steady-state kinetic plots for IPKs listed above each curve. DOI: http://dx.doi.org/10.7554/eLife.00672.010 [file elife00672s003.png]

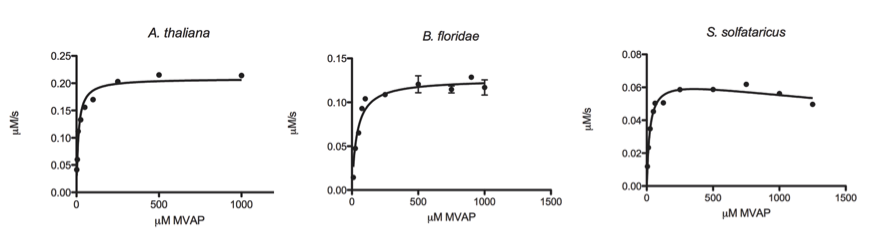

Supplement: Table 3—source data 2. — Steady-state kinetic plots for PMKs listed above each curve. DOI: http://dx.doi.org/10.7554/eLife.00672.011 [file elife00672s004.png]

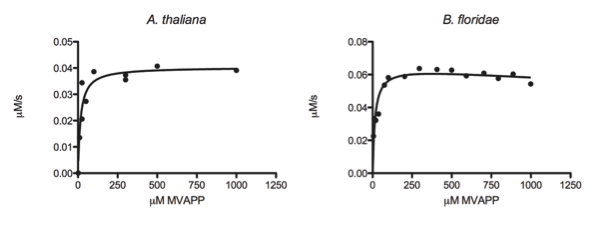

Supplement: Table 3—source data 3. — Steady-state kinetic plots for MDDs listed above each curve. DOI: http://dx.doi.org/10.7554/eLife.00672.012 [file elife00672s005.png]

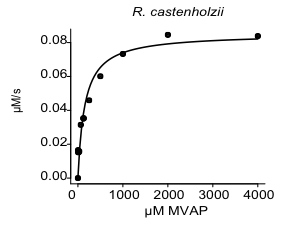

Supplement: Table 3—source data 4. — Stead-state kinetic plots for MPD from R. castenholzii. DOI: http://dx.doi.org/10.7554/eLife.00672.013 [file elife00672s006.png]
