# Supplementary material for: Discovery of a metabolic alternative to the classical mevalonate pathway
Source: eLife. 2013 Dec 10;2:e00672. doi: 10.7554/eLife.00672 (PMC3857490; doi:10.7554/eLife.00672)
Supplement: Table 4—source data 1. — Amino acid sequence alignments of archaeal MDDs. DOI: http://dx.doi.org/10.7554/eLife.00672.018 [file elife00672s007.rtf]

Table 4 - Source data 1. Amino acid sequence alignments of archaeal MDDs.MDC-Ss/1-325                              ML----------------------------------------------------------Haloquadratum_walsbyi_/1-324              MK----------------------------------------------------------Thermoplasma_volcanium_/1-359             MDLPGIVS------------------------------------LLKEKGLYRAPGKYRKMetallosphaera_sedula/1-323               MK----------------------------------------------------------Halobacterium_sp._NRC-1/1-334             MR--------------------------------------------------------ATSulfolobus_tokodaii_/1-257                MK----------------------------------------------------------Thermoplasma_acidophilum/1-384            MA-PHLMASGKSWHDSIYTYNQY-----------YHVQPFDMEKLLRDRGIYRRPGMHAIThermoplasma_acidophilum/1-405            MDTAGMEHSVSWISSNSDELILRSWISSRMDIAELHRAGDDIKEMMKSEGYYSEPSKYEAHalobacterium_salinarum_R1/1-325          MK----------------------------------------------------------Halorubrum_lacusprofundi_/1-332           MT----------------------------------------------------------Candidatus_Micrarchaeum_acidiphilum/1-353 ME----------------------------------------------------------Halorhabdus_utahensis/1-323               MK----------------------------------------------------------Ferroplasma_acidarmanus_/1-364            MD--------------------------------FKFEGENIKHKLVDMDLYKELINYDTHalomicrobium_mukohataei/1-323            MK----------------------------------------------------------Haloterrigena_turkmenica/1-327            MK----------------------------------------------------------Natrialba_magadii_/1-327                  MK----------------------------------------------------------MDC-Ss/1-325                              -----KSVTVSAPSNIAVVKYWGKRGDERLNLPLNNSLSITLDDQLSVITKVTLN---DKHaloquadratum_walsbyi_/1-324              -------ATARAHPIQGLIKYHGMR-DSDKRYPYHDSISVCT-APSATTTTVEFQSDASGThermoplasma_volcanium_/1-359             EMRYGEIHYGSGYPITGLIKFLGYY-DESLKIANFPSISLNT-DVSEAYSAFMISKDNGNMetallosphaera_sedula/1-323               -----LEAEAVAPSNIAIVKYWGKR-DRELNLPLNSSLSISL-DSLWVRSRVIFDESLDKHalobacterium_sp._NRC-1/1-334             PPHRRMKATARAHPIQGLVKYHGMR-DESLRMPYHDSISVCT-APSNTTTTVEFDPDRDASulfolobus_tokodaii_/1-257                ------------------------------------------------------------Thermoplasma_acidophilum/1-384            RPAYRKIAFGAGYSEIGIIKSLGYY-DSTFNILNFPCVSMAA-DFSRAYAALVLTENIGEThermoplasma_acidophilum/1-405            ELSDGNITFAYSYPIKAFEKFLGYY-DRENRIAFNPSISMRT-DFSFCLAACRYRKNGKTHalobacterium_salinarum_R1/1-325          -------ATARAHPIQGLVKYHGMR-DESLRMPYHDSISVCT-APSNTTTTVEFDPDRDAHalorubrum_lacusprofundi_/1-332           -----EKATARAHPIQGLVKYHGMR-DPELRLPYHDSISLCT-APTATTTTVEWQPDASECandidatus_Micrarchaeum_acidiphilum/1-353 ----DRIYTAIGSSNIAFIKYWGKR-DGKINLPNNSSISMTLDRNVGTKTSVLFSSKLKSHalorhabdus_utahensis/1-323               -------ATATAHPIQGLVKYHGIR-DPELRTPYHDSISLCT-APSNSTTTVAFEPERPEFerroplasma_acidarmanus_/1-364            ELEPGKVSTGHSYPIKAFEKFLGYY-DDYYKIANNPSTSFNT-DFSFVYSACMYTKKENSHalomicrobium_mukohataei/1-323            -------ATAKAHPIQGIVKYHGMR-DEELRLPYHDSISVCT-APSHSKTTAAFDPELDAHaloterrigena_turkmenica/1-327            -------ATAMAHPIQGLVKYHGMR-DEIERLPYHDSISLCT-APSHTRTTVEFSMDYEENatrialba_magadii_/1-327                  -------ATAMAHPIQGLVKYHGMR-DDIERLPYHDSISVCT-APSHTRTTVEFSMDYDEMDC-Ss/1-325                              NIVIVNDR----ILSEDEMKEYAGRVLDTFKKIVGKEF---HVKVESKSKFPIN--AGLAHaloquadratum_walsbyi_/1-324              DVYIIDNE-----RVDGRAAERIDAVVEHVRERTGIRD---PVRLVSTNSFPSN--IGFGThermoplasma_volcanium_/1-359             DTAVVEGE------NSPNITKKAMTAINVFKNLYDIKG-SFHFYLRIKRKYAGA--KGLGMetallosphaera_sedula/1-323               DEVIINGK----RLSENEVREYAGRVLRRFRDLYGKEL---FARVESTTNFPSS--AGLAHalobacterium_sp._NRC-1/1-334             DQYVVDGD-----TVTGHGADRIRSVVDAVRDRAGFDH---RVRLESQNSFPTN--IGLGSulfolobus_tokodaii_/1-257                --------------------EYSGRVLNIFRKLYGKEI---YAKVESWSNFPKS--TGLAThermoplasma_acidophilum/1-384            DTFILNGR------QDEQTMIMARRVVQLLRSIYSIRG-SFHVYIKVDNKQGGG--CGHWThermoplasma_acidophilum/1-405            DTVVLDGY------ADNKYYKKAKFALDKFRSEYSING-SFDFYIKRYRRYQKA--KGLSHalobacterium_salinarum_R1/1-325          DQYVVDGD-----TVTGHGADRIRSVVDAVRDRAGFDH---RVRLESQNSFPTN--IGLGHalorubrum_lacusprofundi_/1-332           DVYVIGDE-----EVDGRAAERIDMVVEHVRELAGVDA---AVRLESENSFPSN--IGFGCandidatus_Micrarchaeum_acidiphilum/1-353 DRLFINGKEENIKEGANEKSRFISEMLAYCKKAAGINT---NALIVSENNFPSD--SGLAHalorhabdus_utahensis/1-323               DEYVIDGE-----HIDGRGAERIRTVVDNVRERADLDE---RVRVASENNFPSN--VGFGFerroplasma_acidarmanus_/1-364            DMAILDGK------PATGYVDRYEKPLEIFRKNTGIRG-SFIFYIKRYRKYSEA--KGLSHalomicrobium_mukohataei/1-323            DEYVIDGE-----PVEGRGAERIAAVVDHVRELAGIDH---RVRFESENTFPTN--IGFGHaloterrigena_turkmenica/1-327            DTFVVDGE-----ELDGRAYERVEAVVEKARSKSDAAHTVYPVRLESENSFPSN--VGLGNatrialba_magadii_/1-327                  DTYVVDGE-----ELEGRAAERVDAVVEKARDMSDAAHTVYPVRLESENSFPTN--VGLGMDC-Ss/1-325                              SSAAGIAALAFSLNELLELN----LKSEELSKIARLGSGSACRSMFGGFVVW--NKGEREHaloquadratum_walsbyi_/1-324              SSSSGFAAAAMALVTAAGEE----LTHPEISTIARRGSSSAARAVTGAFSQL--YSGMNDThermoplasma_volcanium_/1-359             ESAAVAAAASRSLVSAL-FEKEALKDSNFISIVARLASGSGSKSVAGPLSLWLTAPAVSHMetallosphaera_sedula/1-323               SSAAGIAALTYASNAALGLG----LSNRELSKIARVGSGSACRSMFGGFVKW--NRGELEHalobacterium_sp._NRC-1/1-334             SSSSGFAAAALACVRAAGLD----LDLPTVSTVARRGSASAARAVTGGFSDL--HAGLNDSulfolobus_tokodaii_/1-257                SSAAGIAALVYATNEALELG----LSQKELSKIARIGSGSACRSTAGGFVLW--EKGERDThermoplasma_acidophilum/1-384            ESAAVAAAFARSVARSV-FDESAIEDGPFLSKMARLVSGSGAASTTGPLSVLISWPGYAHThermoplasma_acidophilum/1-405            ESSAVAAAVSRALISNV-FGDDAAKDDIFVSRYARLVSGSGTRAAHDGISMWLSYPGMDSHalobacterium_salinarum_R1/1-325          SSSSGFAAAALACVRAAGLD----LDLPTVSTVARRGSASAARAVTGGFSDL--HAGLNDHalorubrum_lacusprofundi_/1-332           SSSSGFAAAALALTEAAGLD----LTLPDISTVARRGSSSAARSVTGAYSRL--DAGLNDCandidatus_Micrarchaeum_acidiphilum/1-353 SSASGGATLAFLLSNALDLK----MDSREISIMARKISGSACRSVYGGIVKW--DAGSKQHalorhabdus_utahensis/1-323               SSASGFAALATALVEAAGLD----LSRPEISTIARRGSTSAARAVTGGFSDL--RAGSNDFerroplasma_acidarmanus_/1-364            ESSAVASAVARSLIKNV-FGEMGAKDDSFVSRYARLVSGSGTRAAINGPSIWLSYPGMQEHalomicrobium_mukohataei/1-323            SSASGFAAAAMALVEAAGLD----MTRPEVSTVARRGSCSAARAVTGGFSHL--KNGMNDHaloterrigena_turkmenica/1-327            SSSSGFAAAAMALAEAAELD----ASRQEISTIARVGSASAARAVTGAFSQL--HTGLNDNatrialba_magadii_/1-327                  SSSSGFAAAAMALAEAADLD----ADRPTVSTIARVGSASAARAVTGAFSQL--NTGLNDMDC-Ss/1-325                              DGEDSYCYQI------FRHDYWSELVDIIPILSEKEKKISSRKGMIRSAETSELMECRLKHaloquadratum_walsbyi_/1-324              --TDCHAERI------ETDLD--ATVRTVAAHVPAYKETEEAH---REAAQSHMFDARLAThermoplasma_volcanium_/1-359             --EGSFSLNL------RKEI---DDIFLCAVPIRDSVSTAEAH---NTVIKSPFYQQWSRMetallosphaera_sedula/1-323               SGDDSFCEEI------FPPDHWPDLVDIIPIFGEEKKKVSSRTGMENTATSSALMRCRLQHalobacterium_sp._NRC-1/1-334             --ADCRSERL------DAPAEFASDLRIVVGEVPAYKETESAH---AEAADSHMFDARLASulfolobus_tokodaii_/1-257                DGEDSYCYSL------FPENHWKELVDIIAIVSEKSKKISSREGMIITAKTSNLMKCRLKThermoplasma_acidophilum/1-384            --DTSFALGL-----PMPD----TGIALCAVPIAADFHRSNIH---EVALRSPFYREWATThermoplasma_acidophilum/1-405            --RDCVAFKV-----GKSN----ENLNYGVFPKYSDVATDNAH---SIAVNSVFYGTWVSHalobacterium_salinarum_R1/1-325          --ADCRSERL------DAPAEFASDLRIVVGEVPAYKETESAH---AEAADSHMFDARLAHalorubrum_lacusprofundi_/1-332           --EDCRSHRLDVGV-GDDGFDPEEDLRIVAAHVPAYKETEEAH---REAAASHMMQARTACandidatus_Micrarchaeum_acidiphilum/1-353 DGSDSFAEQV------VDHRYWPDLMDIIAIVDPSKKKVSSSAGHAITVKTSSLYRVRPQHalorhabdus_utahensis/1-323               --ADCRSKRL------DVPLE--DDVRIVGAVIPAYKETEAAH---EEAAESHMFEGRLAFerroplasma_acidarmanus_/1-364            --QNSFAVKI------PADV---DKINYAIFPKNIDYRTSNAH---IEAVKSIFYNSWLNHalomicrobium_mukohataei/1-323            --ADCRSERI------ETELE--EDLRVVAGMVPSYKETEAAH---EEAAASHMFENRMAHaloterrigena_turkmenica/1-327            --EDCRSRRI------PSDLH--EDLKIVVGLVPYHKETEDAH---REAADSHMFQARNANatrialba_magadii_/1-327                  --EDCRSERL------PSNLH--EDLKIVVGLVPYHKETEDAH---NEAEDSHMFQARNAMDC-Ss/1-325                              YIEKTFNEVIEAIRNRDEKKFYYLMMRHSNSMHAVILDSWPSFFYLNDTSIRIMEWIHDYHaloquadratum_walsbyi_/1-324              HVHHQIDAMRDALYNADFDRIFELAEHDSLSLTAATMTGPAGWVYWQPQTIAVFNTVRELThermoplasma_volcanium_/1-359             LQFDAVYSIIS--RGGYSAQIIENATTNTYLMHSVLIST--GKLLWNQDTLRAMGIVEDMMetallosphaera_sedula/1-323               FIEETFNEVIDAIRTKNAGKFFQLTMRHSNSMHAVILDSWPPMNYLNEKSFRVMEWVVEFHalobacterium_sp._NRC-1/1-334             HVQGQLAEMRDAVRAGDFQRVFETAEHDSLSLAATTMTGPSGWVYWKPETLSIFETVRELSulfolobus_tokodaii_/1-257                FIEETLPKVIKSIEERNEKEFYYWLMRHSNSMHAVILDSWPSFFYLNDTSLKIMEWIQEFThermoplasma_acidophilum/1-384            YTRHALLDLLN--SEFDADTIIRTATNSSLMMHATLMSS--RSILWTEKTIDVVSRILEMThermoplasma_acidophilum/1-405            EKFSNVKRLIS--DHFDINDLLKIGENDMLRLNSILMSG--GLIIQTPDSLRILKEILKFHalobacterium_salinarum_R1/1-325          HVQGQLAEMRDAVRAGDFQRVFETAEHDSLSLAATTMTGPSGWVYWKPETLSIFETVRELHalorubrum_lacusprofundi_/1-332           HVQDQLVEMTDALREGDFDRICGTAEHDSLSLTATTMTGPAGWVYWQPETIAVFNAVRELCandidatus_Micrarchaeum_acidiphilum/1-353 VAEEGVKKVVNAVTNKDFQVLAETVMRDSNNMHATMMDSWPPIMYLSDASRSIIYAMHELHalorhabdus_utahensis/1-323               HVHEQLADMRDALGRGDFERSFEIAEHDTLSLAATTMTGPSGWVYWQPESLEVFETVRDLFerroplasma_acidarmanus_/1-364            EKYNKLNEIID--EDFNIELMMNRAMEDMFALNAVLLSR--GNVIQTAESIILLKNFIEFHalomicrobium_mukohataei/1-323            HIHGQIAEARDAIAAGAFDRTFELAEHDSLSLAATTMTGPAGWVYWQPRTIEIFNAVRELHaloterrigena_turkmenica/1-327            HIHGQIAEMRDALRNNEFDRAFELAEQDSLSLAATTMTGPSGWVYWQPATLKIFNTVRELNatrialba_magadii_/1-327                  HIHSQIAEMRDALRNDEFEDAFELAEHDSLSLAATTMTGPEGWVYWQPATLAIFNRVRELMDC-Ss/1-325                              GK-------AGYTFDAGPNPHIFTTERN-IGDILEFLKSLEIKRIIV--SKVGDGPKVLSHaloquadratum_walsbyi_/1-324              RERESIP--VYFSTDTGASVYVNTTAAH-VDTVESAISDIGIDTDI---WTVGGPATVLSThermoplasma_volcanium_/1-359             RRIGRL---IGFSIDTGPSVLVMADREDLIKEFKERYNGECIDASVP-NGAPDIPSSFVEMetallosphaera_sedula/1-323               GK-------AAYTFDAGPNPHIFVLEKD-VDEVLKFLNEIGSTKTIV--SRVGKGPYLI-Halobacterium_sp._NRC-1/1-334             RA-DGVP--TYFSTDTGATVYVNTTASH-ADEVEAAVADCGVDTAV---WEVGGPAHELDSulfolobus_tokodaii_/1-257                GK-------AGYTFDAGPNPHIFTTEKY-KDEVIRFLNSIGVNKIII--SKVGSGPKVNKThermoplasma_acidophilum/1-384            RSGGRA---VGFSIDAGPSVVLMARNAAELDEARRSIDAECVDGSIT-SGEPSIPPEFLRThermoplasma_acidophilum/1-405            KSKNEG---FYFTADTGPSIAIFSFDRSLIDEFRENVNDEYIEGSYDFKGYNNRMRDFIRHalobacterium_salinarum_R1/1-325          RA-DGVP--TYFSTDTGATVYVNTTASH-ADEVEAAVADCGVDTAV---WEVGGPAHELDHalorubrum_lacusprofundi_/1-332           RE-EGVP--VYFSTDTGASVYVNTLAGH-AEEVEERIAEIGIDTDI---WEVGGPAHLLDCandidatus_Micrarchaeum_acidiphilum/1-353 NESEGKYV-AAYTFDAGPNAHIITTSSN-RSKVIKMLEEIGVARSII-ESKMGAGPEMLEHalorhabdus_utahensis/1-323               RD-DGVP--VYFSGDTGASIYVNTTAEY-VDRVESAIETLGIETLT---WRVGGPARVRDFerroplasma_acidarmanus_/1-364            SKKHEG---IYITGDTGPSLMVMSGDKTLLNQFLDTVDDPKIVGSHHPKDHKKRENEFRKHalomicrobium_mukohataei/1-323            RE-EGVP--VYFSVDTGASVYVNTTAEH-VDRVEETVADCGVDTRV---WEVGGPARVLDHaloterrigena_turkmenica/1-327            REEEDIP--VYFSTDTGASVYVNTTEEH-VDEVEEAVSDCGVSTTV---WDVGGPAKLLDNatrialba_magadii_/1-327                  REEEDIP--VYFSTDTGASVYVNTTEEH-AEFVEEEIADCGVSTTI---WDVGGPARLLDMDC-Ss/1-325                              RE----------------Haloquadratum_walsbyi_/1-324              ASDSLF------------Thermoplasma_volcanium_/1-359             SAERYFAKH---------Metallosphaera_sedula/1-323               ------------------Halobacterium_sp._NRC-1/1-334             ERDAIF------------Sulfolobus_tokodaii_/1-257                LL----------------Thermoplasma_acidophilum/1-384            MAREALDRYT--------Thermoplasma_acidophilum/1-405            EAQEYFTQTPGEDEEDRLHalobacterium_salinarum_R1/1-325          ERDAIF------------Halorubrum_lacusprofundi_/1-332           ENEALF------------Candidatus_Micrarchaeum_acidiphilum/1-353 GEESLIDQESMAPVHK--Halorhabdus_utahensis/1-323               PEKALF------------Ferroplasma_acidarmanus_/1-364            ESEEYFRSI---------Halomicrobium_mukohataei/1-323            DSEALF------------Haloterrigena_turkmenica/1-327            EEKHLF------------Natrialba_magadii_/1-327                  EDEHLF------------
