# Supplementary material for: Discovery of a metabolic alternative to the classical mevalonate pathway
Source: eLife. 2013 Dec 10;2:e00672. doi: 10.7554/eLife.00672 (PMC3857490; doi:10.7554/eLife.00672)
Supplement: Table 4—source data 2. — Amino acid sequence alignments of bacterial MDDs. DOI: http://dx.doi.org/10.7554/eLife.00672.019 [file elife00672s008.rtf]

Table 4 - Source data 2. Amino acid sequence alignments of bacterial MDDs.Saureus/1-327                      ----------------MIKSGKARAHTNIALIKYWGKKDEALIIPMNNSISVTLEKFYTEStaphylococcus_warneri_/1-327      ----------------MAKSGKARAHTNIALIKYWGKADEKLIIPMNNSLSVSLDKFYTEStaphylococcus_carnosus/1-331      ----------------MAKTGKARAHTNIALIKYWGKADETLIIPMNNSISLTLDKFYTEEnterococcus_faecalis/1-331        -----------------MLSGKARAHTNIALIKYWGKANEEYILPMNSSLSLTLDAFYTELactobacillus_iners/1-325          ------------------MKIIARAHTNIALIKYWGKADSSLKIPLMSSISMTLDAFYTEListeria_monocytogenes_/1-339      --MLELHKNGFLRLEKVVMKATAIAHTNVALIKYWGKRDEHLILPANSSLSFTVDKFYTKLactobacillus_johnsonii/1-321      ------------------MKKTARAHTNIALIKYWGKADQALKTPLMSSLSMTLDAFYTDLactobacillus_reuteri_/1-323       -------------------MATAKAHTNIALVKYWGKKDQELIIPQTDSLSLTLNEFYTTLactobacillus_gasseri/1-321        ------------------MKKTARAHTNIALIKYWGKANQALKTPLMSSLSMTLDAFYTDCorynebacterium_amycolatum/1-325   ----------------MTTTATAVAHPNIALIKYWGKRDEAVQLPATGSLSLTLGIAPTTLactobacillus_helveticus/1-320     ------------------MSKIVRAHTNIALIKYWGKANAELRLPLMSSLSMTLDAFYTDLactobacillus_acidophilus/1-320    ------------------MKNTARAHTNIALIKYWGKSDPILRLPLMSSLSMTLDAFYTDStreptococcus_pyogenes/1-314       ---------------MDPNVITVTSYANIAIIKYWGKENQAKMIPSTSSISLTLENMFTTBorrelia_garinii/1-312             ------------------MKVKCKVNASLALIKYWGKKDAFLNIPATSSLAVSVDKFYSIBorrelia_spielmanii_/1-312         ------------------MKIKCKANASLALIKYWGKKDVFLNIPATSSLAVSVDKFYSI|gb|EGF88987.1|/1-303              ------------------MDGYSLGYANIALVKYWGKKSKDPVLPYNPNISLRLDNLLSK|ref|YP_804426.1|/1-327            ---------------MNEKHGFARAHTNIALLKYWGKINSDLILPANDSISLTLDKFYTD|ref|NP_691147.1|/1-324            ------------------MKATAKAHTNIALIKYWGKRNEPIILPTNSNLSLTLDGFSTV|gb|ADQ43374.1|/1-351              MGAEQQATAVRTAPTGATDTATAVAHPNIALIKYWGKRDERLILPRTDSLSMTLDIFPTT|ref|ZP_07819449.1|/1-328          -------------MTADKYQARMRAHTNIALVKYWGKRNKNLFLPVTSSLSLTLDAFYTE|gb|AAO90151.1|/1-503              MRKQIFHQLLSQRSKSPQSSGHAFAPSNIALCKYWGKRNLELNLPVTSSLSISLGDKGAT|ref|ZP_06751493.1|/1-340          ------------------------------MIKYWGKRDEKLILPISPSLSLTLDSLYTD|ref|ZP_07086643.1|/1-352          --MTTQEFIGKENFTIHTQTVSESCPSNIALIKYWGKYADQ--IPANPSISYTLNHCKTNSepidermis/1-332                   -----------GSTGSMVKSGKARAHTNIALIKYWGKADETYIIPMNNSLSVTLDRFYTESaureus/1-327                      TKVTF--NDQLTQDQFWLNGEKVS--GKELEKISKYMDIVRNRAGIDWYA--------EIStaphylococcus_warneri_/1-327      TKVTF--DSDYPADQLILNGKEAN--EKETKKIQSYMDIVREIANTDLHT--------RIStaphylococcus_carnosus/1-331      TRVTF--DPTFEEDTFFLNGEKAN--EKETQKISAYLDIVRKQAGTHTKA--------SIEnterococcus_faecalis/1-331        TTVTF--DAHYSEDVFILNGILQN--EKQTKKVKEFLNLVRQQADCTWFA--------KVLactobacillus_iners/1-325          TEFTH--NVDLANDMVIMNGKAVD--DQASYRIINYVKKLQDIYGFNDHF--------CIListeria_monocytogenes_/1-339      TTVEW--DEKLTQDTFILNN---E--QKTDAKVARFIDKMREEFGISAKA--------KILactobacillus_johnsonii/1-321      TTFEH--DSSLTEDTFILNDQKQS--VEDSKRVFNYIHLLQEKFGMTDHF--------TILactobacillus_reuteri_/1-323       TTVNF--DNHLTSDLVAIDQQTLS--KKEAKKVVHVLDIVRQLSGIKAFA--------RVLactobacillus_gasseri/1-321        TTFEH--DSSLTEDTFILNDQEQG--PEESKRVFNYIHLLQEKFGFNDHF--------IICorynebacterium_amycolatum/1-325   TTVSLIDDPSVTADSGTLNGQEMV--GKDLSRVQKFLDLVRERAGSTSFA--------EVLactobacillus_helveticus/1-320     TSV----EKTNDENAFYLNGQKQD--SKQSQRVFSYLTKLQNKFGYHDNL--------IVLactobacillus_acidophilus/1-320    TLI----EKTDAKNEFYLNGKRQN--RQAKKRVFSYLDTLKEKFGYTDNL--------IVStreptococcus_pyogenes/1-314       TSVSFL-PDTATSDQFYINGVLQN--DEEHTKISAIIDQFRQPGQAFV----------KMBorrelia_garinii/1-312             SELEL-----SDQDEIILNSKPVV--FQNREKV--FFDYARKILSEPNVRF-------KIBorrelia_spielmanii_/1-312         SELEL-----SDRDEIILNSKPVI--LQNREKV--FFNYARKILGEPNVRF-------KI|gb|EGF88987.1|/1-303              TKIE---KSLSNEDEFYINDEKQG--PEEVNKMIKFISKFTPVAREKI----------CI|ref|YP_804426.1|/1-327            TEVTF--SDEYTSNLFYLNHQLID--VKKMQRINRVLEAVKSEFGYQGFA--------KI|ref|NP_691147.1|/1-324            TTVHF--QEELSSDEFFLNDRLVE--DAASQRVTGFLDKVRAMAGKEMYA--------RI|gb|ADQ43374.1|/1-351              TRVHL--APGADHDEVTLGGTPAE--GEARRRIVTFLDLVRERSGVADRA--------VV|ref|ZP_07819449.1|/1-328          TEVLF--DPSLKEDSFTLDGQVQT--GQSLAKVSNFVDLFRRDFNMSLPV--------QI|gb|AAO90151.1|/1-503              AAIS---PSSTNQHELIINNQPIAIYSTHAKQLLAFLEAF-NFLGVKY----------HL|ref|ZP_06751493.1|/1-340          TALM---PSSDGRWHFVLDGQEQG--GEALKRVVDFARIFPVSATAPIPSTPAAATPLTI|ref|ZP_07086643.1|/1-352          TSMEFVANEPFSVQTFLAGNEE----VKFAEKIEKYFRNIEQYLPWILKGK------YIISepidermis/1-332                   TKVTF--DPDFTEDCLILNGNEVN--AKEKEKIQNYMNIVRDLAGNRLHA--------RISaureus/1-327                      ESDNFVPTAAGLASSASAYAALAAACNQALDL--------QLSDKDLSRLARIGSGSASRStaphylococcus_warneri_/1-327      DSQNFVPTAAGLASSASAYAALAAACNEALQL--------ELSDKDLSRLARRGSGSASRStaphylococcus_carnosus/1-331      DSTNFVPTAAGLASSASAFAALAAACNEALEM--------RLSDKDLSRLARRGSGSACREnterococcus_faecalis/1-331        ESQNFVPTAAGLASSASGLAALAGACNVALGL--------NLSAKDLSRLARRGSGSACRLactobacillus_iners/1-325          KTENHVPTAAGLASSASGFAALATSFAASYNL--------NLNRQELSRIARLGSGSATRListeria_monocytogenes_/1-339      TSENHVPTAAGLASSASAFAALALAGSNAAGR--------KDTKEYISRLARFGSGSASRLactobacillus_johnsonii/1-321      RSTNHVPTSAGLASSASAFAALATSFAASYGL--------DLSKKELSRLARLGSGSATRLactobacillus_reuteri_/1-323       ESINHVPTAAGLASSASAFAALAGAASTAAGL--------NLSSRDLSRLARRGSGSATRLactobacillus_gasseri/1-321        KSTNHVPTSAGLASSASAFAALATSFAASYDL--------DLSRKDLSRLARLGSGSATRCorynebacterium_amycolatum/1-325   NSTNEIPTGAGLASSASGFGALALAAAKAYGL--------DYTPEQLSALARRGSGSACRLactobacillus_helveticus/1-320     KSVNHVPTSAGLASSSSAFAALAAAFCQYYNI--------QVDKKELSRLARIGSGSACRLactobacillus_acidophilus/1-320    KSTNHVPTSAGLASSSSAFAALAASFCKLYNL--------DVDKTELSRLARLGSGSASRStreptococcus_pyogenes/1-314       ETQNNMPTAAGLSSSSSGLSALVKACNQLFNT--------QLDQKALAQKAKFASGSSSRBorrelia_garinii/1-312             KSENNFPTAAGLASSSSGFASIAACILKYFN---------KYSFNSASNLARVGSASAARBorrelia_spielmanii_/1-312         KSENNFPTSAGLASSSSGFASIAACILKYFN---------KYSFNSVSNLARVGSASAAR|gb|EGF88987.1|/1-303              KSYNTVPTAAGLSSSSSGTMALVLACNEYFKL--------NKSTQEMVEIAKEGSGSSCR|ref|YP_804426.1|/1-327            ESENHVPTAAGLASSASGMAALAGAAVSALGS--------HTDLTNLSRLARLGSGSASR|ref|NP_691147.1|/1-324            HSLNHVPTAAGFASSASGFAALAAASTKAIGL--------ELNDTELSILTRQGSGSACR|gb|ADQ43374.1|/1-351              DTENTVPTGAGLASSASGFAALAVAAAAAYGL--------DLEAIALSRLARRGSGSASR|ref|ZP_07819449.1|/1-328          TSRNHVPTAAGLASSASAFAALAAASNQALGL--------GLSPEEVSVYARQGSGSASR|gb|AAO90151.1|/1-503              ELNFNIPLAAGLASSACAYAAIVKALDNFFEW--------QLDRKSLSILARLGSGSACR|ref|ZP_06751493.1|/1-340          ISHNHVPTAAGLASSSSAFAALAWALRDYFGLAGPGRDGRSLSDQALSACARQGSGSATR|ref|ZP_07086643.1|/1-352          RTENTFPHSSGIASSASGFGAIAK-CLMALDASFTEKTSEEESLRKASFLARLGSGSACRSepidermis/1-332                   ESENYVPTAAGLASSASAYAALAAACNEALSL--------NLSDTDLSRLARRGSGSASRSaureus/1-327                      SIYGGFAEWEKG-------YNDETSYAVPLESNHFED---DLAMIFVVINQHSKKVPSRYStaphylococcus_warneri_/1-327      SIFGGFAEWEKG-------HDDETSYAHPIDADHWED---ELSMIFVVINNQSKKVSSRSStaphylococcus_carnosus/1-331      SIFGGFAEWEKG-------HDDASSYSHPIDAEHWED---ELSMIFVVINNKSKKVKSRAEnterococcus_faecalis/1-331        SIFGGFAQWNKG-------HSDETSFAENIPANNWEN---ELAMLFILINDGEKDVSSRDLactobacillus_iners/1-325          SIFGGFVEWQKG-------YDDQTSFAFPINEHPQM----DLTMLAVELDVSQKDISSTCListeria_monocytogenes_/1-339      SVFGDFVIWEKG-E----LADGSDSFAVPFTNKLCD----KMSLVVAVVSDKEKKVSSRDLactobacillus_johnsonii/1-321      SVYGGFVEWKKG-------FDDESSYAVPIDENPDL----DLSLLAIEVNTKQKKISSTKLactobacillus_reuteri_/1-323       SIYGGLVEWQKG-------TDDASSFAQPVLENVDF----PIEMLAVLVDTKRKKVSSRSLactobacillus_gasseri/1-321        SIYGGFVEWQKG-------IDDASSYAIPIDENPDL----DLSLLALEVDTKQKKISSTKCorynebacterium_amycolatum/1-325   SIFGGLVEWLPG-------DDDASSHAVALPDSGL-----DLSLVVAVLAPGRKKIDSRALactobacillus_helveticus/1-320     SIFGGFSVWQKG-------DSDASSYAYALDEHPQM----DLHLLAVELNTNQKKISSTSLactobacillus_acidophilus/1-320    SIFGGFAIWQKG-------NSNQSSYAYALDEKPKM----DLQLLAVELNTEQKKISSTKStreptococcus_pyogenes/1-314       SFFGPVAAWDK---------DSGAIYKVETDL--------KMAMIMLVLNAAKKPISSREBorrelia_garinii/1-312             AIYGGFTILKEG-----------SKESFQLRDQSYFN---DLRIIFAIIDSNEKELSSRVBorrelia_spielmanii_/1-312         AIYGGFTILKEG-----------SKESFQLRDESYFN---DLRIIFAIIDSNEKELSSRA|gb|EGF88987.1|/1-303              SFYK-LAAWLED--------GSVEELSCKL----------DFGMMVLVVNEDRKKISSRV|ref|YP_804426.1|/1-327            SVFGGIVHWHRG-------YDHQSSFAEQIVSEDQI----DLNMVTIVIDRRQKKVKSTL|ref|NP_691147.1|/1-324            SIYGGFVEWQMG-E----KEDGSDSYAVPIASKDHW----DIRVAAVVLSATEKKVSSRD|gb|ADQ43374.1|/1-351              SLFGDFVVWHAGRETGTDEEADLSSYAEPVPTGPL-----DPALVVAVVNAGPKDVSSRA|ref|ZP_07819449.1|/1-328          SLFGGFALWHKG-Q----GDDSASSYAQQIDPADW-----DIAMLVVLVNPGPKKISSRQ|gb|AAO90151.1|/1-503              SVFNGFVEWYCG-K----DPDGMDSYAEPL-VENWP----GLCIGLCILNQKPKTVSSRE|ref|ZP_06751493.1|/1-340          SIFGGFVEWTYG-Q----REDGADSFARPIDDGEW-----DLGLIAVALSTGKKKISSRA|ref|ZP_07086643.1|/1-352          SLYNGLVVWGETDE----VEESSDLFGVQYPDTEIHEIFKNFNDWVLLIHEGQKSVSSTVSepidermis/1-332                   SIFGGFAEWEKG-------HDDLTSYAHGINSNGWEK---DLSMIFVVINNQSKKVSSRSSaureus/1-327                      GMSLTRNTSRFYQYWLDHIDEDLAEAKAAIQDKDFKRLGEVIEENGLRMHATNLGSTPPFStaphylococcus_warneri_/1-327      GMSLTRDTSRFYQYWLDHVDEDIKEAKQAIEAKDFKQLGEVIEANGLRMHATNLGSQPPFStaphylococcus_carnosus/1-331      GMSLTRNTSRFYQYWLDRVEADIEEAKAAIKDKDFKRLGEVFEANGLRMHATNLGAEPPFEnterococcus_faecalis/1-331        GMKRTVETSSFYQGWLDNVEKDLSQVHEAIKTKDFPRLGEIIEANGLRMHGTTLGAVPPFLactobacillus_iners/1-325          GMK-IAQTSPFYQTWLNRNKQEISEMESAIKNNNFTRLGELSELSANEMHSLNLTAMQSFListeria_monocytogenes_/1-339      GMRLTVETSPFFENWVSAAEIDLEEMKQAILDEDFIKVGEITERNGMKMHATTLGAEPPFLactobacillus_johnsonii/1-321      GMQ-LAQTSPFYQTWLARNEEEIAEIKKAIQNNDFTRIGELSELSANEMHACNLTAKEPFLactobacillus_reuteri_/1-323       GMQSSVETSPYYDAWRQVVANDMVAIKKAIKAKDIDQIGHIAEENALRMHALTFSADPGFLactobacillus_gasseri/1-321        GMK-LAQTSPFYQPWLARNKQEIAELKQAIKEKDFTKIGKLSELSANEMHACNLTANEPFCorynebacterium_amycolatum/1-325   AMRRTVETSPFFPAWVEQVPRDIEDMKAAIAAADFTAVGELAEANAMRMHATMLGALPPVLactobacillus_helveticus/1-320     GMK-EAQSSPFFNPWLERNETELNQMIAAIKNDDFTALGELAELNANEMHAINLTAQPEFLactobacillus_acidophilus/1-320    GMK-DAQSSPFFSTWTNRNQLELDEMIKAIKQNDFTALGSLAELNANEMHAINLTAQPEFStreptococcus_pyogenes/1-314       GMKLCRDTSTTFDQWVEQSAIDYQHMLTYLKTNNFEKVGQLTEANALAMHATTKTANPPFBorrelia_garinii/1-312             AMNICKHHEFYYDAWITSSKKIFKDALYFFLKKDFIRFGANVVKSYQNMFALMFAS--SIBorrelia_spielmanii_/1-312         AMNICKHHGFYYDAWIASSKKIFKDALYFFLKKDFIHFGANVVKSYQNMFALMFAS--SI|gb|EGF88987.1|/1-303              AMEQCVQTSTTFASWVEKAKKDFVLMKEALKEADFEKIGEITESNALAMHETTTTSSPSF|ref|YP_804426.1|/1-327            GMQHTASTSPFYPAWVEATNQAIPEMISAVQNNDFTKIGELAEHSAAMMHATTLSSKPAF|ref|NP_691147.1|/1-324            GMRRTVETSPFYDGWLKQTPKDLEEIKTAIHDKDFEKTGSIAEANCMRMHATTLGANPPF|gb|ADQ43374.1|/1-351              AMRRTVDTSPLYEPWALSSKGDLAEMRRALGRGDLEAVGEIAERNALGMHATMLAARPAV|ref|ZP_07819449.1|/1-328          GMEHTMQSSPFYALWPEEVAKDLSAMEDAIKDRNIDQIGIIAEHNAMKMHATMIASNPSF|gb|AAO90151.1|/1-503              GMRRTVTTSPLYSAWPEKANRDLTQLKKAIAKKDFNLLGRTAESNALAMHATMLAAWPPL|ref|ZP_06751493.1|/1-340          GMKHTAETSAFYPLWRQASERDLQRVLEGIANRDVDLIGQAMEANAMKFHATMFSADPPL|ref|ZP_07086643.1|/1-352          GHG-LMKTNPYAERRFQEARENFVPMKEILKNGDMERFIKLVEHEALTLHAMMMMSDPAFSepidermis/1-332                   GMSLTRDTSRFYQYWLDHVDEDLNEAKEAVKNQDFQRLGEVIEANGLRMHATNLGAQPPFSaureus/1-327                      TYLVQESYDVMALVHECREA-GYPCYFTMDAGPNVKILVEKKNKQQIIDKLLTQ------Staphylococcus_warneri_/1-327      TYLVQESYDAMAIVHECRKM-GVPCYFTMDAGPNVKVLVEKKNKQLVIDQFLKH------Staphylococcus_carnosus/1-331      TYLVSDSYEAMSLVHDCREA-GIPAYFTMDAGPNVKVLVQKKDQQAVIDKLTSY------Enterococcus_faecalis/1-331        TYWSPGSLQAMALVRQARAK-GIPCYFTMDAGPNVKVLVEKKNLEALKTFLSEH------Lactobacillus_iners/1-325          SYFQPTTITIMNLVRNLRKN-GIECYYTIDAGPNVKILCQDKNVEDICKAIHNT------Listeria_monocytogenes_/1-339      TYFQPQSLEIMDAVRELREN-GIPAYFTMDAGPNVKVICERANENIVAEKLSGL------Lactobacillus_johnsonii/1-321      TYFEPETIKIIKLVEDLRKN-GIECYYTIDAGPNVKIICTLRNRKDIISAVQKT------Lactobacillus_reuteri_/1-323       TYFNGETLTIIKAVEDLRNQ-GINCYYTMDAGPNVKVIYDRENRSKIVEKLSNI------Lactobacillus_gasseri/1-321        TYFEPETIKAIKLVEDLRKQ-GIECYYTIDAGPNVKILCTLRNRKEIISAVQKS------Corynebacterium_amycolatum/1-325   RYWNPDSVAALDLVATLRDE-GTECYATMDAGPNVKVLCRSGDAETIADRFRAE------Lactobacillus_helveticus/1-320     TYFEPNTIRAIKLVEDLRKE-GIECYYTIDAGPNIKILCRLRNSKEIIERFKSV------Lactobacillus_acidophilus/1-320    TYFMPETIRAIKLVEDLRTK-GIECYYTIDAGPNIKVLCQLKNRKEIIEHFESV------Streptococcus_pyogenes/1-314       SYLTKESYQAMEAVKELRQE-GFACYFTMDAGPNVKVLCLEKDLAQLAERLGKN------Borrelia_garinii/1-312             FYFKSSTIDLIKYAANLRNE-GIFIFETMDAGPQVKFLCLEKNLNTILKRLKQN------Borrelia_spielmanii_/1-312         FYFKSSTIDLIKYAANLRNE-GIFVFETMDAGPQVKFICLEKNLNTILKGLKKN------|gb|EGF88987.1|/1-303              TFLTEESHRAMDIVKQLRSQ-GYKCYFTMDAGPNVKVLYLKEDQEKLHEEISKL------|ref|YP_804426.1|/1-327            TYFAPETIQAIKLVEQLRES-GIECYYTIDAGPNVKVLCQSKNITRVKRFFASY------|ref|NP_691147.1|/1-324            TYWQDTTMRVMQNVQQMREE-GIPAYFTIDAGPNVKVLYLPKDESKVKQRLEQI------|gb|ADQ43374.1|/1-351              RYMSPASLTVLDSVLQLRRD-GVAAYATMDAGPNVKVLCRAADADRVADTVRAA------|ref|ZP_07819449.1|/1-328          TYWQAQSLLAMERVRQLRQA-GYSAYFTMDAGPNVKVICPYSQVEAIRQALLDD------|gb|AAO90151.1|/1-503              LYSSPETITVMQKIWSLREA-GTEIYFTQDAGPNIKLLFLESNKEKIKQSFPEIEIISPF|ref|ZP_06751493.1|/1-340          TYLTARSWEVIEFVWAMRQE-GVSAYFTMDAGPNVKILCRKSQMEEISRRLRER------|ref|ZP_07086643.1|/1-352          ILMKTGTLEVINKIWDFRRETGSPLFFTLDAGANVHLLFPNNGSEEQIKAF---------Sepidermis/1-332                   TYLVQESYDAMAIVEQCRKA-NLPCYFTMDAGPNVKVLVEKKNKQAVMEQFLKV------Saureus/1-327                      ------------------------------------------------------------Staphylococcus_warneri_/1-327      ------------------------------------------------------------Staphylococcus_carnosus/1-331      ------------------------------------------------------------Enterococcus_faecalis/1-331        ------------------------------------------------------------Lactobacillus_iners/1-325          ------------------------------------------------------------Listeria_monocytogenes_/1-339      ------------------------------------------------------------Lactobacillus_johnsonii/1-321      ------------------------------------------------------------Lactobacillus_reuteri_/1-323       ------------------------------------------------------------Lactobacillus_gasseri/1-321        ------------------------------------------------------------Corynebacterium_amycolatum/1-325   ------------------------------------------------------------Lactobacillus_helveticus/1-320     ------------------------------------------------------------Lactobacillus_acidophilus/1-320    ------------------------------------------------------------Streptococcus_pyogenes/1-314       ------------------------------------------------------------Borrelia_garinii/1-312             ------------------------------------------------------------Borrelia_spielmanii_/1-312         ------------------------------------------------------------|gb|EGF88987.1|/1-303              ------------------------------------------------------------|ref|YP_804426.1|/1-327            ------------------------------------------------------------|ref|NP_691147.1|/1-324            ------------------------------------------------------------|gb|ADQ43374.1|/1-351              ------------------------------------------------------------|ref|ZP_07819449.1|/1-328          ------------------------------------------------------------|gb|AAO90151.1|/1-503              KTSREQRVVLVDENDRRLGIEEKIKAHREGKLHRAFSVFIFSRKNNEWQLLLQQRHPEKY|ref|ZP_06751493.1|/1-340          ------------------------------------------------------------|ref|ZP_07086643.1|/1-352          ------------------------------------------------------------Sepidermis/1-332                   ------------------------------------------------------------Saureus/1-327                      ------------------------------------------------------------Staphylococcus_warneri_/1-327      ------------------------------------------------------------Staphylococcus_carnosus/1-331      ------------------------------------------------------------Enterococcus_faecalis/1-331        ------------------------------------------------------------Lactobacillus_iners/1-325          ------------------------------------------------------------Listeria_monocytogenes_/1-339      ------------------------------------------------------------Lactobacillus_johnsonii/1-321      ------------------------------------------------------------Lactobacillus_reuteri_/1-323       ------------------------------------------------------------Lactobacillus_gasseri/1-321        ------------------------------------------------------------Corynebacterium_amycolatum/1-325   ------------------------------------------------------------Lactobacillus_helveticus/1-320     ------------------------------------------------------------Lactobacillus_acidophilus/1-320    ------------------------------------------------------------Streptococcus_pyogenes/1-314       ------------------------------------------------------------Borrelia_garinii/1-312             ------------------------------------------------------------Borrelia_spielmanii_/1-312         ------------------------------------------------------------|gb|EGF88987.1|/1-303              ------------------------------------------------------------|ref|YP_804426.1|/1-327            ------------------------------------------------------------|ref|NP_691147.1|/1-324            ------------------------------------------------------------|gb|ADQ43374.1|/1-351              ------------------------------------------------------------|ref|ZP_07819449.1|/1-328          ------------------------------------------------------------|gb|AAO90151.1|/1-503              HSGGLWTNTCCSHPRPDEDIVTAGERRLFEETGLKIPLKRVGEFHYTATVGNQLIENEYD|ref|ZP_06751493.1|/1-340          ------------------------------------------------------------|ref|ZP_07086643.1|/1-352          ------------------------------------------------------------Sepidermis/1-332                   ------------------------------------------------------------Saureus/1-327                      ------FDNNQIIDSDIIATGIEIIE------------------------------Staphylococcus_warneri_/1-327      ------FEASQIIASDIIATGVEIIK------------------------------Staphylococcus_carnosus/1-331      ------FDPEQIIASN-IGTGVEILNEEDAL-------------------------Enterococcus_faecalis/1-331        ------FSKEQLVPAF-AGPGIELFETKGMDK------------------------Lactobacillus_iners/1-325          ------LDSVKIIKSK-FGPGVQIINCDD---------------------------Listeria_monocytogenes_/1-339      --------AKNVLICH-AGKEASVVSDEK---------------------------Lactobacillus_johnsonii/1-321      ------LTNVKIVVAS-FGPGVTLL-------------------------------Lactobacillus_reuteri_/1-323       ------VGPERLVVSQ-PGPGIKIWNE-----------------------------Lactobacillus_gasseri/1-321        ------LTNVKIVVAS-FGPGVTLL-------------------------------Corynebacterium_amycolatum/1-325   ------FEDIDVLVSG-SGPGAYLV-------------------------------Lactobacillus_helveticus/1-320     ------FNNVNIVIAN-FGPGITYLD------------------------------Lactobacillus_acidophilus/1-320    ------FNNVNIVSAS-FGPGVIYLD------------------------------Streptococcus_pyogenes/1-314       ---------YRIIVSK-TKDLPDV--------------------------------Borrelia_garinii/1-312             ------FTDINFIVSK-VGCGLEWI-------------------------------Borrelia_spielmanii_/1-312         ------FTGINFIVSK-VGCDLEWI-------------------------------|gb|EGF88987.1|/1-303              ------WNKKIILCME----------------------------------------|ref|YP_804426.1|/1-327            ------FDQDQLVVAK-PGSGIKFTKN-----------------------------|ref|NP_691147.1|/1-324            ------MGVEDVIVSK-PGKGISYL-------------------------------|gb|ADQ43374.1|/1-351              ------AQGGAVHIAR-PGPGARLLTEDGR--------------------------|ref|ZP_07819449.1|/1-328          ------FAEDHLVISR-PGPAPYAV-------------------------------|gb|AAO90151.1|/1-503              HVLIGFTDADAIDFNKKEISAVRWIRVSELKNELKENPSHFTPWFMQALEIAIKPL|ref|ZP_06751493.1|/1-340          ------FPQAALFQST-SGPGPISLDFARWSQLYGEPSV-----------------|ref|ZP_07086643.1|/1-352          ------IEAELLQHTQKNGVVKDVMRF-----------------------------Sepidermis/1-332                   ------FDESKIIASDIISSGVEIIK------------------------------
