# Supplementary material for: Discovery of a metabolic alternative to the classical mevalonate pathway
Source: eLife. 2013 Dec 10;2:e00672. doi: 10.7554/eLife.00672 (PMC3857490; doi:10.7554/eLife.00672)
Supplement: Table 4—source data 3. — Amino acid sequence alignments of Chloroflexi MDDs. DOI: http://dx.doi.org/10.7554/eLife.00672.020 [file elife00672s009.rtf]

Table 4 - Source Data 3. Amino acid sequence alignments of Chloroflexi MDDs.gi|156740939_Roseiflexus_castenholzii/1-402 MYHRPLERMPGLATAIPAPYADLVERMATADADLRAALRAHGLTWEEYPDLTGAARERGAgi|148658371_Roseiflexus_sp.RS-1/1-376      --------------------------MASADAELRAALRAHGLRWEAYPDLTGAARVRGVgi|309791678_Otrichoides/1-389              ----------MT-TTLPAGFADMLEPMRIGHERILAALAQHGVEYEAYPERIPDPRPTGRgi|163846202_Caurantiacus/1-390             ----------MNQSTIPHYVSDLVVPMRTAHEQILAELQQHNLLPPP-PPPLPPARAQGMgi|219850237_Caggregans/1-389               ----------MNESSIPQGFADLVAPMRTAHERIIADLRRHHIELPP-PPHLPPACRQGIgi|222523952_Csp.Y400/1-374                 --------------------------MRTAHEQILAELQQHNLLPPP-PPPLPPARAQGMgi|320161393_Athermophila/1-326             ---------------------------------------------------------MGQgi|159898136_Haurantiacus/1-334             -----------------------------------------------------MKQLSHAgi|156740939_Roseiflexus_castenholzii/1-402 AAALAYPMQGVLKYHGLSDWDYRIAFLPSVSLCNDAGHTLTLVEFDPDLATDCATI---Ngi|148658371_Roseiflexus_sp.RS-1/1-376      AAALAYPMQGVLKYHGLSDWKYRIAFLPSISLCNDAGHTLTLVEFDPDLPDDSATI---Ngi|309791678_Otrichoides/1-389              AAARAFPMQGVLKYHGLSDWHYRIAYLPSISLNNSAAHTTTCVEFDPQLEADCAEI---Ggi|163846202_Caurantiacus/1-390             AAARAYPMQGVLKYHGLSDWTQRIAFLPSISLNNAAAHTTTLVEFDPALPADTAII---Ggi|219850237_Caggregans/1-389               AAARAFPMQGVLKYHGLSDWVQRIAFLPSISINNAAAHTTTLVEFDPALPADSAVI---Ggi|222523952_Csp.Y400/1-374                 AAARAYPMQGVLKYHGLSDWTQRIAFLPSISLNNAAAHTTTLVEFDPALPADTAII---Ggi|320161393_Athermophila/1-326             ATAIAHPNIAFIKYWGNRDAVLRIPENGSISMNLAELTVKTTVIFEKHSREDTLIL---Ngi|159898136_Haurantiacus/1-334             ATAVACANIAFIKYWGQHDSQLTLPTNGSISMNLDGCLTETTVQCLPEAVDDSVWLALSGgi|156740939_Roseiflexus_castenholzii/1-402 G--HVARGRELERVRQSLDAIRAASGATVRARVMSRNVTRGTRMGKGLGSSAAASAALALgi|148658371_Roseiflexus_sp.RS-1/1-376      G--QPARGRELERVQQSLDAIRAVSGATVHARVTSRNVTRGTRFGKGLGSSASASAALALgi|309791678_Otrichoides/1-389              G--VNATGRDLDRVVQTLNAVRELSGCQTRARVTSRNILQSRVAGKGLGTSASASAALAAgi|163846202_Caurantiacus/1-390             G--IPAQGRELERVVTVLDAVRSLAGISSRARVISRNVLRTKVAGKGLGTSASAAAALACgi|219850237_Caggregans/1-389               G--VPAHGRELERIVHVLDTVRSLAGITSHARVVSRNIVRTRTTGKGLGTSASAAAALACgi|222523952_Csp.Y400/1-374                 G--IPAQGRELERVVTVLDAVRSLAGISSRARVISRNVLRTKVAGKGLGTSASAAAALACgi|320161393_Athermophila/1-326             G--ALADEPALKRVSHFLDRVREFAGISWHAHVISENNFPT---GAGIASSAAAFAALALgi|159898136_Haurantiacus/1-334             GEEVQAKGRQFERVIQQIERLRQLAGVTERVEVRSRNNFPS---DAGIASSAAAFAALTRgi|156740939_Roseiflexus_castenholzii/1-402 AAIAALYGDEAAANRRLVSCMARL-LAGSGCRSAAGGCSIWFSSPGMPHEDSFAVRLDDAgi|148658371_Roseiflexus_sp.RS-1/1-376      AAIAALYGEEAASNRRLVSCMARL-LAGSGCRSAAGGCSIWLSYPGIAHEESFAVRLDDAgi|309791678_Otrichoides/1-389              AALAALYGPELASNRRFLSCFARL-LAGSGCRSAAGGLALWLSYPSLSHADSFAVRLDDAgi|163846202_Caurantiacus/1-390             AAISALFGPELAGHTRFLSTLARL-LAGSGCRSTAGGLALWLSYPGISPTESYAVRLDQHgi|219850237_Caggregans/1-389               AAVGAIFGPELAGHTRFLSTLARR-LAGSGCRSAAGGLALWLSYPGIPPDESFAVRLDQDgi|222523952_Csp.Y400/1-374                 AAISALFGPELAGHTRFLSTLARL-LAGSGCRSTAGGLALWLSYPGISPTESYAVRLDQHgi|320161393_Athermophila/1-326             AATSAI---GLHLSERDLSRLARK-GSGSACRSIPGGFVEW--IPGETDEDSYAVSIAPPgi|159898136_Haurantiacus/1-334             AAASAF---RLELDEAELSRLTRLSGSGSACRSIPAGFVEW--YNDGTHAGSYAAQIAPPgi|156740939_Roseiflexus_castenholzii/1-402 GQLDDVRLITVPLDSRIGLKTEQAHLDAPGSALFRCWMLSRRDEALACIAAARTGDWRTLgi|148658371_Roseiflexus_sp.RS-1/1-376      GQLDDVRLITVPIDSRIGLKTEQAHMDAPASALFRCWMLNRRDEALACIAAARAGDWRTLgi|309791678_Otrichoides/1-389              GQMDHVSLVTVPIDSSIGLKTESAHHDAPESSLFRSWMLSRADEIIECLTAIRAGDWRTVgi|163846202_Caurantiacus/1-390             NELADVALITVPIDSRIGLKTEQAHHDAPNSIFFRSWMLARGDEVRECISAVRRGDWQTIgi|219850237_Caggregans/1-389               HELDDLALITVPIDSRIGLKTEQAHHDAPQSIFFRAWMLARGDEVRECIAAARRGDWQTIgi|222523952_Csp.Y400/1-374                 NELADVALITVPIDSRIGLKTEQAHHDAPNSIFFRSWMLARGDEVRECISAVRRGDWQTIgi|320161393_Athermophila/1-326             EHWALTDCIAILSTQHKPIGSTQGHALASTSPLQPARVADTPRRLEIVRRAILERDFLSLgi|159898136_Haurantiacus/1-334             EHWNLVDIVAVISTEAKHVASTSGHSVATTSPYFSVRLEGIEQRLADVRQGILERDIERLgi|156740939_Roseiflexus_castenholzii/1-402 GQWAELDSMRLHGITMSGSLENKLIGWEPENIVLFRMCNDLRSS-GVPVYCSTDTGPTAVgi|148658371_Roseiflexus_sp.RS-1/1-376      GQWAELDSMRLHGITMSGSLENKLIGWEPENIALFRMCNDLRSG-GVPVYCSTDTGPTAVgi|309791678_Otrichoides/1-389              GQLAEMDSMRLHGVTMSGSRENKLVGWEPENITLFRMCNDLRER-GVPVYASTDTGPTVVgi|163846202_Caurantiacus/1-390             GQLAELDSMRLHGVTMSGSREQRIIGWEPENITLFRLCNDLRAR-GVPVYASTDTGPTVVgi|219850237_Caggregans/1-389               GQLAELDSMRLHGVTMSGSREQKIIGWEPENITLFRLCNDLRAR-GVPVYASTDTGPTVVgi|222523952_Csp.Y400/1-374                 GQLAELDSMRLHGVTMSGSREQRIIGWEPENITLFRLCNDLRAR-GVPVYASTDTGPTVVgi|320161393_Athermophila/1-326             AEMIEHDSNLMHAVMMTS--TPPLFYWEPVSLVIMKSVREWRES-GLPCAYTLDAGPNVHgi|159898136_Haurantiacus/1-334             GRASEADAMSMHVIAMTA--QPSTMYWLPGTLAVMQAVQRWRAQDNLQSYWTIDAGPNVHgi|156740939_Roseiflexus_castenholzii/1-402 FITHRDYEDAVVSAIEGLGLSLEAIRGRVAGPARLVDIAWAGGELGVAD-gi|148658371_Roseiflexus_sp.RS-1/1-376      FITHRDYEEAVVAAIEATGLGLETIRGRIAGPARLVDVAWAQGALGVEG-gi|309791678_Otrichoides/1-389              FITHRDHEDALVEAINGLGLGLETIRGKIAGPASLIDPDEALAEI--RG-gi|163846202_Caurantiacus/1-390             FITHRDHAPTVVAAIHDLGLNLETVVAPIGGPAHVIPVEEALSEL--EGVgi|219850237_Caggregans/1-389               FITRRDFAPIVTDAIHHSGLNVETVIAPIGGPAHLIPVEEALAEL--QS-gi|222523952_Csp.Y400/1-374                 FITHRDHAPTVVAAIHDLGLNLETVVAPIGGPAHVIPVEEALSEL--EGVgi|320161393_Athermophila/1-326             VICPSEYAEEVIFRLTSIPGVQTVLKASAGDSAKLIE----------QSLgi|159898136_Haurantiacus/1-334             VICEAKDAPEVEARLCELDAVQWTIVNGAGPEARLVG-------------
