# Supplementary material for: Discovery of a metabolic alternative to the classical mevalonate pathway
Source: eLife. 2013 Dec 10;2:e00672. doi: 10.7554/eLife.00672 (PMC3857490; doi:10.7554/eLife.00672)
Supplement: Table 4—source data 4. — Amino acid sequence alignments of eukaryotic MDDs. DOI: http://dx.doi.org/10.7554/eLife.00672.021 [file elife00672s010.rtf]

Table 4 - Source Data 4. Amino acid sequence alignments of eukaryotic MDDs.Atha/1-392                         MATEKWV------------FMVTAQTPTNIAVIKY--------W------------GKRDgi|327287778_Acaronlinensis/1-406  MAEDHPL------------SMVTCTAPINIAVIKY--------W------------GKRDgi|260794527_Bfloridae/1-406       MENGRANMKPVTGI-----SQVTCTAPVNIAVIKY--------W------------GKRDEcab/1-376                         MASEKPL------------VAVTCTAPVNIAVIKY--------W------------GKRDCfam/1-376                         MASEKPL------------LAVTCTAPVNIAVIKY--------W------------GKRDMVD_Hs/1-376                       MASEKPL------------AAVTCTAPVNIAVIKY--------W------------GKRDMmus/1-376                         MASEKPQD-----------LMVTCTAPVNIAVIKY--------W------------GKRDMdom/1-376                         MAAEKPL------------VSVTCTAPTNIAVIKY--------W------------GKRDOana/1-376                         MIPENFKGVQKIARLPKVHSLAHCGQGLDLPTLLY--------W------------GKRDMVD_Acar/1-374                     MAEDHPL------------SMVTCTAPINIAVIKY--------W------------GKRDXtro/1-376                         M------------------KKVTCTAPVNIAVIKY--------W------------GKRNGgal/1-366                         MPCQAVLCCAKWCQAIPS-GAMPCQAVLCCAKQRSAAHSGTVPWQALPYLSTSPAGGKRDSalmo/1-376                        MPEVSGKKL----------TMVTCSAPVNIAVIKY--------W------------GKRDTnig/1-376                         EKP----------------NILTCTAPVNIAVIKY--------W------------GKRNDrer/1-376                         MSENILQDL----------EMVTCTAPVNIAVIKY--------W------------GKRDDC_Spur/1-371                      MDIS---------------KTVTCKAPINIAVIKY--------W------------GKRDSaccoglossus_kowalevskii/1-378     MTANNDNTTWSG-------RSVTCTAPINIAVIKY--------W------------GKRDTrichoplax_adhaerens/1-374         MADR---------------KQATCIAPVNIAVVKY--------W------------GKRDAaeg/1-372                         M------------------ISVTCIAPVNIAVIKY--------W------------GKRDDmel/1-374                         M------------------FSVTCVAPVNIALIKY--------W------------GKRHTcas/1-372                         M------------------KTVTGIAPVNIAVVKY--------W------------GKRDArabidopsis_lyrata/1-392           MATEKWV------------FMVTAKTPTNIAVIKY--------W------------GKRDAtha2/1-385                        MAEEKWV------------VMVTAQTPTNIAVIKY--------W------------GKRDArabidopsis_lyrata2/1-385          MAEEKWV------------MMVTAQTPTNIAVIKY--------W------------GKRDPtri/1-389                         MAEKTWV------------RMVTAQTPTNIAVIKY--------W------------GKRDRicinus_communis/1-389             MAERWV-------------RMVTAQTPTNIAVIKY--------W------------GKRDVvin/1-392                         MAADSSQKWI---------LMTTAQTPTNIAVIKY--------W------------GKRDZea_mays/1-389                     MAAAEGQWV----------LMATGRTPTNIAVIKY--------W------------GKRDPpat/1-392                         MGNEEETWV----------VMKTARAPSNIAVIKY--------W------------GKRDSmoe/1-388                         MGTDGSAWI----------VSRTARAPSNIAVIKY--------W------------GKRDSalpingoeca_sp./1-378              MDAKRAKTGV---------VRATTTAPVNIAVIKY--------W------------GKRDMonosiga_brevicollis/1-311         MAQPEEKRVKLAV------ARHTATAPVNIAVIKY--------W------------GKRDCapsaspora_owczarzaki/1-378        M------------------LTNQCET----SILTL--------W----TDDDDDQGGKRDLbic/1-373                         MSTV---------------YQATASAPVNIAVIKY--------W------------GKRDLbic2/1-373                        MSTV---------------YQATASAPVNIAVIKY--------W------------GKRDCoprinopsis_cinerea/1-387          MSEPI--------------YEATASAPVNIAVIKY--------W------------GKRDSchizophyllum_commune/1-382        MST----------------YQATASAPVNIACIKY--------W------------GKRDCryptococcus_neoformans/1-375      MV-----------------YEATASAPVNIACIKY--------W------------GKRDKluyveromyces_lactis/1-380         MSI----------------YSASTTAPVNIATLKY--------W------------GKRDScer/1-379                         MTV----------------YTASVTAPVNIATLKY--------W------------GKRDAory/1-379                         MAAPSDSTV----------FRATTTAPVNIAVIKY--------W------------GKRDYlip/1-370                         MI-----------------HQASTTAPVNIATLKY--------W------------GKRDSchizosaccharomyces_pombe/1-368    MDKKV--------------YQCTVSAPVNIAVIKY--------W------------GKRDThalassiosira_pseudonana/1-335     I------------------HTATVSAPTNIACIKY--------W------------GKASPhaeodactylum_tricornutum/1-372    MTTPATHPI----------FLATVSAPTNIAVVKY--------W------------GKADEctocarpus_siliculosus/1-395       MTPAIDSR-----------LCVTCSAPTNIAVIKY--------W------------GKDSPhytophthora_infestans/1-384       M------------------KVATCSAPTNIAVIKY--------W------------GKDDDictyostelium_purpureum/1-377      MVL----------------ASITCSAPVNIAVIKY--------W------------GKRDDictyostelium_discoideum/1-377     MVL----------------ASVTCTAPVNIAVIKY--------W------------GKRDPolysphondylium_pallidum/1-378     ML-----------------SSITCTAPVNIATIKY--------W------------GKRDNaegleria_gruberi/1-386            MSFTS--------------PKFTCTSPVNIAVIKY--------W------------GKENNvec/1-377                         MADDLETSAKRQKTEQI--FRVTVKAPINIAVIKY--------W------------GKRDLeishmania_major/1-372             MSAP---------------IRVTVEAPINIAFIKY--------W------------GKREgi|149241991|pdb|2HKE|A/1-372      MSD----------------QCVTVEAPINIAFIKY--------W------------GKREEncephalitozoon_intestinalis/1-302 MKDS---------------SLGYGRSHPNIAVIKY--------W------------GKVDEncephalitozoon_cuniculi/1-303     MKKP---------------LSGYGSSHPNIAVIKY--------W------------GKADTthe/1-384                         MITVKGDILK---------VKTTYETSPNIALVKY--------W------------GKFDParamecium_tetraurelia/1-355       MFTQLNKKLT---------LSSSFKTAPNIGLIKY--------W------------GKWNTrichomonas_vaginalis/1-341        MSTSNPTHT----------SHAVCTSHPNIALVKY--------W------------GKENSchistosoma_mansoni/1-387          -----------------------VTCPVNIALLKY--------W------------GKGDSchistosoma_japonicum/1-239        ------------------------------------------------------------Bmal/1-393                         MSSNDGSSDRV--------REVKVIAPINIALVKY--------W------------GKRNTrichinella_spiralis/1-366         MSLPHG-------------HSVHVRAPVNLALVKY--------W------------GKRDCele/1-365                         MSQDDGAAE----------AEATVRVPMNIALVKY--------W------------GKRDAtha/1-392                         --EVRILPVNDSISVTLDPDHLCTVTTVAVSPAF-DRDRMWLNGKEIS--L-SGSRYQNCgi|327287778_Acaronlinensis/1-406  --EKLILPINSSLSVTLHQDQLKTTTTAAISRDF-TEDRLWLNGKESD--I-GHPRLQSCgi|260794527_Bfloridae/1-406       --EQLVLPINPSLSVTLSQDQLCARTTVAASADF-KRDRVWLNGQEQS--V-DAPRLQKCEcab/1-376                         --EELILPINSSLSVTLHQDQLKTTTTAAISKDF-TEDRIWLNGREED--V-GQPRLQACCfam/1-376                         --EDLVLPINSSLSVTLHQDQLKTTTTAAVSKDF-TEDRIWLNGREED--V-EQPRLQACMVD_Hs/1-376                       --EELVLPINSSLSVTLHQDQLKTTTTAVISKDF-TEDRIWLNGREED--V-GQPRLQACMmus/1-376                         --EALILPINSSLSVTLHQDQLKTTTTVAISKDF-TEDRIWLNGREED--V-GQPRLQACMdom/1-376                         --EKLILPINSSLSVTLHQNQLKTTTTAAISRDF-KEDRIWLNGKEED--V-GHHRLQSCOana/1-376                         --EELILPINSSLSVTLHQDQLKTTTTAAISRDF-KEDRIWLNSKEED--V-GHPRLQTCMVD_Acar/1-374                     --EKLILPINSSLSVTLHQDQLKTTTTAAISRDF-TEDRLWLNGKESD--I-GHPRLQSCXtro/1-376                         --EELILPINSSLSVTLHQDQLKTTTSAAASREF-TEDRIWLNGKEEN--I-SHPRLQSCGgal/1-366                         --TDLILPINSSLSVTLHQDQLRTTTTAAACRDF-TEDRLWLNGEEVD--A-AQPRLQACSalmo/1-376                        --EELILPINSSLSVTLHQDQLKTTTTVACSRSF-QEDRIWLNGKEED--I-TQPRLQSCTnig/1-376                         --EELILPINSSLSVTLHQDQLKTTTTVVTSKSF-EEDRIWLNGKEED--I-SHPRLQSCDrer/1-376                         --EDLILPVNASLSVTLHQDHLRTTTTIACSRSF-HKDCIWLNGKEQD--I-SHPRLQSCDC_Spur/1-371                      --EKLILPVNSSLSATLHIDQLCTTTSIAGSKHF-PEDRLWLNGKEES--L-ENPRVKTCSaccoglossus_kowalevskii/1-378     --EQLILPTNSSLSASLDQDHLKSTTTASISKEF-KRDRLWLNGKEES--I-ENPRIQNCTrichoplax_adhaerens/1-374         --ENLILPINSSLSGTLSTDQMCAKTTIAISKSF-QRDRLWINGKEQD---ATGKRLQNCAaeg/1-372                         --EDLILPLNDSVSATLSTDHLCTKTTITTCESF-TENKIILNGKEES--F-DNPRLLRCDmel/1-374                         --EELILPVNDSISMTLSTDELCAKTTVTASESF-ETNRMWLNGEEVP--FEESSRLQRCTcas/1-372                         --EDLILPINDSLSCTLSTDFMCAKTTIMASPTF-PTHRFWLNGKESD--F-NNERLNNCArabidopsis_lyrata/1-392           --EVRILPVNDSISVTLDPDHLCTVTTVAVSPAF-DRDRMWLNGKEIS--L-SGSRYQNCAtha2/1-385                        --EVRILPINDSISVTLDPDHLCTLTTVAVSPSF-DRDRMWLNGKEIS--L-SGSRYQNCArabidopsis_lyrata2/1-385          --EVRILPINDSISVTLDPDHLCTLTTVSVSPSF-DRDRMWLNGKEIS--L-SGSRYQNCPtri/1-389                         --ETLILPVNDSISVTLDPAHLCTTTTVAVSPSF-DQDRMWLNGKEIS--L-SGGRYQNCRicinus_communis/1-389             --ETLILPVNDSISVTLDPAHLCTTTTVAVSPAF-DQDRMWLNGKEIS--L-SGGRYQNCVvin/1-392                         --EALILAVNDSISVTLDPQHLCTTTTVAVSPMF-QSDRMWLNGKEIS--L-SGGRYQNCZea_mays/1-389                     --EALILPINDSISVTLDPDHLSATTTVAVSPSF-PSDRMWLNGKEIS--L-LGGRFQSCPpat/1-392                         --EKLILPINSSISVTLDPEHLSATTTVAASPAF-EKDRLWLNGKEVS--V-EGVRYKNCSmoe/1-388                         --EDLILPLNSSISVTLDPNDLSATTTVSTSPDF-DADRLWLNDKEVS--L-SSHRYVSCSalpingoeca_sp./1-378              --TKLLLPINSSLSGTLDQEQLHARTTVAASSSF-EADEIWLNGKQED--I-SNQRLQNVMonosiga_brevicollis/1-311         --TKLLLPINDSLSGTLSQDEMHARTTVAASESY-AEDTLWLNNEQTD--I-SNPRVQNVCapsaspora_owczarzaki/1-378        --EELILPINSSLSGTLSQDQLHARTSVMARADF-ASDAIWLNGKQES--I-ENPRLQNCLbic/1-373                         --TKLILPTNSSLSVTLDQDHLRSTTTSRADPSF-VKDTLWLNGKEDE--IKPGGRLATCLbic2/1-373                        --TKLILPTNSSLSVTLDQDHLRSTTTSRADPSF-VKDTLWLNGKEDE--IKPGGRLATCCoprinopsis_cinerea/1-387          --TSLILPTNSSLSVTLSQDHLRSTTTSRASSSF-DKDRLWLNGQEDV--IKPGSRLETCSchizophyllum_commune/1-382        --TKLILPTNSSLSVTLDQDYLKSTTTSRADPSF-EKDQLWLNGTEDE--IKPGSRLETCCryptococcus_neoformans/1-375      --TRLILPTNSSLSVTLDQDHLRSTTTSRADASFEAGDKLWLNGKEEV--IKEGGRLAVCKluyveromyces_lactis/1-380         --KVLNLPTNSSISVTLSQEDLRTLTTATTSPDF-AKDQLWLNGKEES--L-ASERTQHCScer/1-379                         --TKLNLPTNSSISVTLSQDDLRTLTSAATAPEF-ERDTLWLNGEPHS--I-DNERTQNCAory/1-379                         --ATLNLPTNSSLSVTLSQRSLRTLTTASCSAKYPTADELILNGKPQD--IQSSKRTLACYlip/1-370                         --PALNLPTNNSISVTLSQDDLRTLTTASCSPDF-TQDELWLNGKQED--V-SGKRLVACSchizosaccharomyces_pombe/1-368    --VALNLPTNSSISVTLSQDDLRTVTTASCSEKF-ENDTLWLNGNAEE--IFANKRLRVCThalassiosira_pseudonana/1-335     --SKYNTPINSSLSLTLDQSDLRAVTTAAASTSF-TKDRLWLNGSEEANAF-TSKRFRACPhaeodactylum_tricornutum/1-372    --EHYNTPINSSCSVTLHQDDLRAVTTVAVSKDF-VQDRLWLNGVEVP-HAATSRRFRACEctocarpus_siliculosus/1-395       --VALNTPINSSASVTLSQDDLRAITTVAASKDF-EKDQLWLNGTEED--VSKNKRFQAVPhytophthora_infestans/1-384       --VALNTPINSSVSVTLHQNQLRTTTSVAGGSEL-QSTRLWLNGQEQP----INKRVTVVDictyostelium_purpureum/1-377      --EKLILPLNSSLSGTLHQDDLKTTTTIVASEDY-TEDAIWLNGKKED--I-NATRYQNVDictyostelium_discoideum/1-377     --ENIILPLNSSLSGTLHQDDLKTTTTIVASEDY-TEDELYLNGKKED--I-NAVRYQNVPolysphondylium_pallidum/1-378     --EKLILPLNSSLSGTLHQDDLKTTTTAVASENF-TEDAIWLNGKKED--I-NTTRYQNVNaegleria_gruberi/1-386            --EAEHIPLNSSLSATLNQDDLKTTTTVQASTEF-PCDELILNGKKED--VQGSKRIQRVNvec/1-377                         --EELILPLNSSLSATINLDELCTTTTVVARRDN-PQDSLWINKREQP--IAESPRIQKCLeishmania_major/1-372             GGETLILPTNDSFSITLSTKPFRSKTSVELRSDA-SEDELWLNGKKSN--IQETPRIQSVgi|149241991|pdb|2HKE|A/1-372      GGETLILPTNDSFSITLSASPFRSKTSVELRDDI-ETDTLRLNGTEVD--VGKTPRVQSMEncephalitozoon_intestinalis/1-302 --TINNTPSNSSVSFPLT--NFQTETTVEYS----NSDRFYLNGEMLS----FGKKMSQVEncephalitozoon_cuniculi/1-303     --TINNMPSSRSISFPLT--NFLTETVVEHSL---EEDRFYLNGKMLP----IGEKMGRATthe/1-384                         --EEYILPLNSSTGITLSTEDLQTRTTITLTNKY-KDIKFLLNGQPHP----VSGRLKKIParamecium_tetraurelia/1-355       --EREIIPLNTNIGVTLNPKDIFTTTTLTLNPET-DKNQLLINGKDFH----ISNRIERLTrichomonas_vaginalis/1-341        --IPEITPIHGSLSVTLN---FGVTTTKAEYSSD-DVDHFYLNNKEAE----ITSRLKTASchistosoma_mansoni/1-387          --DLNIYPSTSSISLTLNQAHVGTKTAMFTKNDL-KESLFKLNGKLLDVLIVGMSIFTILSchistosoma_japonicum/1-239        ------------------------------------------------------------Bmal/1-393                         --EDLMLPLNDSISLSIN--DMCAKTRVRIGASV-KKDSVSINGSNVC--LSKHPGFLRCTrichinella_spiralis/1-366         --EREMLPLNDSLSMNINELFVDTRVTISDG----SNDRVVLNGKEIV--GVQFSRFKRCCele/1-365                         --DLLILPLNDSISLTV--DRLTAETTIRMVPGV-GKHTVEINGKSVE--LSSNKRYQTVAtha/1-392                         L-----REIRGRAGDVED----------MEKGIKIRKKDWEKLNLHIASHNNFPTAAGLAgi|327287778_Acaronlinensis/1-406  L-----REIRRLARKRRS-------GDTKGPEGGEPSPLSLTYKVHIASENNFPTAAGLAgi|260794527_Bfloridae/1-406       L-----GEIRRLARKRKH--------------KDERAGDLLGSCVHVCSENNFPTAAGLAEcab/1-376                         L-----REIRRLARKRRS------------AGDEDLLPLSLSYKVHVASVNNFPTAAGLACfam/1-376                         L-----REIRRLARKRRS------------TGDEDPLPLSLTYKVHIASVNNFPTAAGLAMVD_Hs/1-376                       L-----REIRCLARKRRN------------SRDGDPLPSSLSCKVHVASVNNFPTAAGLAMmus/1-376                         L-----REIRRLARKRRS------------TEDGDTLPLSLSYKVHVASVNNFPTAAGLAMdom/1-376                         L-----REIRRLARKRRS--------------GSDGDLVPLSYKVHIASVNDFPTAAGLAOana/1-376                         L-----REIRRLARKRRS------------GRDGDSASLSLSYKVHIASVNNFPTAAGLAMVD_Acar/1-374                     L-----REIRRLARKRRS-------GDTKGPEGGEPSPLSLTYKVHIASENNFPTAAGLAXtro/1-376                         L-----REIRRLARKRRN------------EEGDENVSRILNDKVHICSVNNFPTAAGLAGgal/1-366                         L-----REVRRLARKRR--------------GDDAAAPLSLSYKVHVASENNFPTAAGLASalmo/1-376                        L-----REIRCLSRKRRS------------DGEADVDAAGLSHKVHICSVNNFPTAAGLATnig/1-376                         L-----KEIRRLARKRRN------------DENPSLESPVWSHKVHICSINNFPTAAGLADrer/1-376                         L-----LEIRRLAQRRKN---------------TGDPASDVSNKVHICSVNNFPTAAGLADC_Spur/1-371                      L-----QEIRKRARKRKI---------------SDDESNPEDWKLHICSENNFPTAAGLASaccoglossus_kowalevskii/1-378     L-----IEIRRRARKRKH--------------NDDSKSEMLNWSVHICSENNFPTAAGLATrichoplax_adhaerens/1-374         L-----REVRSRCGS-----------------------EIEGCHYHICSVNNFPTAAGLAAaeg/1-372                         L-----EEIKKRAKASNK-----------------CKPEILKWNVHVKTENNFPTAAGLADmel/1-374                         L-----NEVHRLAVASGS------------------QKVPPTWKLHIASVNNFPTAAGLATcas/1-372                         L-----TEIRKRANPKCG--------------------DLLNWKLHICSENNFPTAAGLAArabidopsis_lyrata/1-392           L-----REIRSRAGDVED----------KEKGIKIGKKDWEKLHLHIASHNNFPTAAGLAAtha2/1-385                        L-----REIRSRADDVED----------KEKGIKIAKKDWEKLHLHIASHNNFPTAAGLAArabidopsis_lyrata2/1-385          L-----REIRSRADDLED----------KEKGIKIEKKDWQKLHLHIASHNNFPTAAGLAPtri/1-389                         L-----REIRARACAVED----------KEKGIKIAKKDWEKLHLHVASYNNFPTAAGLARicinus_communis/1-389             L-----REIRARACDVED----------KEKGIKIAKKDWEKLHVHIASFNNFPTAAGLAVvin/1-392                         L-----REIRSRASKIED----------EKKGIKITKKDWEKLHVHIASYNNFPTAAGLAZea_mays/1-389                     L-----REIRKRARDFED----------KEKGVKIKKEDWDKLHVHIASYNNFPTAAGLAPpat/1-392                         L-----REMRARATDVVI----------ESSGKVIRKEDWNSLHIHIASENNFPTAAGLASmoe/1-388                         L-----KELRDRATDVKD----------EKTGIVITKEDWKHLKLHIVSKNNFPTAAGLASalpingoeca_sp./1-378              L-----GAVRALAAKKDP------------------EHPLKDAHIKIASVNNFPTAAGLAMonosiga_brevicollis/1-311         I-----RAMRAKAKAAHP------------------DSTLPEQKLLICSVNNFPTAAGLACapsaspora_owczarzaki/1-378        L-----REIRKLAAAKQT---------------HQQGAPLPTDKVHICSVNNFPTAAGLALbic/1-373                         I-----SELKRLRQATVE-------------NEDPSAPKLSTYKVHIASYNNFPTAAGLALbic2/1-373                        I-----FELKRLRQATVE-------------NEDPTAPKLSTYKVHIASYNNFPTAAGLACoprinopsis_cinerea/1-387          I-----REMKKLRKELVE-------------DKDANAPKLSTLPVHIASYNNFPTAAGLASchizophyllum_commune/1-382        I-----KEMKRLRKVEVE-------------DKDPSAPKLSTYHVRIASYNNFPTAAGLACryptococcus_neoformans/1-375      I-----KELRRWRKEMES--------------KDKDLPKLSEWPLRIASYNNFPTAAGLAKluyveromyces_lactis/1-380         L-----QDLRQLRRELEE--------------KDSSLPTFSQWKLHIASENNFPTAAGLAScer/1-379                         L-----RDLRQLRKEMES--------------KDASLPTLSQWKLHIVSENNFPTAAGLAAory/1-379                         L-----SNLRSLRQELEA--------------ADSSLPRLSTLPLRIVSENNFPTAAGLAYlip/1-370                         F-----RELRALRHKMED--------------SDSSLPKLADQKLKIVSENNFPTAAGLASchizosaccharomyces_pombe/1-368    V-----EELRKARLDLEE--------------ENDDLDKIGALKLHVVSENNFPTAAGLAThalassiosira_pseudonana/1-335     I-----DGLRALATDKVD---------PTTNEVIVSKSQWQSMHVHVASYNTFPTAAGLAPhaeodactylum_tricornutum/1-372    V-----DGVLALAPDKYH-----TDDDNNNKTVAIAQHEWPTLHVHVSSYNTFPTAAGLAEctocarpus_siliculosus/1-395       I-----RQVRALATEKRD---------EATGEVVVAEGDWDQYRVRIASRNTFPTAAGLAPhytophthora_infestans/1-384       L-----REMQQLAQRVHG--------------------ESDPQHLHIVSTNSFPTAAGLADictyostelium_purpureum/1-377      L-----KAIRSRATKLQD----------------------KKHCVHIVSINNFPTAAGLADictyostelium_discoideum/1-377     L-----KMIRSRATKLMD----------------------KKHCVHIASINNFPTAAGLAPolysphondylium_pallidum/1-378     L-----RMIRSRATKLMD----------------------KKHFVHICSINNFPTAAGLANaegleria_gruberi/1-386            F-----QEIRKAATAKWY---------------TERPNKDQEIYVHIDSTNNFPTAAGLANvec/1-377                         I-----SKVRQLAKENSP----------------ERWQELRNYGLCIYSKNNFPTAAGLALeishmania_major/1-372             L-----SCIRDNCPDNTK-----------------------NLKAYIVSENNFPTAAGMAgi|149241991|pdb|2HKE|A/1-372      L-----LHLRSTCPE-----------------------ELKNKKVNIVSENNFPTAAGMAEncephalitozoon_intestinalis/1-302 V-----EIFRTRSGDRRS--------------------------ICVRSFNNFPHSCGLAEncephalitozoon_cuniculi/1-303     V-----EIFRKKSGDDRP--------------------------VCIRSFSNFPHSCGLATthe/1-384                         LKFFEDKALAALGEELVPLQEGESQDTKRKTLKEFLNGDLSQLKLKIKSVNSFPTASGLAParamecium_tetraurelia/1-355       F-----GIFREQILQSKQFASNKYKNSPSDKPLGQVIPDIEKYGIRVESNNSFPTGSGLATrichomonas_vaginalis/1-341        I-----DFF----------------------------NDNGKLHFNITSVNSFPTAAGLASchistosoma_mansoni/1-387          F-----IKLAQLRSRLDG-------------------RLVPSPFLCVESENNFPTSAGLASchistosoma_japonicum/1-239        ------------------------------------------------------------Bmal/1-393                         F-----KEVRRLIRKRSI----------ISETAGKSEKHDYFSKFEVVSETNFPIEAGLATrichinella_spiralis/1-366         F-----DEARRIGGI--------------------------KQCFVVQSESLFPVSAGLACele/1-365                         F-----DEALRLQRKRKE-------AEASSADSNGNDPPPIFYHFHVTSTTNFPVAAGLAAtha/1-392                         SSAAGFACLVFSLAKLMN-VD-------EDPSHLSAIARQ--------------------gi|327287778_Acaronlinensis/1-406  SSAAGYACLVYTLAKLYG-VE----------GDLSEVARM--------------------gi|260794527_Bfloridae/1-406       SSAAGYACLVQSLAKLFH-ID----------GDVSHIARQ--------------------Ecab/1-376                         SSAAGYACLAYTLAQVYG-VE----------GDLSEVARR--------------------Cfam/1-376                         SSAAGYACLAYTLAQVYG-VD----------SDLSEVARR--------------------MVD_Hs/1-376                       SSAAGYACLAYTLARVYG-VE----------SDLSEVARR--------------------Mmus/1-376                         SSAAGYACLAYTLAQVYG-VE----------GDLSEVARR--------------------Mdom/1-376                         SSAAGYACLVYTLAQLYG-VE----------SELSEVARQ--------------------Oana/1-376                         SSAAGYACLVYTLARLYG-VE----------GELSEVARQ--------------------MVD_Acar/1-374                     SSAAGYACLVYTLAKLYG-VE----------GDLSEVARM--------------------Xtro/1-376                         SSAAGYACLVYTLAKLYG-VE----------GELSEIARQ--------------------Ggal/1-366                         SSAAGYACLVSALARLYG-VE----------GELSEVARR--------------------Salmo/1-376                        SSAAGYACLVYTLSRVMG-VE----------GELSAVSRQ--------------------Tnig/1-376                         SSAAGFACLVYTLARVFG-VE----------GELSAIARQ--------------------Drer/1-376                         SSAAGYACLVYTLSQLFN-VE----------GELSGVARQ--------------------DC_Spur/1-371                      SSAAGYACLVATLAQVYG-VQ----------GNVSDIARQ--------------------Saccoglossus_kowalevskii/1-378     SSAAGYACLVYTLSKLYD-IN----------GDVSDIARR--------------------Trichoplax_adhaerens/1-374         SSAAGYACLGEYFEYKEG-IT-----------KIQFTIRQ--------------------Aaeg/1-372                         SSASGYACLVYTLACLYG-IE---------NEEISSIARQ--------------------Dmel/1-374                         SSAAGYACLVYSLSRLYD-IP--------LNEELTTVARQ--------------------Tcas/1-372                         SSAAGYAALVSTLSALYN-VE----------GDISAIARR--------------------Arabidopsis_lyrata/1-392           SSAAGFACLVFSLAKLMN-VD-------EDPSHLSAIARQ--------------------Atha2/1-385                        SSAAGFACLVFALAKLMN-VN-------EDPSQLSAIARQ--------------------Arabidopsis_lyrata2/1-385          SSAAGFACLVFALAKLMN-VN-------EDPSQLSAIARQ--------------------Ptri/1-389                         SSAAGFACLVFALAKLMN-AK-------EDNSELSAIARQ--------------------Ricinus_communis/1-389             SSAAGFACLVFALAKLMN-AR-------EDNSELSAIARQ--------------------Vvin/1-392                         SSAAGFACLVFSLAKLMN-VQ-------EDQGKLSAIARQ--------------------Zea_mays/1-389                     SSAAGLACFVFTLGKLMN-AK-------EDYGELSSIARQ--------------------Ppat/1-392                         SSAAGFACLVYALAQLMG-VQ------EKYEGELTAIARL--------------------Smoe/1-388                         SSAAGFACLVFTVAQLMG-IK------ESFPGELSTIARR--------------------Salpingoeca_sp./1-378              SSAAGYACLVAALAELFG-VQ---------DQELTAIARV--------------------Monosiga_brevicollis/1-311         SSAAGYAALVAALAGLYD-LP---------VESLTDVARI--------------------Capsaspora_owczarzaki/1-378        SSAAGYACLVYALAQLYK-VG-------DSLAEVTKLARV--------------------Lbic/1-373                         SSASGFAALVASLAQLYA-LP-------VSPSTLSIIARQ--------------------Lbic2/1-373                        SSASGFAALVASLAQLYA-LP-------VSPSTLSIIARQ--------------------Coprinopsis_cinerea/1-387          SSASGFAALVSSLAHLYT-LT----PPLTSPSTLSLIARQ--------------------Schizophyllum_commune/1-382        SSASGFAALVSSLAALYK-LP-------VSPSTLSLIARQ--------------------Cryptococcus_neoformans/1-375      SSASGLAALVASLASLYS-LP-------QSPSQLSLVARQ--------------------Kluyveromyces_lactis/1-380         SSAAGFAALIKAIAKLYE-LP-------QSESELSKIARK--------------------Scer/1-379                         SSAAGFAALVSAIAKLYQ-LP-------QSTSEISRIARK--------------------Aory/1-379                         SSAAGFAALVRAVADLYQ-LP-------QSPRDLSRIARQ--------------------Ylip/1-370                         SSAAGFAALIRAVANLYE-LQ-------ETPEQLSIVARQ--------------------Schizosaccharomyces_pombe/1-368    SSAAGYAAFCEAIARLYD-LP-------WTPTQLSRIARQ--------------------Thalassiosira_pseudonana/1-335     SSAAGYAALVASLVELYN-AK------ESYPGEFTAIARQ--------------------Phaeodactylum_tricornutum/1-372    SSAAGYAALVAALVQLTGATE-------TFPGEFSTLARQ--------------------Ectocarpus_siliculosus/1-395       SSAAGLACLTFSLAKLFN-AK------ESFDGELSSIARQ--------------------Phytophthora_infestans/1-384       SSAAGYACLVAALAEFYG-VS---KADEEFTGQLSAIARQ--------------------Dictyostelium_purpureum/1-377      SSASGYCCLVYTLAQIYG-VD----------GDISGIARI--------------------Dictyostelium_discoideum/1-377     SSASGYCCLVFTLAQMYG-VD----------GDISGIARL--------------------Polysphondylium_pallidum/1-378     SSASGYACLVYVLAQLYG-VS----------GDISAIARI--------------------Naegleria_gruberi/1-386            SSASGYCCLVFALGQLFE-VK----------SDLSIIARL--------------------Nvec/1-377                         SSASGYACLVLALSKLYH-LD----------MELSSIARQ--------------------Leishmania_major/1-372             SSASGYCALAAALVKAYG-AT----------VDVSMLSRL--------------------gi|149241991|pdb|2HKE|A/1-372      SSASGYCAMSAALIRAFK-ST----------TNVSMLARL--------------------Encephalitozoon_intestinalis/1-302 SSASGFAALALALDDFYG-LK-------TSEEELCRIARI--------------------Encephalitozoon_cuniculi/1-303     SSASGLAALVLALNDFYG-LD-------MPEEELCIAARI--------------------Tthe/1-384                         SSASGLAALSVCLFDVYH-MK------EEYEFQRSVIARL--------------------Paramecium_tetraurelia/1-355       SSSSGLSALALCLQDILK-TD----------IDVRYLSRI--------------------Trichomonas_vaginalis/1-341        SSAAGAAAFVGALASLVGKTNNPITYWMQKGVDLTALARK--------------------Schistosoma_mansoni/1-387          SSASGTAAFAFALGTMYG-LD----------GDYTSLSRR--------------------Schistosoma_japonicum/1-239        ---------------MYS-LD----------GDYTSFSRR--------------------Bmal/1-393                         SSAAGFAAIAYGLGQIYQ-LN---------ISDIIRVARMGKHAVAIVILKCSQNELRLDTrichinella_spiralis/1-366         SSSAGFAAIAFAIGKMLN-WD---------VDTMSHVARL--------------------Cele/1-365                         SSAAGFAAIALAIQRILR-LD---------DSQANRLARI--------------------Atha/1-392                         ---GSGSACRSLFGGFVKWTM-GSK--------------------ED-GSDSVAVQLADEgi|327287778_Acaronlinensis/1-406  ---GSGSACRSMFGGFVQWVK-GED--------------------AD-GKESIAEQVAPEgi|260794527_Bfloridae/1-406       ---GSGSACRSMYGGFVEWTM-GRL--------------------ED-GADSVAKQVAPAEcab/1-376                         ---GSGSACRSLYGGFVEWQM-GQR--------------------AD-GKDSVARQVAPECfam/1-376                         ---GSGSACRSLYGGFVEWQM-GER--------------------AD-GKDSIARQVAPEMVD_Hs/1-376                       ---GSGSACRSLYGGFVEWQM-GEQ--------------------AD-GKDSIARQVAPEMmus/1-376                         ---GSGSACRSLYGGFVEWQM-GEQ--------------------AD-GKDSIARQIAPEMdom/1-376                         ---GSGSACRSMFGGFVQWHM-GER--------------------PD-GKDSIAQQVAPEOana/1-376                         ---GSGSACRSMYGGFVEWLM-GER--------------------PD-GKDSIAQQLVPEMVD_Acar/1-374                     ---GSGSACRSMFGGFVQWVK-GED--------------------AD-GKESIAEQVAPEXtro/1-376                         ---GSGSACRSMYGGFVQWVM-GER--------------------DD-GKDSLAKQVEPEGgal/1-366                         ---GSGSACRSMLGGFVQWHR-GER--------------------PD-GRDSVAQQLAPESalmo/1-376                        ---GSGSACRSMYGGFVQWLM-GQQ--------------------GD-GKDSLAQQVEPETnig/1-376                         ---GSGSACRSMYGGFVQWIM-GQR--------------------ED-GKDSIALQVEPDDrer/1-376                         ---GSGSACRSLYGGFVQWKL-GEQ--------------------SD-GKDSIAEQVASEDC_Spur/1-371                      ---GSGSACRSMYGGFVEWLD-GES--------------------SC-GSDSIAQQVVDESaccoglossus_kowalevskii/1-378     ---GSGSACRSIYGGFVQWTV-GEK--------------------KN-GSDSIAKVVADVTrichoplax_adhaerens/1-374         ---GSGSACRSMYGGFVKWEM-GNK--------------------SD-GSDSIAVQVTPEAaeg/1-372                         ---GSGSACRSLHSGFVQWQK-GEH--------------------PD-GSDSVAVQLVPHDmel/1-374                         ---GSGSACRSLYGGFVQWHR-GAL--------------------DD-GSDSVARQIAPSTcas/1-372                         ---GSGSACRSIYGGFVRWNK-GAK--------------------PG-GEDSIACQIASAArabidopsis_lyrata/1-392           ---GSGSACRSLFGGFVKWTM-GSK--------------------ED-GSDSVAVQLADEAtha2/1-385                        ---GSGSACRSLFGGFVKWNM-GNK--------------------ED-GSDSVAVQLVDDArabidopsis_lyrata2/1-385          ---GSGSACRSLFGGFVKWNM-GNK--------------------ED-GSDSVAVQLVDDPtri/1-389                         ---GSGSACRSLFGGFVKWIM-GKA--------------------ED-GSDSLAVQLVDERicinus_communis/1-389             ---GSGSACRSLFGGFVKWIM-GKV--------------------DD-GSDSLAVQLVDEVvin/1-392                         ---GSGSACRSLYGGFVKWVM-GNE--------------------EN-GSDSIAVQLQDEZea_mays/1-389                     ---GSGSACRSIYGGFVKWCM-GEK--------------------DD-GSDSIAVQLADEPpat/1-392                         ---GSGSACRSLYGGFVEWKM-GQE--------------------ID-GTDSIAVQLAEESmoe/1-388                         ---GSGSACRSLHGGFVKWEM-GKR--------------------DD-GKDSIAVPLAEHSalpingoeca_sp./1-378              ---GSGSACRSLMGGFVRWEK-GTR--------------------DD-GADSLASQVVPEMonosiga_brevicollis/1-311         ---GSGSACRSLSGGFVRWRR-GEL--------------------AD-GTDSLASQVVPECapsaspora_owczarzaki/1-378        ---GSGSACRSIYGGWVRWVM-GEA--------------------AD-GSDSIAEQVVDELbic/1-373                         ---GSGSACRSLYGGFVAWQE-GVL--------------------PD-GSDSLAVQVAPQLbic2/1-373                        ---GSGSACRSLYGGFVAWQE-GVH--------------------PD-GSDSLAIQVAPQCoprinopsis_cinerea/1-387          ---GSGSACRSLFGGFVAWEM-GST--------------------PT-GTDSLAVQIADESchizophyllum_commune/1-382        ---GSGSACRSLYGGFVAWEQ-GTK--------------------AD-GSDSLAIQIAPECryptococcus_neoformans/1-375      ---GSGSACRSLFGGFVAWRE-GTD--------------------PA-GSDSLAEEVAPRKluyveromyces_lactis/1-380         ---GSGSACRSLFGGYVAWEM-GKL--------------------ED-GSDSKAVEIGSLScer/1-379                         ---GSGSACRSLFGGYVAWEM-GKA--------------------ED-GHDSMAVQIADSAory/1-379                         ---GSGSACRSLMGGYVAWRA-GNL--------------------AD-GSDSLAEEVAPEYlip/1-370                         ---GSGSACRSLYGGYVAWEM-GTE--------------------SD-GSDSRAVQIATASchizosaccharomyces_pombe/1-368    ---GSGSACRSLFGGYVAWEM-GEL--------------------HS-GADSVAVQVEPVThalassiosira_pseudonana/1-335     ---GSGSACRSLYGGFVAWRAGGMK--------------------ED-WSDSIAEQVADEPhaeodactylum_tricornutum/1-372    ---GSGSACRSLYGGLVAWHA-GTA--------------------DEQWRDSRAEQLADEEctocarpus_siliculosus/1-395       ---GSGSACRSLYGGFVKWQK-GVR--------------------ED-ARDSIAVQVADEPhytophthora_infestans/1-384       ---GSGSACRSLDGGFVAWQK-GER--------------------PD-GHDSIAVQVADEDictyostelium_purpureum/1-377      ---GSGSACRSMYGGFVKWEM-GEK--------------------ED-GSDSIAVQVQPEDictyostelium_discoideum/1-377     ---GSGSACRSMYGGFVKWEM-GTK--------------------DD-GSDSIAVQVQPEPolysphondylium_pallidum/1-378     ---GSGSACRSVYGGFVKWEM-GAE--------------------SD-GSDSIAVQVAPENaegleria_gruberi/1-386            ---GSGSACRSLYGGYVAWEK-GHD--------------------HE-TSKAIQVLDEHDNvec/1-377                         ---GSGSACRSMYGGFVKWEA-GCR--------------------PD-GTDSIASQIVDELeishmania_major/1-372             ---GSGSACRSVYGGFVIWHK-GEK--------------------PD-GTDCIATQFLDEgi|149241991|pdb|2HKE|A/1-372      ---GSGSACRSAFGGFVIWNK-GEK--------------------PD-GSDCVATQFVDEEncephalitozoon_intestinalis/1-302 ---GSGSAGRSISPGIHLFDG---------------------------------VFVEKLEncephalitozoon_cuniculi/1-303     ---GSGSAGRSISTGIHLFDG---------------------------------MSVERLTthe/1-384                         ---GSGSASRSIYGGLVEWTGVPHQYLQKKFESKNNEIQLSEQEYEQLSKLCIAKQTHNEParamecium_tetraurelia/1-355       ---GSGSACRCLYGNLVLFPE-TIS--------------------LE-SKRCLPYEV-QSTrichomonas_vaginalis/1-341        ---VSGSGCRSIHGGFVEWVP-GTP--------------------SE----SVAKQIADQSchistosoma_mansoni/1-387          ---GSGSSCRSLLGGFVQWSN------------------------NH-DDHTSVQQLFPASchistosoma_japonicum/1-239        ---GSGSSCRSLSGGFVLWSS-NRG--------------------DY-LHPSFVQQLFPSBmal/1-393                         NYSGSGSACRSILSGLVHWKA-GTA--------------------ED-GTDCICETVFPETrichinella_spiralis/1-366         ---GSGSACRGVYPGFVHWMAELAQ--------------------SN-DTRNKCEVVALPCele/1-365                         ---GSGSACRSMYGGLVHWRK-GEM--------------------DD-GSDCLAVRTEAAAtha/1-392                         KHWDDLVIIIAVVSS---RQKETSSTSGMRESVETSLLLQHRAKEVVPKRILQMEEAIKNgi|327287778_Acaronlinensis/1-406  THWPEMRVLILVVSA---EKKPIGSTAGMQTSVETSHLLKHRAEKLVPEYMAQMTRHIRRgi|260794527_Bfloridae/1-406       EHWPELRVLVAVVNA---GKKAVGSTEGMQTTVKTSALVKYRAEHVVPSRQEDMRQAILEEcab/1-376                         LHWPELRVLILVVSA---EKKLTGSTVGMQTSVETSPLLRFRAEALVPARMAEMARCVMECfam/1-376                         SHWPELRVLILVVSA---EKKLMGSTAGMQTSVETSPLLRFRAESVVPARMAEMTRCIQEMVD_Hs/1-376                       SHWPELRVLILVVSA---EKKLTGSTVGMRASVETSPLLRFRAESVVPARMAEMARCIREMmus/1-376                         WHWPQLRILILVVSA---DKKQTGSTVGMQTSVETSTLLKFRAESVVPERMKEMTRCIQEMdom/1-376                         SHWPELRVLVLVVSA---ERKPVSSTSGMQTSVETSSLLKFRAESVVPGRMAEMARCIKEOana/1-376                         THWPELRVLILVVSA---EKKSVGSTAGMQTSVETSPLLKFRAESVVPGRMAEMRRCIQEMVD_Acar/1-374                     THWPEMRVLILVVSA---EKKPIGSTAGMQTSVETSHLLKVGSGMC-PCHAQD--GPFRRXtro/1-376                         SHWPELRVLILVATA---EKKHVGSTAGMQTSVETSPLLKLRADLVVPERMEAMIESIRKGgal/1-366                         THWPELSVLVLVVSG---EKKAVGSTAGMQTSVDTSPLLKYRAEVVVPERMTRMARCIRDSalmo/1-376                        THWPELRVLVLVVSA---ERKPVGSTSGMQTSVETSILLKHRADSVVPARMKEMIEAVHKTnig/1-376                         SHWPELRILVLVASA---EKKPVGSTAGMQTSVETSCLLKYRAESVVPGRLAEMIQAVRRDrer/1-376                         LYWPELRVLILVVSA---EQKSVGSTSGMHTSVETSHLLKYRADAVVPGRMEEMIRAIRLDC_Spur/1-371                      NYWSEMRILILVVSN--------------------LSFMSHH-----HGYMETMRKAIKDSaccoglossus_kowalevskii/1-378     DHWPEMRVLVLVVSD---QKKHTSSTNGMRNSVNTSDFLRYRAEHVVPSRMEEMIKAIEETrichoplax_adhaerens/1-374         SHWPEMEVLILVVSD---KKKGVSSTSGMQTSVKTSKLLKYRAESLVPKLMTEMETAIQQAaeg/1-372                         DFWPEMRIIVLVVND---ARKKTSSTGGMSTSVKTSKLLKYRVEECVPKHTKDLVEALNKDmel/1-374                         DHWPNMHVLILVVND---ARKKTASTRGMQQAVKTSQLIKHRVDQVVPDRIIRLREAIASTcas/1-372                         SHWPEMRVLILVVSD---DQKKYSSTSGMKQSVLTSELLKHRAEKIVPGRVDEIIKAIKLArabidopsis_lyrata/1-392           KHWDDLVIIIAVVSS---RQKETSSTSGMRESVETSLLLQHRAKEVVPKRILQMEEAIKNAtha2/1-385                        KHWDDLVIIIAVVSS---RQKETSSTSGMRESVETSLLLQHRAKEVVPVRILQMEEAIKNArabidopsis_lyrata2/1-385          KHWDDLVIIIAVVSS---REKETSSTSGMRESVETSLLLQHRAKEVVPVRILQMEEAIKNPtri/1-389                         KHWDELVIIIAVVSS---RQKETSSTTGMRDSVETSLLLQHRAKEVVPKRIKQMEEAIKNRicinus_communis/1-389             KHWDDLVIIIAVVSS---RQKETSSTSGMRESVETSLLLQHRAKEVVPKRIIQMEEAINKVvin/1-392                         KHWDELVIIIAVVSS---RQKETSSTSGMRDSVETSLLLQHRAKEVVPKRIIEMEEAIKNZea_mays/1-389                     THWNDLVIIIAVVSS---KQKETSSTSGMRDSVETSPLLQYRAQTVVPGRVLKMEEAIKNPpat/1-392                         SHWKDLVIIIAVVSS---RQKETSSTSGMQESVKTSPLLKYRAEEVVPKRIVQMEKAIKSSmoe/1-388                         HEWDDLRIVICVVSS---RQKEVSSTSGMQESVQTSPLLHYRAKEVVPKRITEMEEALSKSalpingoeca_sp./1-378              SHWPDMQVLILVVNA---GKKGVSSTSGMQSTVKTSALINHRAEVVVPQRMKDIEKAIQDMonosiga_brevicollis/1-311         SHWPEMEVLILV------------------------------------------------Capsaspora_owczarzaki/1-378        HHWPEIEVLILVVSD---HKKTTSSTAGMQTTVETSSLVKHRADKVVPQRMEDIQNAIRALbic/1-373                         SHWPEIHALICVVSD---DKKGTSSTSGMQLTVETSTLLQHRIKAVVPQRMKDISKAILELbic2/1-373                        SHWPEIHALICVVSD---DKKGTSSTSGMQLTVETSPLLQHRIKAVVPQRMKDISKAILECoprinopsis_cinerea/1-387          AHWPEMHALICVVSD---DKKGTSSTAGMQRTVETSTLLQHRIKDVVPRRMDEMIRAIKESchizophyllum_commune/1-382        SHWPTLHAVVCVVND---AKKGTSSTAGMQRTVETSPLLQHRIKHVVPQRMAEISDAIRACryptococcus_neoformans/1-375      EHWPEMHALICVVSD---AKKGTSSTSGMQKTVETSTLLQERLR-IVPKRMDAISQAIKAKluyveromyces_lactis/1-380         NHWPEMKAAILVVSA---DKKDTPSTSGMQLTVKTSDLFQERINNVVPKRFEQMKKSILEScer/1-379                         SDWPQMKACVLVVSD---IKKDVSSTQGMQLTVATSELFKERIEHVVPKRFEVMRKAIVEAory/1-379                         SHWPEMRALILVVSA---EKKDVPSTEGMQTTVATSNLFATRAESVVPERMAAIETAIQNYlip/1-370                         DHWPEMRAAILVVSA---DKKDTSSTTGMQVTVHTSPLFKERVTTVVPERFAQMKKSILDSchizosaccharomyces_pombe/1-368    ENWPEIRVAVLVASA---AKKGVSSTAGMQATVASSTLFQHRIQNIVPQRIQEMKTAIREThalassiosira_pseudonana/1-335     MHWKEMRAVILVVSD---AKKETSSTVGMETSVATSELLAHRAKEIVPKRMKIIEDAIQAPhaeodactylum_tricornutum/1-372    ASWPALRAVIAVVSD---AQKDTASTAGMQASVKTSPLLAFRAAHVVPQRMQELTQAWRREctocarpus_siliculosus/1-395       HHWPEMRALILVVSA---DKKDTSSTSGMSTSVQTSPLLGFRAKEVVEPRLAEIEKAYLEPhytophthora_infestans/1-384       LHWPELCAVVCVVND---AQKDTSSTTGMQTSKATSSLLAYRAKHLVPERMQTMEQAILADictyostelium_purpureum/1-377      SHWPEMNIIVLVVND---KKKETSSTDGMQKSAATSPMMKERCAVTVPTRMRDIEEAIKNDictyostelium_discoideum/1-377     SHWPDMNIIVLVVND---KKKETSSTDGMQKSAATSVMMKERCAVTVPNRMRDIEEAINKPolysphondylium_pallidum/1-378     THWPEMNIIVLVVND---KKKETSSTDGMQRSAATSPMMKERCATIVPQRMRDIEAAIQANaegleria_gruberi/1-386            DFSKQTNIVVCVVSD---RQKHTPSTSGMQQSVITSKLLKVRASEIVPQRMIEMDKALKTNvec/1-377                         KHWSTLRILILVIND---ERKANPSTSGMRRSTETSELLQFRAQKCVPKRMENITKAIKELeishmania_major/1-372             KYWPEVQVMCAVLKG---EKKDVSSTSGMQQSLKTSSMMRERIESIVPARMSAVKEAIQQgi|149241991|pdb|2HKE|A/1-372      THWPEIQVMCAVLKG---AQKDVSSTKGMQQSLKTSPLMKKRISETVPERMKIASRAIKAEncephalitozoon_intestinalis/1-302 PSWREIKILSIVLSK---DPKKIGSTEGMIRTRETSEFYQERLARM-KEKVEAMAKCISQEncephalitozoon_cuniculi/1-303     PSWKEVRILSIILSG---DCKKTGSTEGMIRTKETSNFYQERLARI-ERKIKAMVQYISQTthe/1-384                         TFFEDLDVFVVAYSF---ESKEVPSTSGMLQSTQTSELLKYRALNTAHVHIAGVKKAIEEParamecium_tetraurelia/1-355       SKWLKDKVSIVILTDTHQGQKDVLSKDGMKLTWETSKLIQGRVRQYVEQHITELQSALEKTrichomonas_vaginalis/1-341        HQWEDFVVFSVIVSS---KKKDVLSTKGMQSTVETVPWIHWRAQEVVPKRISDAKKFINESchistosoma_mansoni/1-387          SYWPELRVLICVTNE---NPKPVGSTDAMLCCVKTSYLFRNGRVPSSKIHEKEIISALKDSchistosoma_japonicum/1-239        SHWPELKVLICVVNE---HSKHIGSTDAMLNCVNTSDLFRSGRVLSAKIHEKQAISALREBmal/1-393                         DYWPTLRSLILVTSH---GTKKVSSSNGMQSTVKTSKLLQARMD-IVPEQITKLRNAFRDTrichinella_spiralis/1-366         EHWPELTVIVLIGSD---EAKRWSSTDGMRRSVATSKLLKYRAECCVPERIEKVRRAIQACele/1-365                         ANWEDLYCIILVFDD---GRKKVGSSEGMRRSRETSQLLKHRIESIVPQRIQQIQEAYTSAtha/1-392                         RDFASFTQLTCTDSNQFHAVCLDTSPPIF--YMNDTSHRIISLVEKWNRSE-----GTPQgi|327287778_Acaronlinensis/1-406  RDFEAFGELTMKDSNQLHATCLDTFPPIF--YLNDISKQVVRLVHRFNDHY-----GKTKgi|260794527_Bfloridae/1-406       RDFQTFGEITMKDSNQFHATCLDTYPPIF--YLNETSKHIIHLVHRYNRHH-----GKIKEcab/1-376                         RDFQAFGQLTMKDSNQFHATCLDTFPPIS--YLNDTSRCIIHLVHRFNAHH-----GQTKCfam/1-376                         RDFQGFGQLTMKDSNQFHATCLDTFPPIS--YLSDTSRRIVHLVHRFNTHH-----GQTKMVD_Hs/1-376                       RDFPSFAQLTMKDSNQFHATCLDTFPPIS--YLNAISWRIIHLVHRFNAHH-----GDTKMmus/1-376                         QDFQGFAQLTMKDSNQFHATCLDTFPPIS--YLNDTSRRIIQLVHRFNTHQ-----GQTKMdom/1-376                         RDFEAFGQLTMKDSNQFHATCLDTFPPIC--YLNDTSRQIISLVHCFNAYY-----GKTKOana/1-376                         KDFQGFGLLTMKDSNQFHATCLDTFPPIC--YLNDTSRHIISLVHRFNAHF-----GKTRMVD_Acar/1-374                     RDFEAFGELTMKDSNQLHATCLDTFPPIF--YLNDISKQVVRLVHRFNDHY-----GKTKXtro/1-376                         KDFKAFGELTMKDSNQFHATCLDTYPPIF--YLNSVSQRVISVVHQYNTYY-----GQTKGgal/1-366                         RDFEAFGQLTMQDSNQFHATCLDTFPPIF--YLNDISQRIIALAHRFNAHH-----GRTKSalmo/1-376                        RDFTAFAELTMKDSNQFHATCLDTYPPIF--YLNDVSRRVINLVHRYNRHY-----RETKTnig/1-376                         RDFATFAELTMKDSNQFHATCLDTYPPIF--YLSSVSQQVINLVHRYNRHY-----GEMRDrer/1-376                         RDFPKFGELTMKDSNQFHAICLDTYPPIF--YLNNISHQIISLVHRYNQYY-----GETRDC_Spur/1-371                      RDYRTFAELTMKDSNQMHAVCLDTYPPIS--YMNDTSRSIVQMVHDYNSFH-----GETKSaccoglossus_kowalevskii/1-378     KDYQKFAELTIKDSNQMHAVCLDTYPPIS--YMNDTSRKIINMIHAFNKYQ-----GELKTrichoplax_adhaerens/1-374         KNYQAFAEITMKDSNQFHAVCLDTYPPIA--YMNDISHKIVQLITHFNQYC-----GEYKAaeg/1-372                         KDFETFGKITMQDSNQFHAVCLDTYPPCV--YMNDISFAVVNMVHQFNALK-----KEVKDmel/1-374                         HDFQAFAEITMKDSNQFHAIALDTYPPCV--YMNDVSHSIVSFVHDYNERM-----GSYHTcas/1-372                         KNFEAFAKITMQDSNQFHAICLDTYPPCF--YMNDVSRMIIELVHAYNDYQ-----GATKArabidopsis_lyrata/1-392           RDFASFTQLTCTDSNQFHAVCVDTSPPIF--YMNDTSHRIISLVEKWNRSE-----GTPQAtha2/1-385                        RDFTSFTKLTCSDSNQFHAVCMDTSPPIF--YMNDTSHRIISLVEKWNRSA-----GTPEArabidopsis_lyrata2/1-385          RDFTSFTKLTCSDSNQFHAVCMDTSPPIF--YMNDTSHRIISLVEKWNRSA-----GTPEPtri/1-389                         RDFGSFAQLSCADSNQFHAVCLDTCPPIF--YMNDTSHRIISCVEKWNCSE-----GTPQRicinus_communis/1-389             RDFASFAQITCADSNQFHAVCLDTCPPIF--YMNDTSHRIISCVEKWNRSE-----ETPQVvin/1-392                         RDFPSFARLTCADSNQFHAVCLDTSPPIF--YMNDTSHRIISCVEKWNRSE-----GTPQZea_mays/1-389                     RDFESFAKLTCADSNQFHAVCLDTSPPIF--YMNDTSHRIISLVEKWNHSE-----GTPQPpat/1-392                         MNFTEFAKITCADSNQFHATCLDTSPPIF--YLNDTSRKLIGLVERWNRHA-----GEPQSmoe/1-388                         RDFSSFAKLTCADSNQFHATCLDTSPPIF--YMNDTSRRIIGLVERWNKSE-----GSPQSalpingoeca_sp./1-378              RDFQTFGRITMQDSNQFHATCLDTYPPIF--YMNDVSRQIVQILTQYNDAA-----GEIRMonosiga_brevicollis/1-311         ------------------------------------------LLTKFNAQS-----SSPRCapsaspora_owczarzaki/1-378        RDFETFGRITMQDSNQFHAVCLDTYPPIT--YLNDVSRGIIDMLTKYNAHK-----GKIQLbic/1-373                         KDFDTFARITMADSNQFHAVALDTEPPIF--YMNDVSRAIIAVIVEYNRLSLANG-QGYKLbic2/1-373                        KDFDTFARITMADSNQFHAVALDTEPPIF--YLNDVSRAIIAVIVEYNRLSLANG-QGYKCoprinopsis_cinerea/1-387          KDFDSFARITMADSNSFHAVALDTEPPIF--YMNDVSRAIIALIVELNRVSLEKG-EGYKSchizophyllum_commune/1-382        RDFDAFARITMQDSNQFHAVALDTDPPIF--YMNDVSKAIVALIVEYNRVAIEKT-GKRKCryptococcus_neoformans/1-375      RDFSEFAKLTMADSNSFHAVCLDTAPPIF--YLNDVSRAIIAVVEELNRAA-----GEIIKluyveromyces_lactis/1-380         KDFPTFAELTMKDSNSFHATCLDSYPPIF--YLNDTSKKVIKLCHAINEFY-----NETVScer/1-379                         KDFATFAKETMMDSNSFHATCLDSFPPIF--YMNDTSKRIISWCHTINQFY-----GETIAory/1-379                         RDFPAFAEITMRDSNGFHATCLDSWPPIF--YMNDVSRAAVRLVHDINRAV-----GRTVYlip/1-370                         RDFPTFAELTMRDSNQFHATCLDSYPPIF--YLNDVSRASIRVVEAINKAA-----GATISchizosaccharomyces_pombe/1-368    RDFETFAKLTMTDSNQFHACCLDTFPPIF--YLNDTSRAVIRVVENINATA-----GKTIThalassiosira_pseudonana/1-335     KDFEAFGKVTMMDSNQFHATCLDTYPPIF--YMNDVSRSVIQMVTRYNEWA-----GEIRPhaeodactylum_tricornutum/1-372    RDFPVFGKITMQDSNQFHATCLDTYPPIF--YMNDVSRQIIRIVTAYNDYA-----GEIREctocarpus_siliculosus/1-395       KDFATFGKITMQDSNQFHATCLDTYPPIF--YMNDVSRSVIRIVHAYNAFH-----GEIRPhytophthora_infestans/1-384       RDFEAFGTLTMQDSNHFHATCLDTTPPIF--YLNDVSRQIIHLVHRYNKQA-----GRVQDictyostelium_purpureum/1-377      KDFQTFGDITMKDSDDFHEVCATTTPAIY--YLNDTSRYIMNLIHRYNKLS-----GSVKDictyostelium_discoideum/1-377     KDFQTFGDITMKDSDDFHEVCATTTPPIY--YLNDTSRYIMNLIHRYNKLS-----GSIKPolysphondylium_pallidum/1-378     RDFQTFGDITMKDSDDFHEVCATTDPAIY--YLNDTSRYIMNLVHKYNKMS-----GKIKNaegleria_gruberi/1-386            KDFNLFATLTMDDSDNMHACCADTEPAIY--YMNETSNQIVQLVKDFNAFDDGNGVENLKNvec/1-377                         RDFHTFAEITMKDSNQLHAVCQDTYPPITPPYMNSTSHLVVQLVTAYNNNH-----GNNKLeishmania_major/1-372             RDFNQFAAIAMADSDDLQEICRTTKPPIQ--YATDDSYAMIRLIRAFNAKK-----GYNVgi|149241991|pdb|2HKE|A/1-372      RDFATFAEIAMLESDDLQEICATTEPKIT--YATEDSYAMIRLVKAYNAKK-----GRTAEncephalitozoon_intestinalis/1-302 KDFDGFAYLTMRESNEFHGMLMETYPPIR--YIKDDGFQVIEMCHRFNR-------DKVREncephalitozoon_cuniculi/1-303     KDFDGFAHLTMRESNELHAILMETYPPIR--YIRDDGFKVIEMCHEFNR-------DRTRTthe/1-384                         KNYNELARLVRLDSNQFHAVCLDTTPPIF--YLNDFSKNMINFIHQLDSAL------EYHParamecium_tetraurelia/1-355       QDFNKVMEIIIKDSNQFHATCMDTYPPLL--YLNDFSRQIIKMVHIFNRN------AKHITrichomonas_vaginalis/1-341        KDFASLAEIIMRESNELHANCLATFPPIK--YLNDESFKVVSAIHQLNDDH-----KINISchistosoma_mansoni/1-387          RDFSALAEVTMRESNQLHALCLDTWPPCI--YLNELSHSIMDFVHSINNYF-----MKNVSchistosoma_japonicum/1-239        RDFSALAEVTMRESNQLHAVCLDTWPPCV--FLNHLSYSIMDFVHRINKYF-----KKSVBmal/1-393                         RNFEQLAKVIMSDSGQLHALCMDTMPSLR--YLNDNSWYLMQLIHALNRHC-----KDTKTrichinella_spiralis/1-366         RDFAKLAVEVMRDSCQLHAICLDTYPPLL--YLTEFSRQVMLMVHHYNDVC-----GRPKCele/1-365                         RNFEQLARVIMADSNQFHAVCMDSTPPIR--YLNEASWQLIDTVEEFNI-------GGIRAtha/1-392                         VAYTFDAGPNAVLIARNRKVAVQLLQGLLYYFPPKSDTDM---KS--------------Ygi|327287778_Acaronlinensis/1-406  VAYTFDAGPNAVVFMMEETVAE-FVEVVKRSFPPENNGGQ-------------------Fgi|260794527_Bfloridae/1-406       AAYTFDAGPNAVLYLLQDDVPE-VLALLRHFFPPSSTNNS--ERE--------------FEcab/1-376                         VAYTFDAGPNAVIFTLDDTMAE-FVAAVRHSFPPESNGDK-------------------FCfam/1-376                         VAYTFDAGPNAVVFTLEDTVPE-FVAAVQHCFPPESNGDK-------------------FMVD_Hs/1-376                       VAYTFDAGPNAVIFTLDDTVAE-FVAAVWHGFPPGSNGDT-------------------FMmus/1-376                         VAYTFDAGPNAVIFTLEDTVAE-FVAAVRHSFPPAANGDK-------------------FMdom/1-376                         VAYTFDAGPNAVIFTLEETVDE-FVAVIKQVFPPEMNGDK-------------------FOana/1-376                         VAYTFDAGPNAVIFTLDNTVDD-FVAVVKHSFPPVSNGDQ-------------------FMVD_Acar/1-374                     VAYTFDAGPNAVVFMMEETVAE-FVEVVKRSFPPENNGGQ-------------------FXtro/1-376                         VAYSFDAGPNAVIFMLEPTVNE-FVEVVKHCFPPESNGDT-------------------YGgal/1-366                         VAYTFDAGPNAVVFMLEDTVDE-FVEVVRRSFPPDSNGD---------------------Salmo/1-376                        VAYTFDAGPNAVIYTLQQNVEE-FVQVVKHFFPPETNGGH-------------------FTnig/1-376                         VSYTFDAGPNAVIFTLEQHVSE-FLQVVQHFFPPEANGGH-------------------FDrer/1-376                         VAYTFDAGPNAVIYSLQDYLPE-FVEVVRHFFPPEVNEEE-------------------FDC_Spur/1-371                      ACYTFDAGPNAVLYVLEENVSE-VLSLIHHSFPPSQDNKE-------------------YSaccoglossus_kowalevskii/1-378     VAYTYDAGPNAVLYLLDEHVPD-VVSLINYYFPPCDNIRE----T--------------FTrichoplax_adhaerens/1-374         ACYTFDAGPNAVLYVLAKDVPQ-ILSAVCHYFPCTENHDS-------------------YAaeg/1-372                         VAYTFDAGPNACLYLLEKDVPE-VLAVVNKVFPNDKLGDP----E--------------YDmel/1-374                         AAYTFDAGPNACLYVLAEHVPH-LLSAIQKVFPNDLADGG----T--------------YTcas/1-372                         VAYTFDAGPNACLYLLQNDVDE-VASLINDIFPSNTNPSE-------------------FArabidopsis_lyrata/1-392           VAYTFDAGPNAVLIARNRKVAVQLLQGLLYYFPPKSDTDM---KS--------------YAtha2/1-385                        IAYTFDAGPNAVMIARNRKVAVELLQGLLYCFPPKPDTDM--------------------Arabidopsis_lyrata2/1-385          IAYTFDAGPNAVLIARNRKVAVELMQGLLYCFPPKPDTDM--------------------Ptri/1-389                         VAYTFDAGPNAVLIAHNRKAATQLMQKLLFCFPPSSDADL---NS--------------YRicinus_communis/1-389             VAYTFDAGPNAVLIAQNRKTAVQLLQKLLYYFPPNSDTDL---NS--------------YVvin/1-392                         VAYTFDAGPNAVLIARDRKVAANLLQRLLYYFPPHSDTDL---NS--------------YZea_mays/1-389                     VAYTFDAGPNAVLIAQNRKTAAHLLQKLLYYFPPQDNDLS----S--------------YPpat/1-392                         VAYTFDAGPNAVMFAKNKEVAAQLLQRLLYQFPPSADTDI---SR--------------YSmoe/1-388                         AAYTFDAGPNAVIFVPQKSGGA-LLHRLLYEFPPPEGMNL---SS--------------YSalpingoeca_sp./1-378              AAYTYDAGPNCVIYCLKQHVQE-ILSLVCHYFPSSESE---------------------FMonosiga_brevicollis/1-311         AAYTYDAGPNCVIYALKKDIPD-IIALVARCFPSSTPAT--------------------YCapsaspora_owczarzaki/1-378        AAYTFDAGPNAVIYLPRENVNE-VVNLVRHFFPPAVGAEA----A--------------FLbic/1-373                         AAYTYDAGPNAVIYAEEKNIKE-IIQLIVSFFPQREGT----------------------Lbic2/1-373                        AAYTYDAGPNAVIYTEEKNIKE-IIQLIVSFFPQKEGT----------------------Coprinopsis_cinerea/1-387          AAYTYDAGPNAVIYTLDKNVKE-VIQLIVKYFPQKAGEFKDNLQV--------------LSchizophyllum_commune/1-382        AAYTYDAGPNAVIYVEQENVKE-IVDLILQYFPDAAANFKDVFNL--------------YCryptococcus_neoformans/1-375      AAYTFDAGPNAVIYTLEKNMPV-VLGAIKRFFPTGEEFEDPF------------------Kluyveromyces_lactis/1-380         VAYTFDAGPNAVLYYLEQSEDK-LFAFLYHLFQNVSGWESKFTKE--------------QScer/1-379                         VAYTFDAGPNAVLYYLAENESK-LFAFIYKLFGSVPGWDKKFTTE--------------QAory/1-379                         CAYTFDAGPNAVIYYLEKDSEL-VAGTVKAILGASSEGWDGPFYE--------------PYlip/1-370                         AAYTFDAGPNCVIYYEDKNEEL-VLGALKAILGRVEGWE---------------------Schizosaccharomyces_pombe/1-368    AAYTFDAGPNAVIYFLEENSEI-VLNTLYAVTKNAEGWSK--------------------Thalassiosira_pseudonana/1-335     AAYTFDAGPNAVLYTLDKYVVE-LLALVLKHYP---------------------------Phaeodactylum_tricornutum/1-372    AAYTLDAGPNVVLYVLEPHRPV-LAALLRHFFPASGLEEQ--------------------Ectocarpus_siliculosus/1-395       AAYTFDAGPNAVVYHLAGDSAE-LLALLLRFYPAPAGSSTSNGST--------------SPhytophthora_infestans/1-384       AAYTFDAGPNAVIFVEEQHVQE-VVSLVHHCFPTSSEMTIKSSIQ--------------VDictyostelium_purpureum/1-377      CAYTFDAGPNACIYLPEENVVE-VLSLFIKHFPDQDLSTYYRGPK--------------EDictyostelium_discoideum/1-377     CAYTFDAGPNACIYLPAESTTE-VLSLFMKHFPGDDMQTYYRGPK--------------EPolysphondylium_pallidum/1-378     CAYTFDAGPNACIYLPEENVVE-ALALFTKHFPGSDLSTYYRGSD--------------KNaegleria_gruberi/1-386            VAYTFDAGPNAVLFFPNKEVTNKFLAILHAFFPPSNQEQFFSKEP--------------FNvec/1-377                         VAYTFDAGPNSVLFTQEGDLPE-LVALIKHFFPPASGKS--------------------FLeishmania_major/1-372             MAYTFDAGANCFMFTLKQDLPE-VVVMLRAHFPTSWDKLLFHDAD--------------Lgi|149241991|pdb|2HKE|A/1-372      LAYTFDAGANCFLFVLKEDLPE-AVAMLMEHFPTPFEK---------------------FEncephalitozoon_intestinalis/1-302 VAYTFDAGPNPFIITLEQYLKE-VKDFFRAYELVPCNY----------------------Encephalitozoon_cuniculi/1-303     VAYTFDAGPNPFLITLEQHLHA-VEDFFKAYELVPCNY----------------------Tthe/1-384                         VAYTFDAGPHAVLLVHKNHTTQ-VLRAIYEAFS---------------------------Paramecium_tetraurelia/1-355       VGYTFDAGAHAVLLIHNDELQS-----FKKFLSEAEN-----------------------Trichomonas_vaginalis/1-341        AAYSFDAGPNPFVFTTKEHEKA-VLDKLHEIGIEESSITR--------------------Schistosoma_mansoni/1-387          VAYTFDAGPNAFLLTESQNISV-VLKYLVECFGYTVEADSFVNNADKITIKCMNSNKYLKSchistosoma_japonicum/1-239        VAYTFDAGPNAFLLTESHNIEN-ILKYLVECFGRTVGVGD--------------------Bmal/1-393                         VAYTFDAGPNCCLFLESVNVPL-ILAAVNKYCKLRSDL----------------------Trichinella_spiralis/1-366         VAYSFDAGSNCFLLCLESEVEH-LLAYVCHYFCDTDTMPV--------------------Cele/1-365                         AAYTFDAGPNACVIVQKENASQ-FLKAVLQTIQVPSE-----------------------Atha/1-392                         VVGD---NSILKEAGLDGASGVENLQ--PPPEIKDNIGSQDQKGEVSYFI----------gi|327287778_Acaronlinensis/1-406  LKGL---PVEAVMPPEELLSAVVKDP--APGAIQYLLLTKPGPGPTIVNDGSCHLLGPDGgi|260794527_Bfloridae/1-406       VQGLP--DANRKDLPAELLNSVGLEP--SPGSVQYIIHTRAGQGPQVLTDPQQALLDEKGEcab/1-376                         LKGL---PVRPALLSDELKAALGMEP--TPGGIKYIIATQVG------------------Cfam/1-376                         LKGL---PVRPTPLSDEFKAALNVDP--IPGSIKYIIATQVG------------------MVD_Hs/1-376                       LKGL---QVRPAPLSAELQAALAMEP--TPGGVKYIIVTQVG------------------Mmus/1-376                         LKGL---QVAPVLLSDELKAALAVEP--SPGGVQYIIATQV-------------------Mdom/1-376                         LKGL---PVEPVELSEEVKSALPMEP--FPGGIRYIITTQVGPG----------------Oana/1-376                         LQGL---QVGSAPLSEELKSAMGPEL--TP------------------------------MVD_Acar/1-374                     LKGL---PVEAVMPPEELLSAVVKDP--APGAIQYLLL----------------------Xtro/1-376                         LKGL---PVGSAVLSEGLQSIVASDP--NPGGVRYIIYTKPGPGPTLS------------Ggal/1-366                         ------------------------------------------------------------Salmo/1-376                        LKGL---PVAPTTLSEELKQAIGMEP--MVKGICYIISTK--------------------Tnig/1-376                         IRGL---PIEGVSLPDALKQSIGLQP--MQNGISYVISTKAGPGPC--------------Drer/1-376                         FKGL---PVCPADLSEEMIRDINMKP--TPNGIRYMISTKAGP-----------------DC_Spur/1-371                      IRGL---DSRIHDIPQGLQCAMKRDP--NPGALKYIIHSKVGPGPQVVTDQELSLLDRNGSaccoglossus_kowalevskii/1-378     IRGLK--VDFKTDISQELKDVVPLEP--SPGAIKYVIST---------------------Trichoplax_adhaerens/1-374         IQGLSN-YSDVKEFPKEIENTICLDP--IPGSLTGIIHTRVGSGPRVILDDNE-------Aaeg/1-372                         IKGI---AVDLSELPEAQAEFTA--S--GNNLLKYIINTKVGEGPKRID-----------Dmel/1-374                         LRGL---PIPKVQDAESSKLDSLDVH--AKNAFRYIIHTKVGEGPKEL------------Tcas/1-372                         IRGL---PVKLKNSNNLRETLKIQTQ--TPNKLRYLIHTKIGEGPQILSEAS--------Arabidopsis_lyrata/1-392           VAGD---NSILKEAGLDGASGVENLQ--PPPEIKDNIGSQDQKGEVSYFI----------Atha2/1-385                        -------KSYVLGDTSIVKEAGLEGE--LPQGIKDKIGSQDQKGEVSYFI----------Arabidopsis_lyrata2/1-385          -------KSYVLGDTSIVKEAGLEGE--LPQGIKDKIGNQDQKGEVSYFI----------Ptri/1-389                         VIGD---KSILKDAGIEDIKDVEALP--PPPEIKDAQRCKGDVSYFI-------------Ricinus_communis/1-389             VLGD---KSILKDAGIEEMKDVESLP--APPEIKDAPRFKGDVSYFIC------------Vvin/1-392                         VIGD---KSILGDVGVEEMKDVEALP--PPPETKDQIPAQKQRGAVS-------------Zea_mays/1-389                     LVGD---KSILGVAGLHSMKDVEALP--APPETKIPDQKFKGDVSY--------------Ppat/1-392                         VHGD---QSILESAGVNSLKDIDSLS--APAEVAGIPNLQRIPGEVD-------------Smoe/1-388                         VVGSTE-LLEALGIDKLEDIRSLERP--IESPKRDEGHGELA------------------Salpingoeca_sp./1-378              VRGRSTTASDYSSTVDAAVREKISGPT-TADGVKYILHTGIGPG----------------Monosiga_brevicollis/1-311         VQGR---TTSVPEAEIAPELQSFPVA--EPDQIKYIIHTGIGDGPRVSQEHLINEAGERICapsaspora_owczarzaki/1-378        VRGFR--DVPAAPVDAATLQAINRSV--FADSLKYVMHTRVGSG----------------Lbic/1-373                         -------FKDNLGVFAQGAEVNAQAA--VPAGFNGLIHTRVG------------------Lbic2/1-373                        -------FKDNLGVFAQGAEVNAQAA--VPPGFNGLIHTRVG------------------Coprinopsis_cinerea/1-387          GGGVAD-INQVAQVPEGFNEKVAVVR--EVGAVKGLIH----------------------Schizophyllum_commune/1-382        A------NDQKKGAVVSGFNEAVAQK--WEGGVKGIIHTKIGD-----------------Cryptococcus_neoformans/1-375      -------QTGVRDLPEGFNTGVVREGGWEKGAVKGLIHTRVGDGP---------------Kluyveromyces_lactis/1-380         LSQF---NAKFDEIKDDVSFYLDSE---LHQGVTRVILTRVGPGP---------------Scer/1-379                         -------LEAFNHQFESSNFTARELDLELQKDVARVILTQVGSGP---------------Aory/1-379                         LK-----SFTAPGVALDKVDSRAVDV--LKDGVSRVI-----------------------Ylip/1-370                         -------KHQSVDAKKIDVDERWESE--LANGIQRVILTKVGGDPV--------------Schizosaccharomyces_pombe/1-368    -------QYGSSPVTVDSAAANI-----VSSGISRVILTRVG------------------Thalassiosira_pseudonana/1-335     ------------------------------------------------------------Phaeodactylum_tricornutum/1-372    -------NDEVLDPALVHAAEATGR-----------------------------------Ectocarpus_siliculosus/1-395       TSGAY--VNSAAALEAAREADACLPEGLYEACLKTGRTPTVGEV----------------Phytophthora_infestans/1-384       NRTPSQALLSRMEASLESGKTFKLPR--IPDSVKTMYVSRVGGGTRVLST----------Dictyostelium_purpureum/1-377      NVEK---IQSYKPNEKLQSLFTPETT--FASSLKYILHTKVGPGPQILSESE--------Dictyostelium_discoideum/1-377     NIPS---IENFVPSEKLASLYTPDTT--FVNSLKYILHTKVGPGPQILSESE--------Polysphondylium_pallidum/1-378     SNIEK--IEHYQPPKNIQTLFAPEVT--FADSLKYILHTKIGPGPQILDESES-------Naegleria_gruberi/1-386            VTE----MKQYTEMVESFKKSTSVKI--DADGLRYLIHTTVGYGPK--------------Nvec/1-377                         VQGI---PIPDSGIEKGLLNAIGMDP--NPSSIKY-------------------------Leishmania_major/1-372             -------LEKCKAYQLPASFEGLIDY--PKKSFEMLLQSPMGQGIVYL------------gi|149241991|pdb|2HKE|A/1-372      FFGDRELLEKVKVVSLPDEYKKLIDH--PKKPFEMLLQSPVGCGVKYLG-----------Encephalitozoon_intestinalis/1-302 ------------------------------------------------------------Encephalitozoon_cuniculi/1-303     ------------------------------------------------------------Tthe/1-384                         -------INDMSSLSQ--------------------------------------------Paramecium_tetraurelia/1-355       ----------------------------LFTKQKYII-----------------------Trichomonas_vaginalis/1-341        ------------------------AT--PCEGITCTYNE---------------------Schistosoma_mansoni/1-387          ITGI---SYDLSDEPLDQNLLKILPS--ISGGIRHLISTEVGSGPQLISFIH--------Schistosoma_japonicum/1-239        -------SMNTTDKFTVQCRDSNKYL--KVIGIRYSLSDTPLDQVIFMTI----------Bmal/1-393                         -------IERVAKYPAAFEYGNLRSL--VEEEQKNLVL----------------------Trichinella_spiralis/1-366         ICGW---TEKLRSDLSYGELDQIQ-R--LPGAVRIVVVSKVGSPPTVLH-----------Cele/1-365                         -------DLQVIGKELAEQFDVPAEP--TTKCSK--------------------------Atha/1-392                         -------gi|327287778_Acaronlinensis/1-406  QPRNSSGgi|260794527_Bfloridae/1-406       WPK----Ecab/1-376                         -------Cfam/1-376                         -------MVD_Hs/1-376                       -------Mmus/1-376                         -------Mdom/1-376                         -------Oana/1-376                         -------MVD_Acar/1-374                     -------Xtro/1-376                         -------Ggal/1-366                         -------Salmo/1-376                        -------Tnig/1-376                         -------Drer/1-376                         -------DC_Spur/1-371                      MPKKL--Saccoglossus_kowalevskii/1-378     -------Trichoplax_adhaerens/1-374         -------Aaeg/1-372                         -------Dmel/1-374                         -------Tcas/1-372                         -------Arabidopsis_lyrata/1-392           -------Atha2/1-385                        -------Arabidopsis_lyrata2/1-385          -------Ptri/1-389                         -------Ricinus_communis/1-389             -------Vvin/1-392                         -------Zea_mays/1-389                     -------Ppat/1-392                         -------Smoe/1-388                         -------Salpingoeca_sp./1-378              -------Monosiga_brevicollis/1-311         AS-----Capsaspora_owczarzaki/1-378        -------Lbic/1-373                         -------Lbic2/1-373                        -------Coprinopsis_cinerea/1-387          -------
